# Absolute quantification of the budding yeast transcriptome by means of competitive PCR between genomic and complementary DNAs

**DOI:** 10.1186/1471-2164-9-574

**Published:** 2008-11-29

**Authors:** Fumihito Miura, Noriko Kawaguchi, Mikio Yoshida, Chihiro Uematsu, Keiji Kito, Yoshiyuki Sakaki, Takashi Ito

**Affiliations:** 1 Department of Computational Biology, Graduate School of Frontier Sciences, University of Tokyo, Kashiwa 277-8561, Japan; 2 Institute for Bioinformatics Research and Development (BIRD), Japan Science and Technology Agency (JST), Tokyo 102-0081, Japan; 3 INTEC Systems Institute, Inc, Tokyo 136-0075, Japan; 4 Central Research Laboratory, Hitachi, Ltd, Kokubunji 185-8601, Japan; 5 RIKEN Genomic Sciences Center, Yokohama 230-0045, Japan

## Abstract

**Background:**

An ideal format to describe transcriptome would be its composition measured on the scale of absolute numbers of individual mRNAs per cell. It would help not only to precisely grasp the structure of the transcriptome but also to accelerate data exchange and integration.

**Results:**

We conceived an idea of competitive PCR between genomic DNA and cDNA. Since the former contains every gene exactly at the same copy number, it can serve as an ideal normalization standard for the latter to obtain stoichiometric composition data of the transcriptome. This data can then be easily converted to absolute quantification data provided with an appropriate calibration. To implement this idea, we improved adaptor-tagged competitive PCR, originally developed for relative quantification of the 3'-end restriction fragment of each cDNA, such that it can be applied to any restriction fragment. We demonstrated that this "generalized" adaptor-tagged competitive PCR (GATC-PCR) can be performed between genomic DNA and cDNA to accurately measure absolute expression level of each mRNA in the budding yeast *Saccharomyces cerevisiae*. Furthermore, we constructed a large-scale GATC-PCR system to measure absolute expression levels of 5,038 genes to show that the yeast contains more than 30,000 copies of mRNA molecules per cell.

**Conclusion:**

We developed a GATC-PCR method to accurately measure absolute expression levels of mRNAs by means of competitive amplification of genomic and cDNA copies of each gene. A large-scale application of GATC-PCR to the budding yeast transcriptome revealed that it is twice or more as large as previously estimated. This method is flexibly applicable to both targeted and genome-wide analyses of absolute expression levels of mRNAs.

## Background

An ideal way to describe transcriptome structure would be to elaborate on its composition based on the scale of absolute copy numbers of individual mRNAs per cell. Absolute quantification would help to precisely grasp the structure of the transcriptome. It would also accelerate exchange and sharing of data, which have remained difficult despite of considerable efforts to standardize the description format of transcriptome data in public databases [1]. While a variety of approaches can be undertaken for absolute quantification of individual transcripts, the basic principle of each approach can be classified as tag-counting, hybridization, or PCR.

Tag-counting methods include BodyMapping [2], SAGE [3], MPSS [4], CAGE [5], and GIS-PET [6]. Since these methods are random sampling approaches, a large enough number of tags have to be collected to deduce a statistically reliable portrait of the transcriptome, which can be converted to an absolute quantification data using an appropriate calibration. The recent advances in massively parallel sequencing technologies are expected to drastically improve the power of tag-counting approaches [7].

Hybridization-based methods include northern blot hybridization and those based on liquid-phase hybridization, such as nuclease protection assay. Although these classical methods are of high accuracy and reliability, tedious procedures inherent to them have hampered their applications to large-scale analyses. In terms of comprehensiveness, microarray or DNA chip hybridization is extremely powerful. A protocol was reported to measure absolute expression using microarray [8]. In this protocol, a cDNA sample of interest was hybridized in conjunction with a known amount of an oligonucleotide complementary to every feature on the array, and each signal derived from the sample was corrected by that from the oligonucleotide to be used as the abundance of the mRNA corresponding to that feature. Although this protocol can control for differences in target DNA quantity, spot morphology, and uneven hybridization, it cannot normalize labeling and sequence-specific hybridization differences among transcripts [8]. Another protocol that uses a set of "spike-in" calibration standards was proposed to estimate, but not to directly measure, endogenous transcript abundance [9].

Among the PCR-based approaches, real-time or kinetic PCR and competitive PCR are considered as the most sensitive and accurate ones [10]. Real-time PCR can be used as a high throughput assay, because it obviates any post-PCR steps such as gel electrophoresis. However, it is heavily influenced by the quality of the template and the stochastic nature of the first phases of amplification. In this context, competitive PCR is more robust than real-time PCR, although its application to a large-scale analysis had been hampered by the need to prepare a competitor standard for each target; a target and its competitor standard have to be co-amplified with a single primer pair to give products that differ in size.

This obstacle can be overcome by a unique method termed adaptor-tagged competitive (ATAC)-PCR [11]. In ATAC-PCR, double-stranded cDNAs are synthesized using a biotinylated oligo-dT primer and digested with a restriction enzyme, usually a 4-base cutter. The 3'-end restriction fragment of each cDNA is purified using avidin beads and ligated to an adaptor. The two cDNA samples to be compared are tagged with different adaptors. Notably, these adaptors share a common adaptor-specific primer (ASP) sequence, but its location is different between the two adaptors. The adaptor-tagged cDNAs are combined in a 1:1 ratio and used as a template for PCR using the ASP and a gene-specific primer (GSP). Accordingly, PCR products derived from the two cDNA templates differ in size and can be separated by gel electrophoresis. The changes in relative expression can be determined from the ratio of the two peaks. Remarkably, ATAC-PCR is totally free from laborious steps for the preparation of competitor standards, flexibly applicable to any gene set ranging from a small to a genome-wide scale, and proven highly accurate and sensitive in relative quantification of eukaryotic gene expression [11]. If an equimolar mixture of all cDNAs becomes available, ATAC-PCR can be readily used for absolute quantification of the transcriptome.

However, it is practically difficult to have such an "abundance-normalized" copy of the transcriptome. We thus pursued an alternative but practical solution that uses genomic DNA as a competitor standard in ATAC-PCR. Since genomic DNA isolated from non-dividing cells is guaranteed to contain every gene exactly at the same copy number (*i.e*., one copy per haploid genome), it can serve as an ideal standard to normalize different amplification efficiencies among amplicons. Competitive PCR between genomic DNA and cDNA on a genome-wide scale would immediately provide stoichiometric composition data of the transcriptome, which can be converted to absolute value with an appropriate calibration.

To achieve this goal, ATAC-PCR has to be improved so that it can be applied to any restriction fragment derived from either genomic or complementary DNAs, since the original protocol is applicable only to the 3'-end restriction fragment of each cDNA [11]. In addition, to convert the stoichiometric composition data to absolute quantification data, we need an independent method for data calibration. Finally, to describe the results in terms of copy number per cell, we have to determine the total amount of RNAs per cell.

In this study, we first investigated the methods 1) to fully extract and determine the amount of total RNAs, 2) to competitively amplify target gene fragment from genomic and complementary DNAs, and 3) to measure internal standards for the calibration of competitive PCR data. Then, we integrated these methods to precisely measure absolute expression levels of selected mRNAs in the budding yeast *Saccharomyces cerevisiae *to validate our approach. Finally, we constructed a large-scale competitive PCR system for genome-wide absolute quantification of the budding yeast transcriptome. The results revealed that the yeast transcriptome is composed of twice or more as many mRNAs as has been believed for the last 30 years.

## Results

### Total amount of RNA in budding yeast cells

To determine the exact amount of total RNAs and to extract them as thoroughly as possible, we tested several methods for RNA extraction from the budding yeast *S. cerevisiae*. A hot-phenol-based method [12] was found to be the best in both its yield and reproducibility. We also tested a classical destructive method to calculate the total amount of cellular RNAs [13], in which NaOH treatment was employed to hydrolyze all RNAs into mononucleotides. Both methods indicated that an average amount of cellular RNAs is 1.3~1.4 pg per cell (Table 1), although the destructive method always showed a slightly higher value than the hot-phenol method, as it did than a method based on radioisotope labeling [13]. We used the value obtained by the hot-phenol method hereinafter.

**Table 1:** Total amount of RNAs in a single yeast cell grown in YPD medium

Method	Culture #1	Culture #2	Culture #3	Average (SD)
Hot Phenol	1.32	1.34	1.29	1.32 (0.03)
NaOH/PCA	1.43	1.45	1.43	1.43 (0.01)

Total amount of cellular RNAs (pg per cell) was determined for three independent preparations of S288C cells grown in YPD medium using a modified hot-phenol method [12] and NaOH/PCA method [13].

### Generalized Adaptor-Tagged Competitive PCR (GATC-PCR)

**Figure 1:**
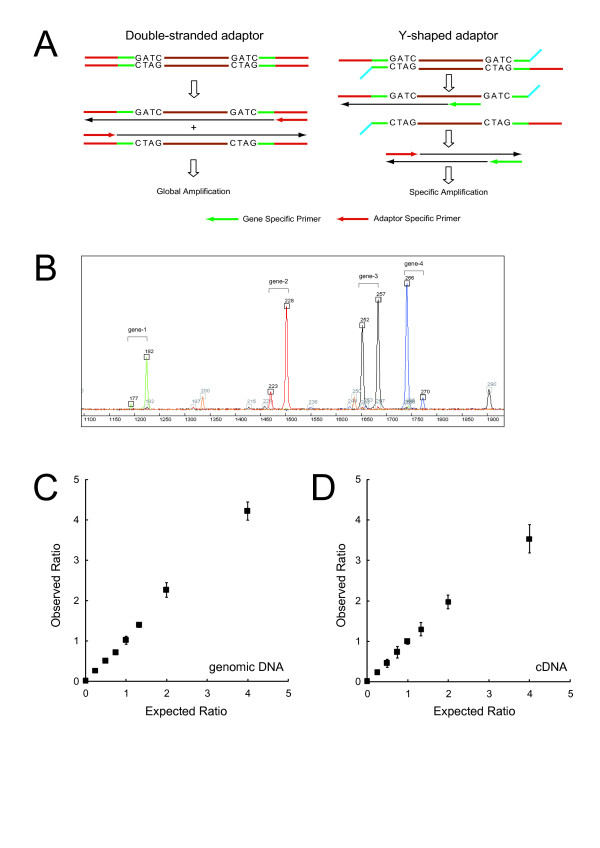
**Generalized Adaptor-Tagged Competitive PCR (GATC-PCR)**. (A) Gene-specific primer (GSP)-dependent amplification from Y-shaped adaptor-tagged template. (B) An example of GATC-PCR. Genomic DNA and cDNA digested with *Mbo *I were ligated with adaptor A/C and B/C (Table 2), respectively, and used for GATC-PCR. The products of four assays (blue, green, red, and black) and a size standard (orange) were separated on ABI 3730 Genetic Analyzer. The fast- and slow-migrating peaks of each pair correspond to the signals from genomic DNA and cDNA, respectively. (C) Linearity of GATC-PCR from genomic DNA templates. Genomic DNAs extracted from the wild and *gcn4*Δ cells were combined at appropriate ratios to prepare a series of genomic DNAs containing 0, 0.25, 0.5, 0.75, and 1 copy of *GCN4 *per haploid on average, digested with *Mbo *I, and ligated to the adaptors A/C and B/C (Table 2). Various combinations of the A/C- and B/C-tagged templates were mixed in a 1:1 ratio, while keeping the total amount equivalent to 3,000 haploid cells, and subjected to GATC-PCR using a *GCN4*-specific primer. (D) Linearity of GATC-PCR from cDNA templates. An experiment similar to the one shown in (C) was conducted using cDNAs, instead of genomic DNA, prepared from the wild and *gcn4*Δ cells.

To apply the principle of ATAC-PCR to any restriction fragment derived from either genomic DNA or cDNA, we employed a Y-shaped adaptor [14] (Table 2, Figure 1A). Note that this adaptor lacks any sequence complementary to ASP. Accordingly, priming from the ASP never occurs, unless the extended product from a GSP reaches the end of the adaptor to provide the sequence complementary to the ASP. Thus, in contrast to conventional double-stranded adaptors leading to global amplification of adaptor-tagged templates, the Y-shaped adaptor should restrict amplification to occur only from adaptor-tagged fragments to which a GSP hybridizes, thereby ensuring selective amplification dependent on the GSP (Figure 1A).

**Table 2:** Oligonucleotides used as primers and adaptors

Name	Sequence
Adaptor-specific primer	
M13RV	5' -**CAGGAAACAGCTATGAC**-3'
	
Adaptor	
Forward oligonucleotide	
A	5' -TGCACAATACTCACA**CAGGAAACAGCTATGAC**TGCGCTCACATCG-3'
B	5' -ACAATTCACA**CAGGAAACAGCTATGAC**TGCACTGCGCTCACATCG-3'
	
Reverse oligonucleotide	
C	5' -(PO_4_)GATCCGATGTGAGCGCCA-3'

The Y-shaped adaptor A/C or B/C was prepared by annealing equal molar amounts of the forward oligonucleotide A or B and the reverse oligonucleotide C, respectively. The sequence shared by the adaptor specific primer (ASP) and the adaptors is shown in bold. Complementary sequences between the forward and reverse oligonucleotides are underlined.

We succeeded in specific amplification of target genes from Y-shaped adaptor-tagged templates (Figure 1B). The products were separated and detected using an ABI 3730 multi-capillary DNA sequencer. Note that we used two Y-shaped adaptors to introduce 5-nt artificial fragment length polymorphism between cognate PCR products (Table 2; Figure 1B). Furthermore, the amplification was highly reproducible and quantitative from either genomic DNA or cDNA (Figure 1C, D). These results indicate that the introduction of the Y-shaped adaptors does not compromise the highly quantitative nature of ATAC-PCR. We termed this method as "generalized" adaptor-tagged competitive PCR or GATC-PCR.

**Figure 2:**
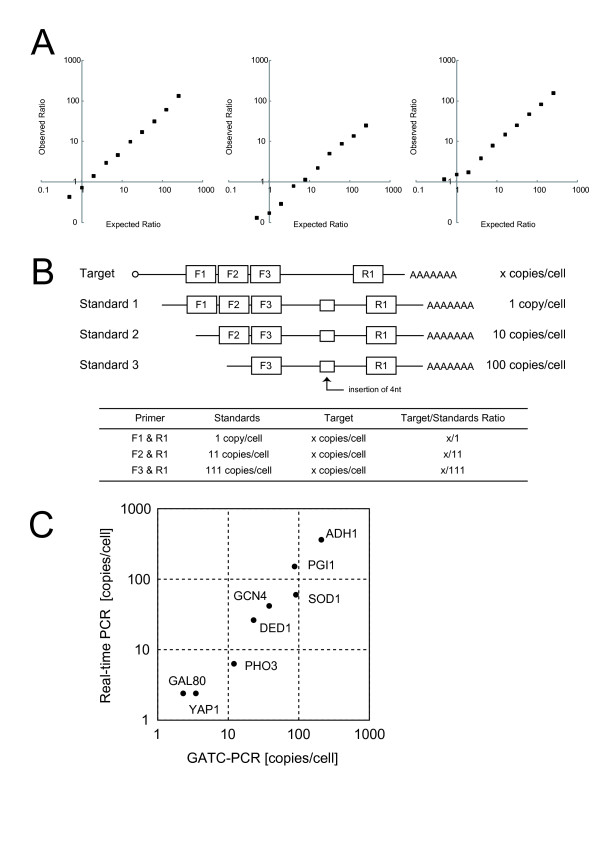
**Calibration of GATC-PCR between genomic DNA and cDNA**. (A) Competitive amplification of *GCN4 *between genomic DNA and cDNA. (B) Standard RNAs used for competitive PCR determination of mRNA copy number. (C) Comparison of absolute amounts of eight mRNAs determined by real-time PCR and GATC-PCR. For real-time PCR, we used each GSP for the first strand cDNA synthesis. The GATC-PCR data were calibrated by the competitive PCR quantification of *GCN4 *mRNA using the standard RNA set (Figure 2B, Table 3).

**Figure 3:**
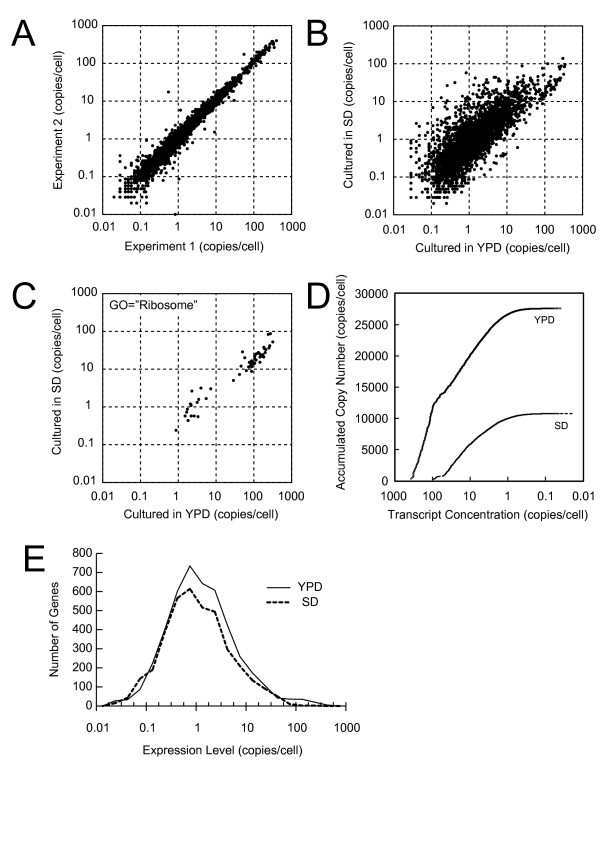
**Absolute quantification of the budding yeast transcriptome by large-scale GATC-PCR**. (A) Reproducibility of absolute quantification of the budding yeast transcriptome by GATC-PCR. Genome-wide GATC-PCR quantification was performed twice using the same total RNA sample labeled as #2 in Table 1. (B) ''Virtual R_0_t'' curve based on the merged expression data (Table 4). (C) Comparison of absolute mRNA levels between cells grown in YPD and SD media. Note that the plot includes 3,351 genes detectably expressed under both conditions but not those with undetectable levels of expression in either condition. (D) Comparison of absolute mRNA levels of genes with GO slim term ''ribosome'' between cells grown in YPD and SD media. (E) Distribution of transcript abundances in cells grown in YPD and SD media. The plot includes 3,351 genes detectably expressed in both conditions.

We next examined whether GATC-PCR can competitively amplify its target from a mixture of genomic and cDNA templates. For this experiment, we spiked different amounts of *in vitro *transcribed *GCN4 *mRNA to total RNAs extracted from a strain deleted for *GCN4*, thereby preparing a series of total RNA samples that differ solely in the concentration of *GCN4 *mRNA. We converted these RNAs into adaptor-tagged cDNA templates using the adaptor B/C (Table 2), mixed each of the cDNA templates with genomic DNA of the parental *GCN4 *strain tagged with the other adaptor A/C (Table 2), and amplified a *GCN4 *fragment by GATC-PCR. (In this experiment, we examined the expression levels ranging over three orders of magnitude, because the performance of the DNA sequencer limited the dynamic range of a single GATC-PCR assay to three to four orders of magnitude. However, we confirmed that GATC-PCR could cover mRNAs expressed at 0.01 to 100,000 copies per cell, when the results of assays at appropriate cDNA-genomic DNA mixing ratios were combined [Additional data file 1].) The results of amplification from three independent templates are shown in Figure 2A. Although the assay showed excellent linearity in every case, the levels of output varied. Nevertheless, the ratio between the signals for *GCN4 *and endogenous *ACT1 *was kept constant among the three experiments (not shown). We assumed that, although the three templates differ in yield, they share almost identical relative composition. This assumption was later confirmed by a large-scale measurement (Figure 3A). Thus, it is necessary to calibrate the results of GATC-PCR using an independent method.

### Calibration of GATC-PCR data

We designed a system to calibrate GATC-PCR data based on a competitive PCR that uses a unique series of standard RNAs (Figure 2B). The amplicon shared among these standards was made 4 nt longer than the one from the target, so that we could separate them by gel electrophoresis for quantification. Each standard RNA was prepared by *in vitro *transcription from a template prepared by modifying a full-length cDNA clone [15]. Accordingly, the 3' terminus of the each standard RNA including poly(A) tail is likely to be similar to that of target RNA, and we can expect a similar efficiency of reverse transcription between the standards and the target. The most prominent feature of the standards is that each of them is differentially 5'-truncated to retain a unique set of priming sites for the forward primers F1, F2, and F3 (Figure 2B). PCR with F1 and R1 primers generates a long product derived solely from standard 1; PCR using F2 and R1 generates a mid-length product derived from standards 1 and 2; PCR using F3 and R1 generates a short product derived from all of the standards. Thus, if the concentrations of the standards 1, 2, and 3 are set as 1, 10, 100 copies per cell, the amounts of the long, mid-length, and short products indicate the expression levels of 1, 11, and 111 copies per cell, respectively (Figure 2B). Among these points, one can use those close to the expression level of a target of interest for its quantification.

We used this scheme to measure the amount of *GCN4 *mRNA in three independent preparations of yeast cells grown in the rich medium YPD to find that each cell contains approximately 40 copies of *GCN4 *mRNA on average (Table 3). These results were consistent with those obtained by northern blot hybridization (Additional data file 2) and real-time PCR (Additional data file 3), both using an *in vitro *transcribed *GCN4 *mRNA as a standard. Thus, we decided to use the value obtained by this method for calibration of GATC-PCR data hereinafter.

**Table 3:** Copy number of *GCN4 *mRNA in a single yeast cell grown in YPD medium

Standard point	Culture #1	Culture #2	Culture #3
#1 (1 copy/cell)	ND	ND	ND
#2 (11 copies/cell)	38.7	45.4	47.3
#3 (111 copies/cell)	39.0	39.9	45.3
Average	38.9	42.7	46.3

Copy number of *GCN4 *mRNA per cell was determined for the same samples as those in Table 1 using the competitive PCR method using a set of *in vitro *transcribed RNA standards shown in Figure 2B.

### Absolute quantification of budding yeast mRNAs

To cover the wide expression range of the budding yeast genes [16], we conducted GATC-PCR using three different ratios of genomic DNA to cDNA (*i.e*., 1:1, 10:1, and 100:1), which would be suitable for quantification of mRNAs expressed around the levels of 1, 10, and 100 copies per cell, respectively. The data obtained at these three measuring points were averaged by putting more weight on the one having the smallest absolute value of log-converted cDNA/genomic DNA signal ratio. For calibration, we used the expression level of *GCN4 *mRNA determined as described above (Table 3). On the other hand, we used the same RNA for real-time PCR assays, for which we prepared *in vitro *transcribed RNAs as the standard of each gene to be measured. Then, we compared the results of GATC-PCR and real-time PCR for eight genes. As shown in Figure 2C, the results of GATC-PCR were found to be highly consistent with those of real-time PCR, providing a proof-of-principle for absolute quantification by GATC-PCR between genomic DNA and cDNA.

### Large-scale absolute quantification of the yeast transcriptome

To expand the GATC-PCR to a genome-wide scale, we designed GSPs for all of the 5,038 yeast genes bearing *Mbo *I sites in their open reading frames (ORFs). For this purpose, we fully exploited the SDSS algorithm that we had developed to design highly specific PCR primers based on the stability and uniqueness of 3'-end subsequences [17]. To evaluate how each primer can quantitatively work in the assay, we examined all the 5,038 primers in GATC-PCR using a series of templates, in each of which two genomic DNA samples tagged with different adaptors were mixed at a known ratio. We plotted the observed signal ratios against the theoretical ones to evaluate the performance of each primer (see Additional data files 4 and 5 for representative and all results, respectively) to find that 4,416 primers (*i.e*., 88% of 5,038 primers) worked satisfactorily (Additional data files 5 and 6). We thus decided to use these validated primers for genome-wide quantification.

To accelerate the analysis on the ABI 3730 DNA sequencer, we used four fluorescent dyes to label the ASP, combined four PCR products obtained using the four differentially labeled ASPs prior to electrophoresis, and detected them via the four optical channels of the sequencer (Figure 1B). We developed a program for automatic detection of peak pairs, each of which consists of a peak derived from the genomic DNA and a peak derived from the cDNA. Note that the former is 5 nt shorter than the latter because of the design of the two adaptors used for tagging of genomic and complementary DNA templates (Table 2). We also developed a software tool to reveal the identity of each peak.

We used the same RNA sample twice for genome-wide measurement to examine overall reproducibility of GATC-PCR as a technical replicate. As shown in Figure 3A, the GATC-PCR system reproducibly measured the expression levels for most genes (R^2 ^= 0.985). From inspection of individual off-diagonal data points, we learned that error in peak identification was the major cause of irreproducibility (data not shown). When the signal ratio between the longer and shorter amplicons was high, a fraction of the former occasionally renatured and migrated faster or very closely to the latter, thereby making the quantification inaccurate.

We used the three independent RNA preparations from the cells grown in YPD medium for genome-wide quantification as biological replicates (Table 4, Additional data file 6). We successfully measured more than 90% of the 4,416 genes, for which we could design validated GSPs, each time and 97% of them (*i.e*., 4,287 genes) at least once in the three measurements. For the remaining 129 genes, we failed to detect their expression in all of the three measurements. Total copy number of mRNAs for these 4,287 genes was estimated to be 27,539 per cell. If we assume no expression of the 129 genes and extrapolate the result to all of the 5,795 yeast genes, which include 4,717 verified and 1,078 uncharacterized ORFs but not 812 dubious ones [18], the total copy number of mRNAs is estimated to be 36,139 per cell. This is remarkably different from the prevailing estimate or 15,000 copies per cell, which is based on a classical R_0_t analysis of the cells grown in a rich medium [19] (see Discussion). The data also illustrate a skewed composition of the yeast transcriptome (Figure 3B): transcripts ranked within the top 1.6%, 9.4%, and 59% in terms of abundance exist at the level exceeding 100, 10, and 1 copies per cell to share 43%, 73%, and 96% of total mRNAs, respectively.

**Table 4:** Total copy number of mRNAs quantified by large-scale GATC-PCR

	Culture #1	Culture #2	Culture #3	Merged
Number of genes quantified	4,055	3,976	4,101	4,287
Total copy number of mRNAs	25,841	23,142	27,372	27,539

Total copy number of mRNAs was calculated for the 4,416 genes whose GSPs were validated for quantification (Additional data file 6). The same RNA samples as those shown in Table 1 were used for GATC-PCR quantification. Note that we excluded the genes whose expression levels were called to be zero from the calculation, because we could not distinguish between the genes that we failed to amplify from the cDNA template and those that were expressed at undetectable levels. Thus, the number of "genes quantified" in the table should be regarded as the most conservative or minimum estimate. To minimize the effect of failed assays, we merged the three datasets to cover 4,287 genes measured at least once in the three measurements and calculated the total copy number from the average copy number of each of these genes.

We next intended to measure the absolute levels of mRNAs in the same strain grown in the minimum medium SD. However, we found that the amount of total RNAs extracted with the hot-phenol procedure (0.5 pg/cell) from these cells was much smaller than the one measured by the NaOH/PCA method (0.9 pg/cell) for unknown reasons. Since none of the methods that we tested improved the yield, we used RNAs extracted with the hot-phenol method for the experiment but used the value obtained by the NaOH/PCA method for the calculation of copy number. We successfully measured the expression levels of 3,381 genes and calibrated the data to conclude that the total copy number of mRNAs for these genes was 10,766 per cell (Additional data file 6).

We compared the absolute expression levels of 3,351 genes between the cells grown in YPD and SD media (Figure 3C, E). As expected from the total copy numbers, most genes were less abundantly expressed in cells grown in SD medium than in those grown in YPD medium. The down-regulation was particularly prominent for a group of genes that were expressed above the level of 100 copies per cell under the rich condition (Figures 3C, Additional data file 7), which could account for the decrease of ~15,000 copies per cell, or ~40% of the transcripts measured in the cells grown in YPD medium. These genes could also be recognized as a shoulder in the plot between the number of genes and absolute expression levels in the cells under the rich condition (Figure 3E). The majority of these genes are, as expected, those encoding ribosomal proteins (Figure 3D). On the other hand, a group of genes were more abundantly expressed under the poor condition than the rich condition. Genes involved in biosynthesis of amino acids were significantly enriched in this group.

## Discussion

The absolute copy number of each macromolecule can be considered a gold standard for the description of a biological system. It is thus ideal to describe the composition of a transcriptome on the scale of absolute number of each RNA species. This would not only accelerate data exchange and integration but provide a precise picture of the transcriptome, which may be overlooked in a ratiometric or relative quantification analysis. For instance, global mRNA changes occur in heat-shocked mammalian cells, those under serum-starvation, and yeast cells at the stationary phase. In these cases, conventional microarray analysis, which usually assumes no changes in the total amount of mRNA, resulted in the false classification of thousands of genes as differentially expressed [20]. Even small global shifts in mRNA populations were shown to mislead the interpretation of ratiometric expression data, unless carefully designed external controls are included [20,21]. By contrast, absolute quantification is highly sensitive to global changes and totally free from these concerns, thereby greatly helping one to precisely grasp and properly interpret the transcriptome.

For absolute quantification of transcriptome, an equimolar mixture of mRNAs for all genes would serve as an ideal standard. Although theoretically possible, it is practically difficult to prepare such an "abundance-normalized transcriptome" from expression-ready full-length cDNA clones. Thus, we employed an alternative method that uses genomic DNA isolated from non-dividing cells as a standard, since every gene is contained in exactly the same copy number or abundance-normalized in the genome.

We used an ATAC-PCR approach as the method of quantification, because it is highly accurate, robust, totally free from tedious steps for the preparation of individual competitor standards, and hence applicable to various scales of quantification. Since the original ATAC-PCR can be applied only to the 3'-end restriction fragment, we introduced Y-shaped adaptors in the protocol to develop GATC-PCR that can analyze any restriction fragment derived from either genomic or complementary DNAs with high accuracy (Figure 1). Using the GATC-PCR and a unique competitive PCR assay for data calibration, we succeeded in accurate absolute quantification of yeast mRNAs (Figure 2).

Genomic DNA has been used as a cohybridization standard in ratiometric microarrays to accelerate data comparison [22,23]. However, it has remained unclear whether single-stranded cDNAs and double-stranded genomic DNAs can be labeled and hybridized with the same efficiency. No rigorous examination of accuracy has thus far been provided in terms of absolute quantification. By contrast, DNA fragments to be compared in GATC-PCR, one from genomic DNA and the other from cDNA, share the same structure except for a subtle sequence difference in the tagged adaptors (Table 2), thereby being free from the problems of differential efficiency in labeling and hybridization. Indeed, the accuracy of this approach was demonstrated in this study.

GATC-PCR can be flexibly applied to quantification experiments of any gene set ranging from a small to a genome-wide scale. However, its application to a genome-wide analysis has several practical drawbacks: it requires a large number of primers to be designed and synthesized, PCR, and capillary electrophoresis runs. In terms of cost, it is desirable to reduce the volume in current PCR (5 μl), because it is too much for detection by the DNA sequencer, which requires only 5 nl of the reaction. However, we failed to reduce the volume further, while maintaining a sufficient success rate of PCR. In addition, current throughput of the capillary DNA sequencer is not sufficient for multiple measurements. Another concern would be its applicability to organisms bearing larger and intron-rich genomes. We assume it plausible, at least, for genes bearing appropriate exonic restriction sites, since we confirmed that highly specific primers designed by appropriate algorithms [15,24] enabled GATC-PCR between human genomic DNA and cDNA (data not shown). It should be noted that, beside absolute quantification of mRNAs, GATC-PCR would also be applicable to relative quantification of splicing variants as well as copy number variation in genomic DNAs.

We applied the GATC-PCR to absolute quantification of the budding yeast transcriptome. The results indicate that the transcriptome contains ~36,000 mRNAs under a nutrient-rich condition (Figure 3, Table 4). This result was striking, because the yeast has been generally believed to contain 15,000 copies of poly(A)^+ ^RNA molecules per cell based on the result of R_0_t analysis [19]. Why do these two estimates differ so greatly?

To answer this question, we revisited the process in which the previous estimate was obtained [19]. The study divided mass quantity of poly(A)^+ ^RNAs by that of an average mRNA to obtain the total copy number. The amount of poly(A)^+ ^RNAs was assumed to be 1.5% of total RNAs from the result of R_0_t analysis, and the average length of mRNAs was assumed to be 1,500 nt. Notably, the amount of total RNAs used for the calculation was not experimentally determined but deduced from the total amount of DNA by using an assumption that the ratio of RNA to DNA in the yeast is 50 [19]. According to this classical assumption, the amount of total RNAs per cell was calculated to be 0.75 pg, which is remarkably smaller than the 1.3 pg reported in recent literature [25] and obtained by our measurements (Table 1). We also examined the average length of mRNA. The average length of budding yeast ORFs in the Saccharomyces Genome Database [18] is 1,340 nt. The median lengths of 5'/3'-untranslated regions revealed by tiling array hybridization [26] and RNA-Seq [27] were reported to be 68 nt/91 nt and 50 nt/104 nt, respectively. Thus, the average length of mRNA is 1,500 nt, coincident with the previous estimate [19]. However, it should be noted that a significant negative correlation was observed between expression level and ORF length in the budding yeast [28]. Indeed, the average size of the yeast ORFs can be as short as 1,123 nt and 1,083 nt when weighted by the copy numbers calculated from signal intensity of high-density DNA microarray [29] and GATC-PCR results, respectively. Thus, the average length of mRNA should be regarded as ~1,250 nt rather than 1,500 nt.

Taken together, the previous study used 1.7-fold smaller mass amount of mRNAs and 1.2-fold larger mRNA size, thereby leading to an approximately 2-fold underestimation of total copy number. In other words, interpretation of the classical R_0_t results with correct parameters led to an estimated total copy number of ~31,000, which is in good agreement with the estimate from GATC-PCR or ~36,000.

It is intriguing to note that the total number of mRNAs in the yeast transcriptome can be larger than the current estimate, because recent studies uncovered the presence of unannotated RNA species including intergenic transcripts, antisense transcripts, and those starting from inside the ORFs [15,26,27,30]. A single GSP conceivably quantifies not only the target mRNA but its antisense and/or internally primed transcripts, although these RNAs are generally several-fold less abundant than mRNA. For more accurate quantification or, at least, careful interpretation of the data, it is crucial to elucidate the structure of each transcription unit in the yeast genome.

We used the GATC-PCR to compare transcriptome between the yeast cells grown under rich and poor conditions or in YPD and SD media, respectively (Figure 3). Notably, the sizes of the transcriptome differ significantly between the two conditions: the total copy number of mRNA under the poor condition is approximately half of that under the rich condition, largely because of a drastic decrease in a group of the most abundant mRNAs encoding ribosomal proteins. These results well illustrate how dynamically the yeast transcriptome can be reorganized upon environmental changes. Absolute quantification would precisely detect such changes, thereby leading to proper interpretation of the transcriptome.

## Conclusion

We developed a method termed GATC-PCR to accurately measure absolute expression levels of mRNAs by means of competitive amplification of genomic and cDNA copies of each gene fragment. Absolute quantification of mRNAs using a large-scale GATC-PCR analysis indicated that the budding yeast transcriptome is composed of twice or more as many mRNAs as previously estimated. The method would be flexibly applicable to both targeted and genome-wide analyses of absolute expression levels of mRNAs.

## Methods

### Preparation of yeast total RNA

A single colony of strain S288C was inoculated into 10 ml of YPD (1%(w/v) yeast extract/2%(w/v) Bacto peptone/2%(w/v) glucose) and grown with shaking at 30°C for overnight. To prepare cells under a rich condition, they were resuspended in 100 ml of YPD at an OD_600 _of 0.1 and grown at 30°C for 6 hrs. To prepare cells under a poor condition, the cells grown overnight in YPD media were washed with ddH_2_O, resuspended in 100 ml of SD (0.67%(w/v) yeast nitrogen base without amino acids/2%(w/v) glucose) at an OD_600 _of 0.5, and grown at 30°C for 6 hrs. The cells were collected by centrifugation, resuspended in ddH_2_O, aliquoted in microtubes (400 μl), frozen in liquid nitrogen, and stored at -80°C until use. The number of cells was directly counted using a hematocytometer.

Total RNA was extracted using a hot-phenol method [12] with some modifications. To the 400-μl cell suspension described above, 100 μl of 5× lysis buffer (50 mM Tris-HCl, pH 7.5/50 mM EDTA/2.5%(w/v) SDS) and 500 μl of water-saturated phenol were added and mixed well on a shaker at 65°C for 1 hr. The tubes were chilled on ice for 5 min and centrifuged for phase separation. While the aqueous phase was saved in another tube, the phenol phase was mixed with 500 μl of 1× lysis buffer and shaked at 65°C for 1 hr. The second aqueous phase was combined with the first one and extracted once with water-saturated phenol and once with chloroform. The RNAs was precipitated by adding isopropanol to the aqueous phase, rinsed with 75%(v/v) ethanol, and dissolved in ddH_2_O. To remove contaminating genomic DNA, the RNA was treated with RNase-free DNase I (Promega) and purified with TRIzol reagent (Invitrogen) according to the manufacturer's instruction. The concentration of RNA was determined by measuring OD_260 _on spectrophotometer based on an assumption that one OD_260 _unit corresponds to 40 ng/μl of RNA.

Total amount of cellular RNAs was also determined using selective extraction of ribonucleotides by NaOH [13] with some modifications. To the 400-μl cell suspension, 100 μl of 1.2 N perchloric acid (PCA) was added and the obtained mixture was placed in ice-cold water for 1 hr. Following centrifugation, the supernatant was removed and the cell pellet was washed again with 500 μl of 0.25 N PCA. Following careful removal of residual PCA solution, the cell pellet was resuspended in 300 μl of 0.3 N NaOH and incubated at 37°C for 1 hr. After neutralization with adding 150 μl of 1.2 N PCA, the concentration of RNA was determined from OD_260 _and a standard curve obtained from the measurement of various known amounts of purified yeast RNA subjected to the same NaOH/PCA treatment.

### Preparation of genomic DNA

An S288C isogenic strain lacking mitochondria genome was obtained by three passages of the cells on YPD plate containing 50 μg/ml ethidium bromide. This strain was cultured in YPD at 30°C for overnight, diluted to an OD_600 _of 0.5 in 200 ml of YPD, and cultured at 30°C for 8 hrs. At the end of culture, the cell growth reached to stationary phase. Cells were collected by centrifugation, and resuspended in 10 ml of buffer Y1 (1.0 M Sorbitol/100 mM EDTA/14 mM β-mercaptoethanol) containing 0.1 mg/ml Zymolyase 100T (Seikagaku Co) and incubated at 30°C for 30 min. From the spheroplasts collected by centrifugation, genomic DNA was isolated with Genomic-tip 500 kit (Qiagen) according to the manufacturer's instruction. The concentration of purified genomic DNA was determined by measuring OD_260 _based on an assumption that one OD_260 _unit corresponds to 50 ng/μl of DNA.

### Preparation of standard RNA

We prepared *in vitro *transcribed RNAs to be used as internal standards for template preparation of GATC-PCR and standards for quantification by real-time PCR. We constructed a plasmid bearing each template sequence downstream of T7 promoter, linearized it by restriction enzyme digestion, and used 1 μg of the linearized plasmid as a template for T7 RiboMax Express Large Scale RNA Production System (Promega). We used RNeasy Mini kit (Qiagen) to purify the *in vitro *transcribed RNAs according to the manufacturer's instruction. Concentration of each RNA standard was determined by measuring the OD_260 _of alkaline-hydrolyzed RNA and subsequent calculation based on its base composition and the molar extinction coefficients (*i.e*., 15,400, 7,400, 11,500, and 8,700 for adenosine, cytosine, guanosine, and uridine, respectively).

### Preparation of double-stranded cDNA

Less than 5 μg of total RNAs were mixed with an appropriate amount of the standard RNAs and 0.5 μg of dT_18 _primer, adjusted to 10 μl with ddH_2_O, heat-denatured at 70°C for 5 min, and chilled on ice. Following the addition of 10 μl of 2× RT solution (a mixture of 4 μl of 5× first strand buffer, 2 μl of 0.1 M DTT, 1 μl of ddH_2_O, 1 μl of 10 mM dNTPs, and 2 μl of SuperScript III RNaseH^- ^reverse transcriptase [Invitrogen]) to the denatured RNAs, the reverse transcription was proceeded by a three-step incubation at 25°C for 15 min, 42°C for 60 min, and 70°C for 10 min. To this solution kept on ice, 20 μl of the second strand buffer (100 mM Tris-HCl, pH 6.9/23 mM MgCl_2_/450 mM KCl/0.75 mM β-NAD^+^/50 mM (NH_4_)_2_SO_4_), 60 μl of ddH_2_O, and 1 μl of 10 mM dNTPs were added to prepare the second strand synthesis reaction, which was initiated by adding 10 units of *E. coli *DNA polymerase I, 1 unit of RNase H, and 1 unit of *E. coli *DNA ligase, followed by incubation at 16°C for 3 hrs. The reaction was terminated by heating at 70°C for 10 min.

### Preparation of adaptor-tagged templates

The double-stranded cDNA solution (~100 μl) was mixed with 50 μl of digestion buffer (100 mM Tris-HCl, pH 8.3/20 mM MgCl_2_/100 mM KCl). For genomic DNA template, up to 1 μg of genomic DNA was dissolved in 150 μl of 1× K buffer (TAKARA, 20 mM Tris-HCl, pH 8.5/10 mM MgCl_2_/1 mM DTT/100 mM KCl). These cDNA and genomic DNA were digested with 10 units of *Mbo *I (TAKARA) at 37°C for 1 hr. Following heat inactivation of *Mbo *I at 70°C for 10 min, each tube was chilled on ice and supplemented with 50 μl of ligation buffer (100 mM Tris-HCl, pH 6.9/4 mM ATP/4 mM DTT/10 mM MgCl_2_) and 100 pmol of an adaptor mix (Table 2). The ligation reaction was initiated by addition of 10 Weiss units of T4 DNA ligase and completed by an overnight incubation at 16°C. The enzymes were inactivated by adding 50 μl of 0.1 M EDTA to the solution.

The templates tagged with different adaptors prepared as above were combined and mixed with 1.8-volume of AMpure (Agencourt). After rinsing SPRI beads with 75%(v/v) ethanol twice, we eluted the templates with 10 mM Tris-acetate (pH 8.0) and stored at -20°C.

### GATC-PCR

In relative quantification (Figure 1C, D), templates tagged with adaptors A/C and B/C (Table 2) were mixed at various ratios, while keeping the total amount constant or equivalent to 3,000 haploid cells per assay. In absolute quantification, genomic DNA and cDNA templates were tagged with adaptor A/C and B/C (Table 2), respectively, and combined at three different ratios, namely 1:1, 10:1, and 100:1. To achieve these ratios, genomic DNA templates equivalent to 3,000, 9,486, and 30,000 haploid cells were combined with cDNA templates equivalent to 3,000, 948, and 300 haploid cells, respectively.

For a single round of genome-wide quantification, we started with 39 μg of total RNAs (*i.e*., 3 × 10^7 ^cells) to prepare the cDNA template. On the other hand, we dispensed 5 pmol of each GSP to each well of 384-well PCR plates, which were dried and stored in an air desiccator until use. An aliquot (5 μl) of PCR master-mix solution, or 1× PCR buffer (Invitrogen) containing 0.2 mM dNTPs, 4.0 mM MgCl_2_, 0.2 μM of 5'-fluorescence-labeled ASP (Table 2), appropriate amount of adaptor-tagged template DNAs, and 0.02 unit/μl of Platinum Taq DNA polymerase (Invitrogen) or TaqHS DNA polymerase (Takara), was dispensed to each well of the PCR plate, which was then subjected to a thermal-cycling composed of a prefacing incubation at 95°C 1 min, 40 cycles of 3-step thermal incubation at 95°C for 20 sec, 60°C for 30 sec, and 72°C for 30 sec, followed by a final incubation at 72°C for 5 min using ABI 9700 thermal cycler.

### Electrophoresis

The GATC-PCR products were separated on ABI 3730 genetic analyzer. We developed a program termed SizeCaller to calculate both size and intensity of each peak in the electropherogram. The calculated peaks were exported to a software tool termed AQUOS that we developed to reveal the identity of each peak.

### Gene-specific primers (GSPs)

We had developed an algorithm termed SDSS to extract highly specific PCR primers [15]. Using the SDSS algorithm, we designed GSPs for 5,038 yeast ORFs bearing *Mbo *I sites. To avoid splitting of a peak induced by partial addition of dA to the 3' end of PCR product by Taq DNA polymerase, we adequately added dG or dGdG to the 5' end of each GSP to make it either 5'-dGdG-, 5'-dGdA-, or 5'-dAdA-, thereby maximizing the efficiency of dA addition (Uematsu C, unpublished data). Nucleotide sequence of each GSP is listed in Additional data file 6. These GSPs were synthesized by Genset or Proligo Japan KK (Kyoto).

### Real-time PCR

We performed real-time PCR using ABI 7000 sequence detection system and Platinum SYBR Green qPCR SuperMix UDG kit (Invitrogen) according to the manufacturer's instruction.

## Authors' contributions

FM conceived of the study, participated in its design, developed the GATC-PCR and programs for primer design and signal quantification, established the large-scale GATC-PCR system, analyzed the data, and drafted the manuscript. NK carried out the large-scale GATC-PCR. MY developed the peak identification software. CU contributed to the development of the primer design program. KK and YS participated in the design of the study. TI conceived of the study, participated in its design and coordination, analyzed the data, and drafted the manuscript. All authors read and approved the final manuscript.

## Supplementary Material

Additional file 1 **Dynamic range of GATC-PCR.** We prepared another series of total RNA samples that differ solely in the concentration of *GCN4 *mRNA, as we did for the experiment shown in Figure 2A. The concentrations of *GCN4 *mRNA in this series ranged from 0.001 to 100,000 copies per cell, thereby covering a much wider concentration range than the one used in Figure 2A. For GATC-PCR, we adjusted the mixing ratio between cDNA and genomic DNA according to the levels of *GCN4 *mRNA as indicated in the inset table. The measured copy number of *GCN4 *mRNA in each sample was plotted against the expected value. We failed to detect any specific signal from the sample corresponding to 0.001 copies per cell, which contained 10 copies of *GCN4 *mDNA. Click here for file

Additional file 2 **Quantification of *GCN4 *mRNA by northern blot hybridization.** (A) Northern blot hybridization of *GCN4 *mRNA. We used an *in vitro *transcribed *GCN4 *RNA as a standard. The standard RNA was transcribed from a plasmid derived from a full-length cDNA clone for *GCN4*, thereby retaining almost the same 3'-end structure as natural *GCN4 *mRNA. Lanes 1 to 6 contained the standard RNAs corresponding to 0, 20, 40, 80, and 160 copies per cell, respectively, whereas lane 7 contained the total RNA labeled as #1 in Table 1. The standard RNAs were loaded with total RNA extracted from a *gcn4*Δ strain so that lanes 1 to 7 contained the same amount of RNAs. (B) Quantification of northern blot hybridization signals. Chemiluminescent signals of the standard RNA in (A) were quantified using LAS-3000 (Fujifilm) and plotted against their amounts to obtain a standard curve. The arrow indicates the signal of the sample (lane 7), which corresponds to approximately 40 copies per cell. Click here for file

Additional file 3 **Quantification of *GCN4 *mRNA by real-time PCR.** (A) Real-time quantitative PCR of *GCN4 *mRNA. We used an *in vitro *transcribed *GCN4 *RNA as a standard. The template for *in vitro *transcription was prepared by PCR amplification of entire *GCN4 *ORF followed by cloning into pCR2.1-Topo vector (Invitrogen) according to the manufacturer's instructions. The standards and the sample or total yeast RNA labeled as #1 in Table 1 were spiked into RNAs extracted from *E. coli *strain DH5α to adjust the environment for reverse transcription and PCR amplification. (B) The C_t _values were plotted against log-converted expression level to obtain a linear standard curve. The arrow indicates the C_t _value for *GCN4 *mRNA in the sample, which corresponds to 40.1 copies per cell. Click here for file

Additional file 4 **Typical examples for GSP evaluation.** (A) Performance of GSPs in GATC-PCR quantification. Each GSP was examined in GATC-PCR from a series of templates, in each of which genomic DNAs tagged with adaptors A/C and B/C (Table 2) were mixed at a known ratio. Obtained ratios were plotted against expected ratios. Approximately 88% of the primers (*e.g*., SCM0001) gave satisfactory results, whereas 8% worked unsatisfactorily (*e.g*., SCM0053 and SCM0129) and 4% failed to obtain enough data points for plotting. Data for all primers are listed in Additional data file 5. (B) Frequency of primers in terms of the slope of the regression line. (C) Frequency of primers in terms of the intercept of the regression line. Click here for file

Additional file 5 **Evaluation of 5,038 GSPs.** A mini-website to browse plots similar to those shown in Additional data file 4 for all the 5,038 GSPs. Click here for file

Additional file 6 **GATC-PCR data.** GATC-PCR data for three independent samples of cells grown in YPD medium and a sample of cells grown in SD medium are summarized in a single table with information on each GSP. The minus sign (-) in the expression level column indicates a failed assay in which the signal from genomic DNA template was not detected. Click here for file

Additional file 7 **Comparison of transcriptome between cells grown in YPD and SD media.** (A) Distribution of transcript abundances in cells grown in YPD and SD media. The plot is similar to that in Figure 3C but contains every gene quantified in each condition. (B) Distribution of transcript abundances for genes to which GO slim term "Ribosome" is assigned. Data are shown for both cells grown in YPD and SD media. The plot includes every gene in the category successfully quantified in each condition. (C) "Virtual R_0_t" curve for 3,351 genes detectably expressed in cells grown in YPD and SD media. Click here for file
