# Supplementary material for: Absolute quantification of the budding yeast transcriptome by means of competitive PCR between genomic and complementary DNAs
Source: BMC Genomics. 2008 Nov 29;9:574. doi: 10.1186/1471-2164-9-574 (PMC2612024; doi:10.1186/1471-2164-9-574)
Supplement: Additional file 1 — Dynamic range of GATC-PCR. We prepared another series of total RNA samples that differ solely in the concentration of GCN4 mRNA, as we did for the experiment shown in Figure 2A. The concentrations of GCN4 mRNA in this series ranged from 0.001 to 100,000 copies per cell, thereby covering a much wider concentration range than the one used in Figure 2A. For GATC-PCR, we adjusted the mixing ratio between cDNA and genomic DNA according to the levels of GCN4 mRNA as indicated in the inset table. The measured copy number of GCN4 mRNA in each sample was plotted against the expected value. We failed to detect any specific signal from the sample corresponding to 0.001 copies per cell, which contained 10 copies of GCN4 mDNA. [file 1471-2164-9-574-S1.pdf]

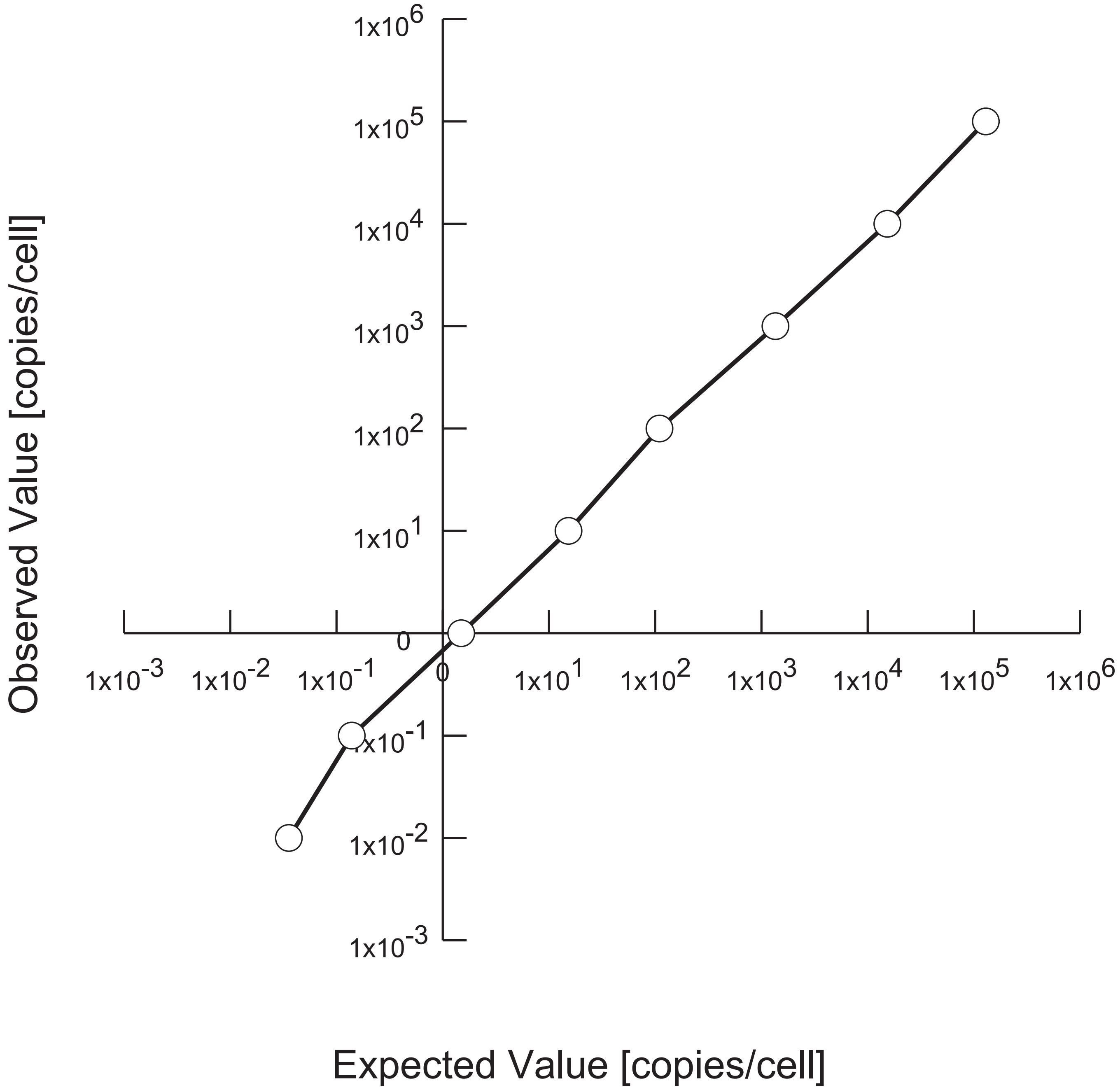

Concentration of GCN4 mRNA (copies/cell)	$1x10^{-3}$	$1x10^{-2}$	$1x10^{-1}$	$1x10^{0}$	$1x10^{1}$	$1x10^{2}$	$1x10^{3}$	$1x10^{4}$	$1x10^{5}$
Amount of cDNA (cell equivalents/tube)	10000	10000	10000	10000	1000	100	10	1	0.1
Amount of genomic DNA (cell equivalents/tube)	10	100	1000	10000	10000	10000	10000	10000	10000

Miura F. et al Additional Data File 1
