# Supplementary figures and images for: Absolute quantification of the budding yeast transcriptome by means of competitive PCR between genomic and complementary DNAs (part 1 of 3)
Source: BMC Genomics. 2008 Nov 29;9:574. doi: 10.1186/1471-2164-9-574 (PMC2612024; doi:10.1186/1471-2164-9-574)

**A**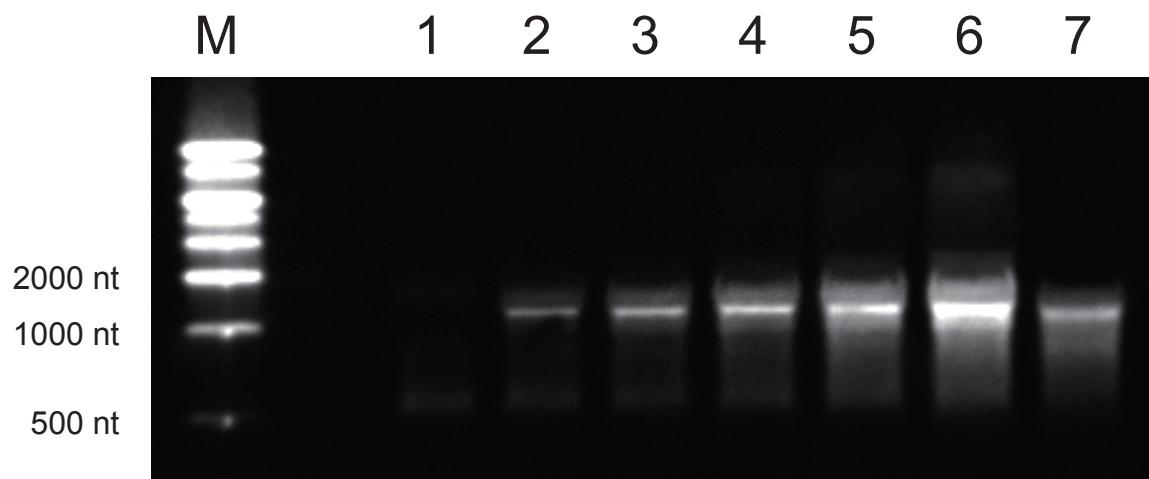**B**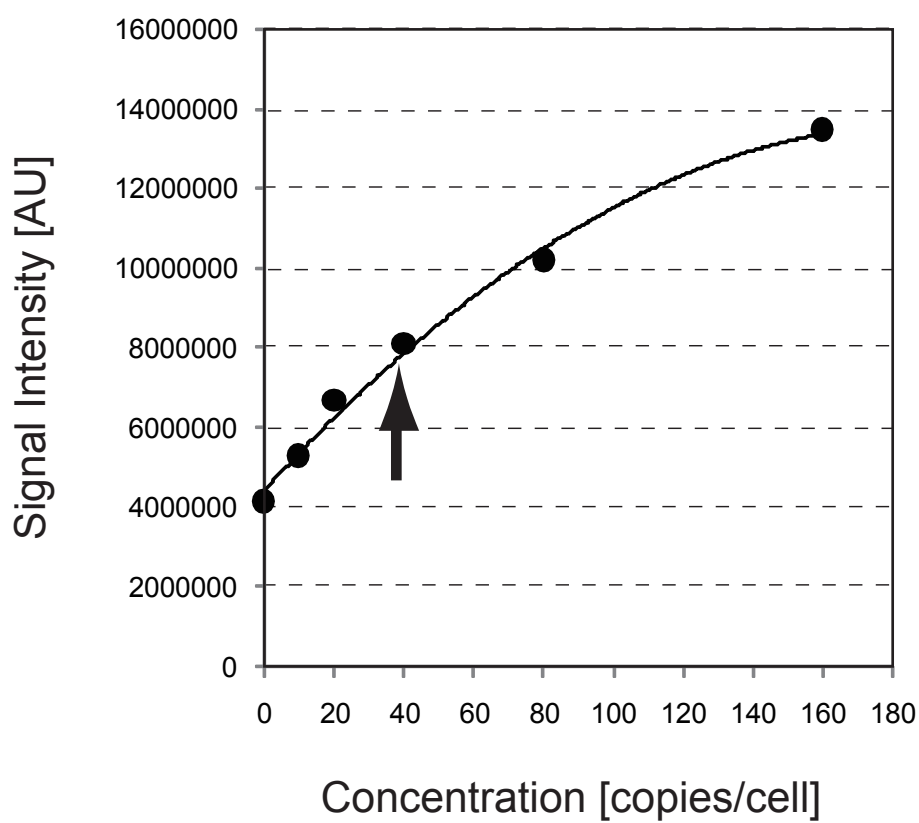

Supplement: Additional file 2 — Quantification of GCN4 mRNA by northern blot hybridization. (A) Northern blot hybridization of GCN4 mRNA. We used an in vitro transcribed GCN4 RNA as a standard. The standard RNA was transcribed from a plasmid derived from a full-length cDNA clone for GCN4, thereby retaining almost the same 3'-end structure as natural GCN4 mRNA. Lanes 1 to 6 contained the standard RNAs corresponding to 0, 20, 40, 80, and 160 copies per cell, respectively, whereas lane 7 contained the total RNA labeled as #1 in Table 1. The standard RNAs were loaded with total RNA extracted from a gcn4Δ strain so that lanes 1 to 7 contained the same amount of RNAs. (B) Quantification of northern blot hybridization signals. Chemiluminescent signals of the standard RNA in (A) were quantified using LAS-3000 (Fujifilm) and plotted against their amounts to obtain a standard curve. The arrow indicates the signal of the sample (lane 7), which corresponds to approximately 40 copies per cell. [file 1471-2164-9-574-S2.pdf]

A

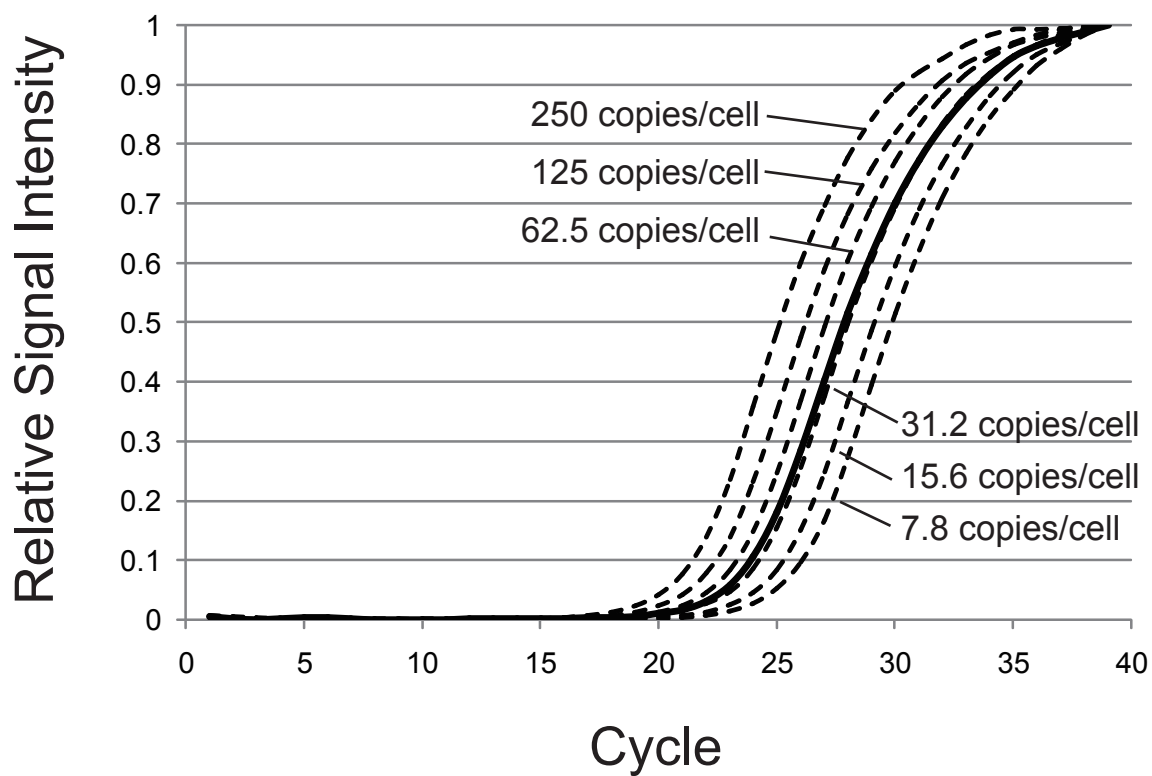

B

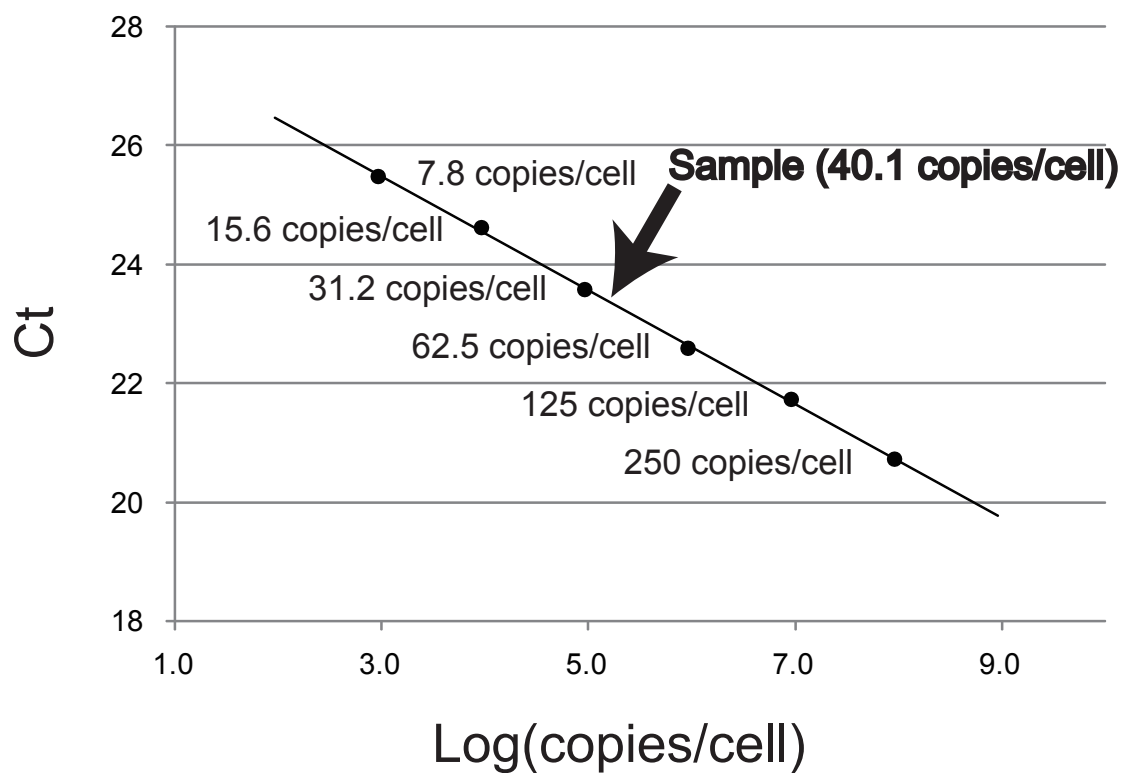

Supplement: Additional file 3 — Quantification of GCN4 mRNA by real-time PCR. (A) Real-time quantitative PCR of GCN4 mRNA. We used an in vitro transcribed GCN4 RNA as a standard. The template for in vitro transcription was prepared by PCR amplification of entire GCN4 ORF followed by cloning into pCR2.1-Topo vector (Invitrogen) according to the manufacturer's instructions. The standards and the sample or total yeast RNA labeled as #1 in Table 1 were spiked into RNAs extracted from E. coli strain DH5α to adjust the environment for reverse transcription and PCR amplification. (B) The Ct values were plotted against log-converted expression level to obtain a linear standard curve. The arrow indicates the Ct value for GCN4 mRNA in the sample, which corresponds to 40.1 copies per cell. [file 1471-2164-9-574-S3.pdf]

A

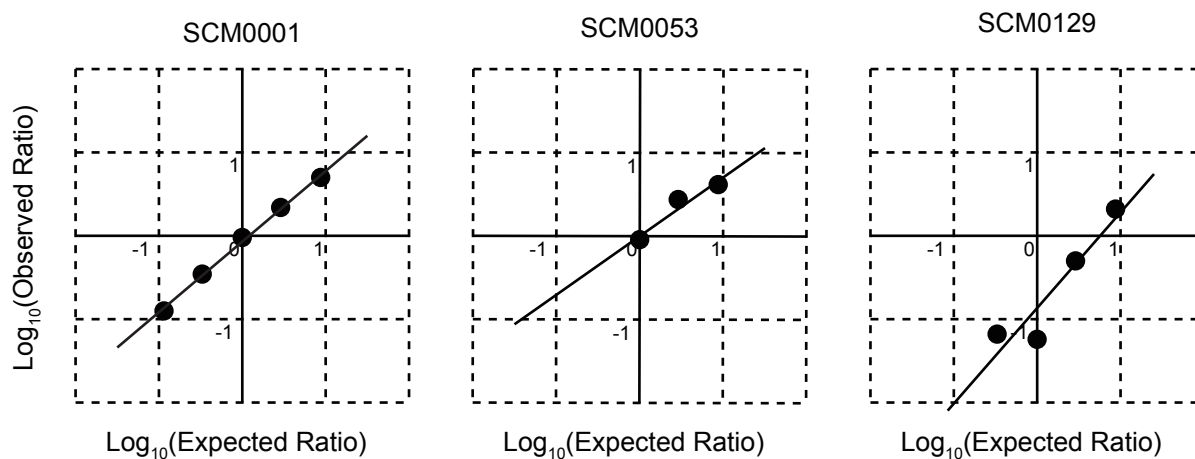

B

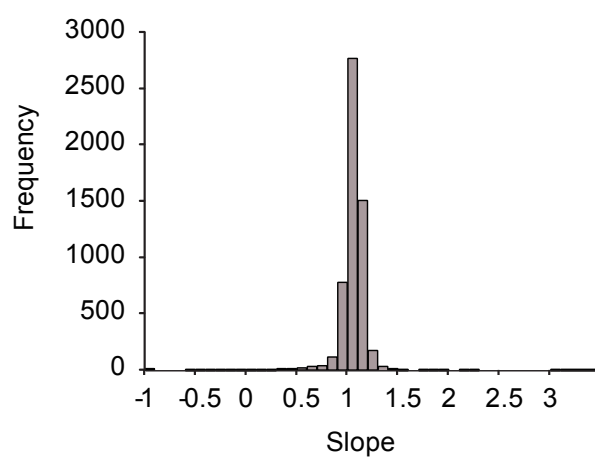

C

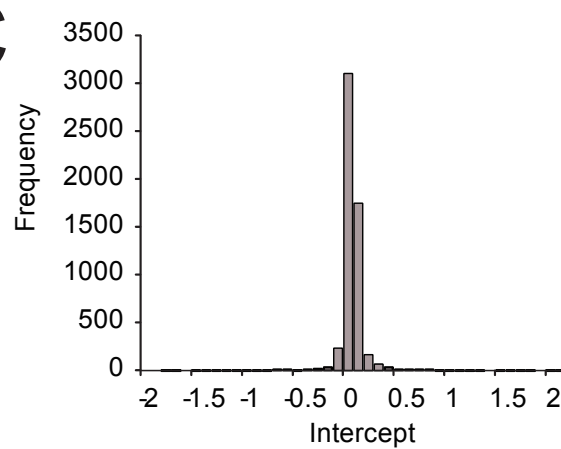

Supplement: Additional file 4 — Typical examples for GSP evaluation. (A) Performance of GSPs in GATC-PCR quantification. Each GSP was examined in GATC-PCR from a series of templates, in each of which genomic DNAs tagged with adaptors A/C and B/C (Table 2) were mixed at a known ratio. Obtained ratios were plotted against expected ratios. Approximately 88% of the primers (e.g., SCM0001) gave satisfactory results, whereas 8% worked unsatisfactorily (e.g., SCM0053 and SCM0129) and 4% failed to obtain enough data points for plotting. Data for all primers are listed in Additional data file 5. (B) Frequency of primers in terms of the slope of the regression line. (C) Frequency of primers in terms of the intercept of the regression line. [file 1471-2164-9-574-S4.pdf]

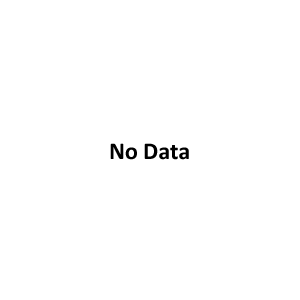

Supplement: Additional file 5 — Evaluation of 5,038 GSPs. A mini-website to browse plots similar to those shown in Additional data file 4 for all the 5,038 GSPs. [file 1471-2164-9-574-S5.zip › image/blank.png]

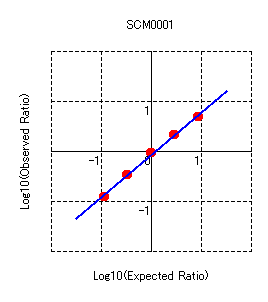

Supplement: Additional file 5 — Evaluation of 5,038 GSPs. A mini-website to browse plots similar to those shown in Additional data file 4 for all the 5,038 GSPs. [file 1471-2164-9-574-S5.zip › image/SCM0001.png]

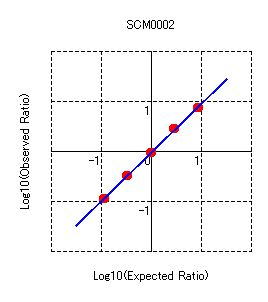

Supplement: Additional file 5 — Evaluation of 5,038 GSPs. A mini-website to browse plots similar to those shown in Additional data file 4 for all the 5,038 GSPs. [file 1471-2164-9-574-S5.zip › image/SCM0002.png]

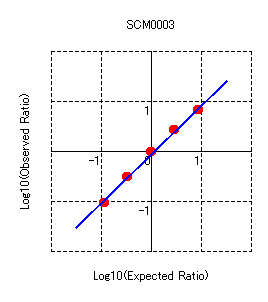

Supplement: Additional file 5 — Evaluation of 5,038 GSPs. A mini-website to browse plots similar to those shown in Additional data file 4 for all the 5,038 GSPs. [file 1471-2164-9-574-S5.zip › image/SCM0003.png]

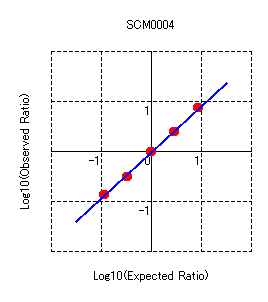

Supplement: Additional file 5 — Evaluation of 5,038 GSPs. A mini-website to browse plots similar to those shown in Additional data file 4 for all the 5,038 GSPs. [file 1471-2164-9-574-S5.zip › image/SCM0004.png]

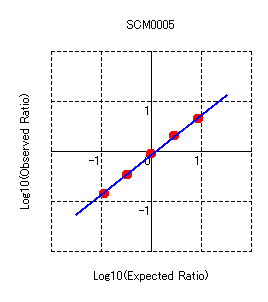

Supplement: Additional file 5 — Evaluation of 5,038 GSPs. A mini-website to browse plots similar to those shown in Additional data file 4 for all the 5,038 GSPs. [file 1471-2164-9-574-S5.zip › image/SCM0005.png]

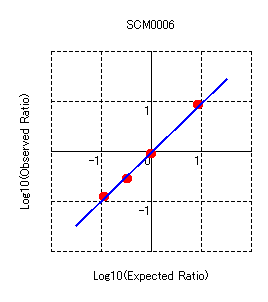

Supplement: Additional file 5 — Evaluation of 5,038 GSPs. A mini-website to browse plots similar to those shown in Additional data file 4 for all the 5,038 GSPs. [file 1471-2164-9-574-S5.zip › image/SCM0006.png]

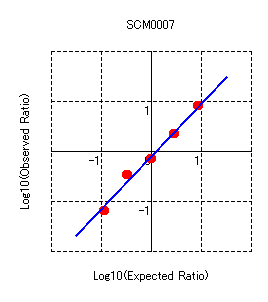

Supplement: Additional file 5 — Evaluation of 5,038 GSPs. A mini-website to browse plots similar to those shown in Additional data file 4 for all the 5,038 GSPs. [file 1471-2164-9-574-S5.zip › image/SCM0007.png]

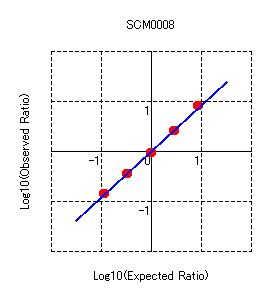

Supplement: Additional file 5 — Evaluation of 5,038 GSPs. A mini-website to browse plots similar to those shown in Additional data file 4 for all the 5,038 GSPs. [file 1471-2164-9-574-S5.zip › image/SCM0008.png]

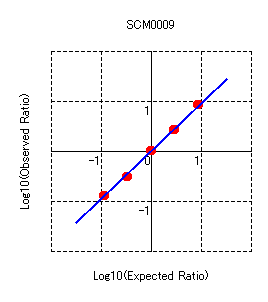

Supplement: Additional file 5 — Evaluation of 5,038 GSPs. A mini-website to browse plots similar to those shown in Additional data file 4 for all the 5,038 GSPs. [file 1471-2164-9-574-S5.zip › image/SCM0009.png]

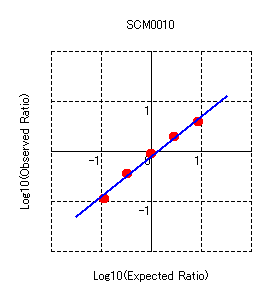

Supplement: Additional file 5 — Evaluation of 5,038 GSPs. A mini-website to browse plots similar to those shown in Additional data file 4 for all the 5,038 GSPs. [file 1471-2164-9-574-S5.zip › image/SCM0010.png]

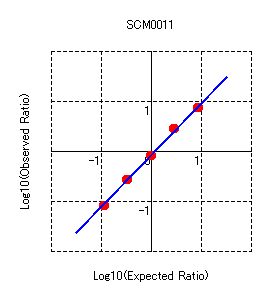

Supplement: Additional file 5 — Evaluation of 5,038 GSPs. A mini-website to browse plots similar to those shown in Additional data file 4 for all the 5,038 GSPs. [file 1471-2164-9-574-S5.zip › image/SCM0011.png]

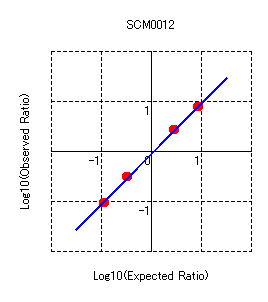

Supplement: Additional file 5 — Evaluation of 5,038 GSPs. A mini-website to browse plots similar to those shown in Additional data file 4 for all the 5,038 GSPs. [file 1471-2164-9-574-S5.zip › image/SCM0012.png]

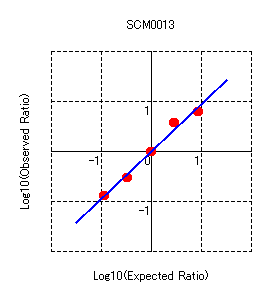

Supplement: Additional file 5 — Evaluation of 5,038 GSPs. A mini-website to browse plots similar to those shown in Additional data file 4 for all the 5,038 GSPs. [file 1471-2164-9-574-S5.zip › image/SCM0013.png]

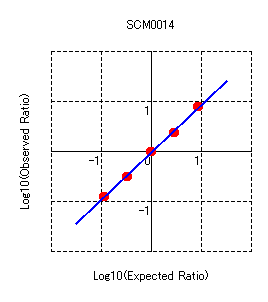

Supplement: Additional file 5 — Evaluation of 5,038 GSPs. A mini-website to browse plots similar to those shown in Additional data file 4 for all the 5,038 GSPs. [file 1471-2164-9-574-S5.zip › image/SCM0014.png]

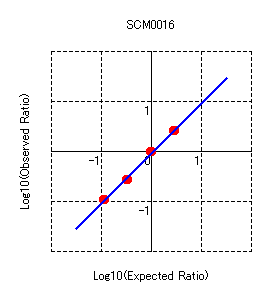

Supplement: Additional file 5 — Evaluation of 5,038 GSPs. A mini-website to browse plots similar to those shown in Additional data file 4 for all the 5,038 GSPs. [file 1471-2164-9-574-S5.zip › image/SCM0016.png]

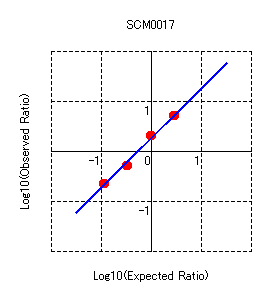

Supplement: Additional file 5 — Evaluation of 5,038 GSPs. A mini-website to browse plots similar to those shown in Additional data file 4 for all the 5,038 GSPs. [file 1471-2164-9-574-S5.zip › image/SCM0017.png]

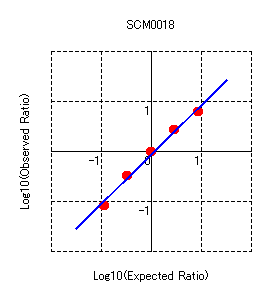

Supplement: Additional file 5 — Evaluation of 5,038 GSPs. A mini-website to browse plots similar to those shown in Additional data file 4 for all the 5,038 GSPs. [file 1471-2164-9-574-S5.zip › image/SCM0018.png]

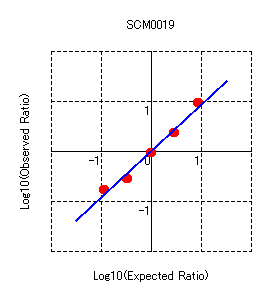

Supplement: Additional file 5 — Evaluation of 5,038 GSPs. A mini-website to browse plots similar to those shown in Additional data file 4 for all the 5,038 GSPs. [file 1471-2164-9-574-S5.zip › image/SCM0019.png]

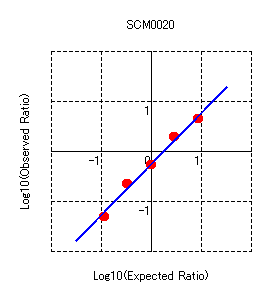

Supplement: Additional file 5 — Evaluation of 5,038 GSPs. A mini-website to browse plots similar to those shown in Additional data file 4 for all the 5,038 GSPs. [file 1471-2164-9-574-S5.zip › image/SCM0020.png]

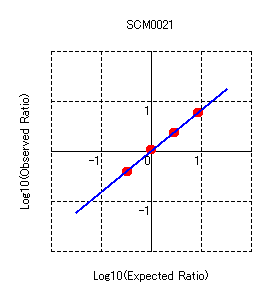

Supplement: Additional file 5 — Evaluation of 5,038 GSPs. A mini-website to browse plots similar to those shown in Additional data file 4 for all the 5,038 GSPs. [file 1471-2164-9-574-S5.zip › image/SCM0021.png]

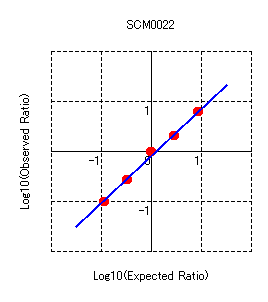

Supplement: Additional file 5 — Evaluation of 5,038 GSPs. A mini-website to browse plots similar to those shown in Additional data file 4 for all the 5,038 GSPs. [file 1471-2164-9-574-S5.zip › image/SCM0022.png]

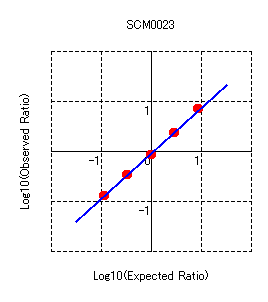

Supplement: Additional file 5 — Evaluation of 5,038 GSPs. A mini-website to browse plots similar to those shown in Additional data file 4 for all the 5,038 GSPs. [file 1471-2164-9-574-S5.zip › image/SCM0023.png]

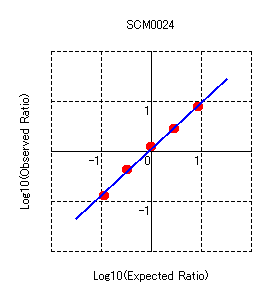

Supplement: Additional file 5 — Evaluation of 5,038 GSPs. A mini-website to browse plots similar to those shown in Additional data file 4 for all the 5,038 GSPs. [file 1471-2164-9-574-S5.zip › image/SCM0024.png]

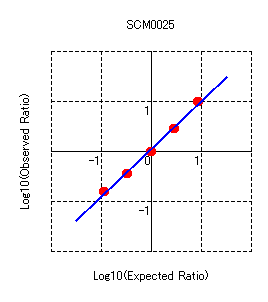

Supplement: Additional file 5 — Evaluation of 5,038 GSPs. A mini-website to browse plots similar to those shown in Additional data file 4 for all the 5,038 GSPs. [file 1471-2164-9-574-S5.zip › image/SCM0025.png]

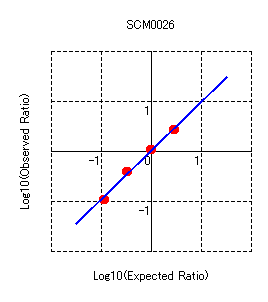

Supplement: Additional file 5 — Evaluation of 5,038 GSPs. A mini-website to browse plots similar to those shown in Additional data file 4 for all the 5,038 GSPs. [file 1471-2164-9-574-S5.zip › image/SCM0026.png]

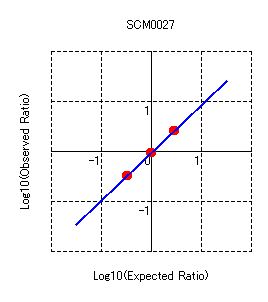

Supplement: Additional file 5 — Evaluation of 5,038 GSPs. A mini-website to browse plots similar to those shown in Additional data file 4 for all the 5,038 GSPs. [file 1471-2164-9-574-S5.zip › image/SCM0027.png]

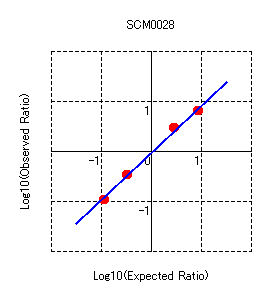

Supplement: Additional file 5 — Evaluation of 5,038 GSPs. A mini-website to browse plots similar to those shown in Additional data file 4 for all the 5,038 GSPs. [file 1471-2164-9-574-S5.zip › image/SCM0028.png]

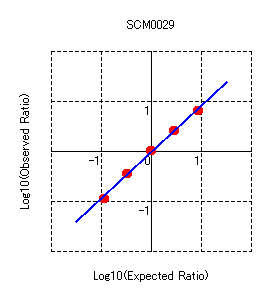

Supplement: Additional file 5 — Evaluation of 5,038 GSPs. A mini-website to browse plots similar to those shown in Additional data file 4 for all the 5,038 GSPs. [file 1471-2164-9-574-S5.zip › image/SCM0029.png]

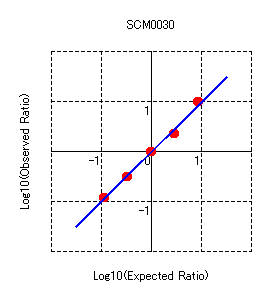

Supplement: Additional file 5 — Evaluation of 5,038 GSPs. A mini-website to browse plots similar to those shown in Additional data file 4 for all the 5,038 GSPs. [file 1471-2164-9-574-S5.zip › image/SCM0030.png]

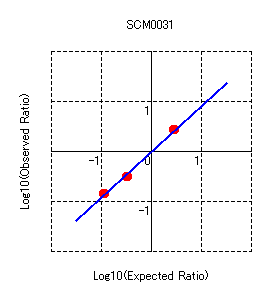

Supplement: Additional file 5 — Evaluation of 5,038 GSPs. A mini-website to browse plots similar to those shown in Additional data file 4 for all the 5,038 GSPs. [file 1471-2164-9-574-S5.zip › image/SCM0031.png]

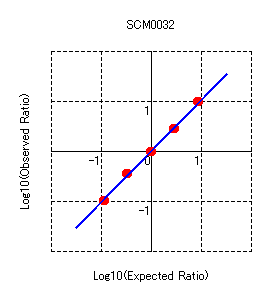

Supplement: Additional file 5 — Evaluation of 5,038 GSPs. A mini-website to browse plots similar to those shown in Additional data file 4 for all the 5,038 GSPs. [file 1471-2164-9-574-S5.zip › image/SCM0032.png]

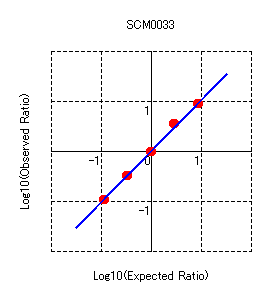

Supplement: Additional file 5 — Evaluation of 5,038 GSPs. A mini-website to browse plots similar to those shown in Additional data file 4 for all the 5,038 GSPs. [file 1471-2164-9-574-S5.zip › image/SCM0033.png]

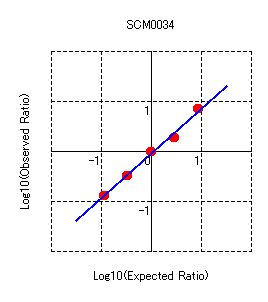

Supplement: Additional file 5 — Evaluation of 5,038 GSPs. A mini-website to browse plots similar to those shown in Additional data file 4 for all the 5,038 GSPs. [file 1471-2164-9-574-S5.zip › image/SCM0034.png]

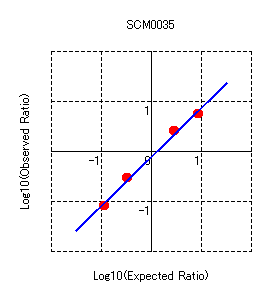

Supplement: Additional file 5 — Evaluation of 5,038 GSPs. A mini-website to browse plots similar to those shown in Additional data file 4 for all the 5,038 GSPs. [file 1471-2164-9-574-S5.zip › image/SCM0035.png]

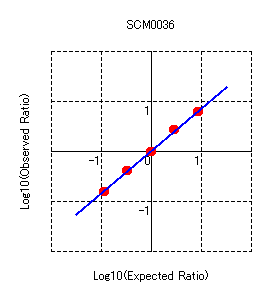

Supplement: Additional file 5 — Evaluation of 5,038 GSPs. A mini-website to browse plots similar to those shown in Additional data file 4 for all the 5,038 GSPs. [file 1471-2164-9-574-S5.zip › image/SCM0036.png]

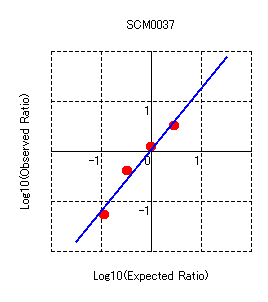

Supplement: Additional file 5 — Evaluation of 5,038 GSPs. A mini-website to browse plots similar to those shown in Additional data file 4 for all the 5,038 GSPs. [file 1471-2164-9-574-S5.zip › image/SCM0037.png]

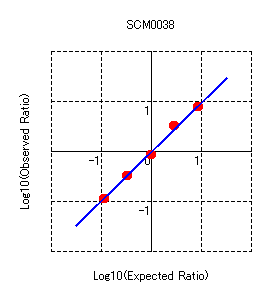

Supplement: Additional file 5 — Evaluation of 5,038 GSPs. A mini-website to browse plots similar to those shown in Additional data file 4 for all the 5,038 GSPs. [file 1471-2164-9-574-S5.zip › image/SCM0038.png]

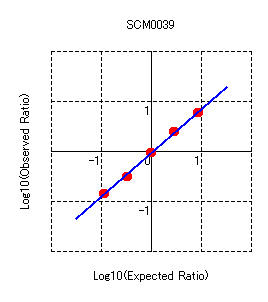

Supplement: Additional file 5 — Evaluation of 5,038 GSPs. A mini-website to browse plots similar to those shown in Additional data file 4 for all the 5,038 GSPs. [file 1471-2164-9-574-S5.zip › image/SCM0039.png]

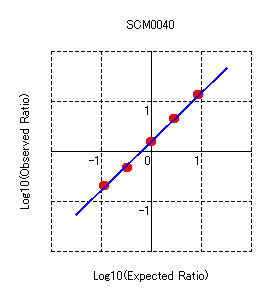

Supplement: Additional file 5 — Evaluation of 5,038 GSPs. A mini-website to browse plots similar to those shown in Additional data file 4 for all the 5,038 GSPs. [file 1471-2164-9-574-S5.zip › image/SCM0040.png]

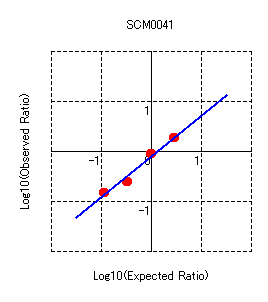

Supplement: Additional file 5 — Evaluation of 5,038 GSPs. A mini-website to browse plots similar to those shown in Additional data file 4 for all the 5,038 GSPs. [file 1471-2164-9-574-S5.zip › image/SCM0041.png]

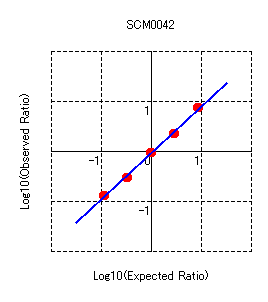

Supplement: Additional file 5 — Evaluation of 5,038 GSPs. A mini-website to browse plots similar to those shown in Additional data file 4 for all the 5,038 GSPs. [file 1471-2164-9-574-S5.zip › image/SCM0042.png]

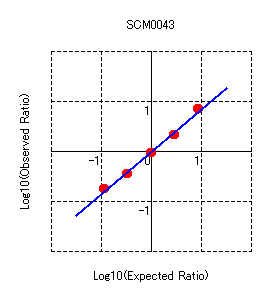

Supplement: Additional file 5 — Evaluation of 5,038 GSPs. A mini-website to browse plots similar to those shown in Additional data file 4 for all the 5,038 GSPs. [file 1471-2164-9-574-S5.zip › image/SCM0043.png]

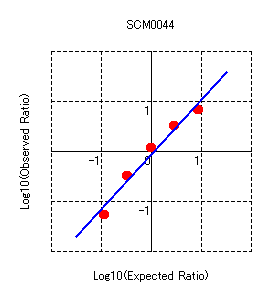

Supplement: Additional file 5 — Evaluation of 5,038 GSPs. A mini-website to browse plots similar to those shown in Additional data file 4 for all the 5,038 GSPs. [file 1471-2164-9-574-S5.zip › image/SCM0044.png]

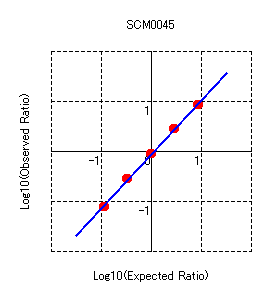

Supplement: Additional file 5 — Evaluation of 5,038 GSPs. A mini-website to browse plots similar to those shown in Additional data file 4 for all the 5,038 GSPs. [file 1471-2164-9-574-S5.zip › image/SCM0045.png]

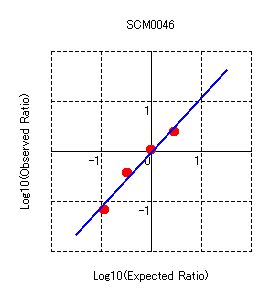

Supplement: Additional file 5 — Evaluation of 5,038 GSPs. A mini-website to browse plots similar to those shown in Additional data file 4 for all the 5,038 GSPs. [file 1471-2164-9-574-S5.zip › image/SCM0046.png]

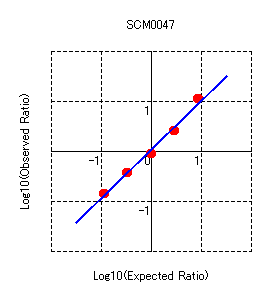

Supplement: Additional file 5 — Evaluation of 5,038 GSPs. A mini-website to browse plots similar to those shown in Additional data file 4 for all the 5,038 GSPs. [file 1471-2164-9-574-S5.zip › image/SCM0047.png]

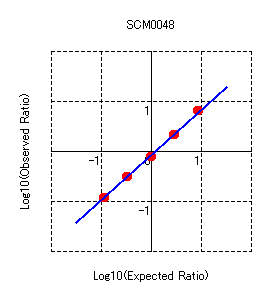

Supplement: Additional file 5 — Evaluation of 5,038 GSPs. A mini-website to browse plots similar to those shown in Additional data file 4 for all the 5,038 GSPs. [file 1471-2164-9-574-S5.zip › image/SCM0048.png]

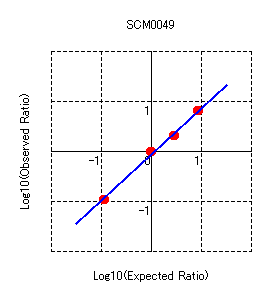

Supplement: Additional file 5 — Evaluation of 5,038 GSPs. A mini-website to browse plots similar to those shown in Additional data file 4 for all the 5,038 GSPs. [file 1471-2164-9-574-S5.zip › image/SCM0049.png]

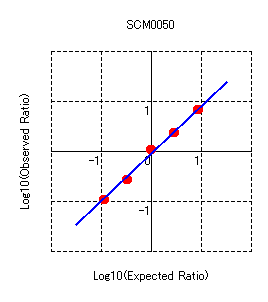

Supplement: Additional file 5 — Evaluation of 5,038 GSPs. A mini-website to browse plots similar to those shown in Additional data file 4 for all the 5,038 GSPs. [file 1471-2164-9-574-S5.zip › image/SCM0050.png]

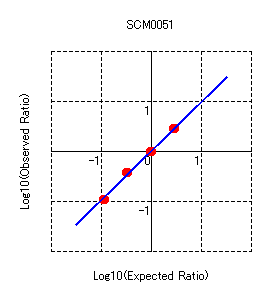

Supplement: Additional file 5 — Evaluation of 5,038 GSPs. A mini-website to browse plots similar to those shown in Additional data file 4 for all the 5,038 GSPs. [file 1471-2164-9-574-S5.zip › image/SCM0051.png]

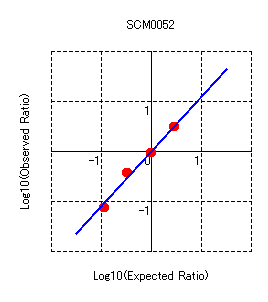

Supplement: Additional file 5 — Evaluation of 5,038 GSPs. A mini-website to browse plots similar to those shown in Additional data file 4 for all the 5,038 GSPs. [file 1471-2164-9-574-S5.zip › image/SCM0052.png]

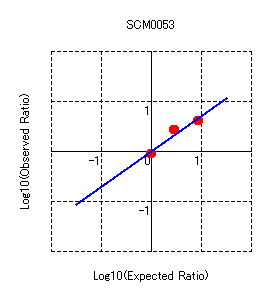

Supplement: Additional file 5 — Evaluation of 5,038 GSPs. A mini-website to browse plots similar to those shown in Additional data file 4 for all the 5,038 GSPs. [file 1471-2164-9-574-S5.zip › image/SCM0053.png]

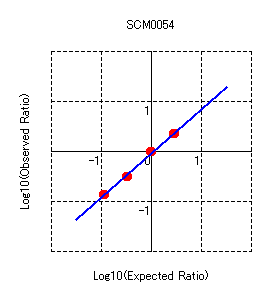

Supplement: Additional file 5 — Evaluation of 5,038 GSPs. A mini-website to browse plots similar to those shown in Additional data file 4 for all the 5,038 GSPs. [file 1471-2164-9-574-S5.zip › image/SCM0054.png]

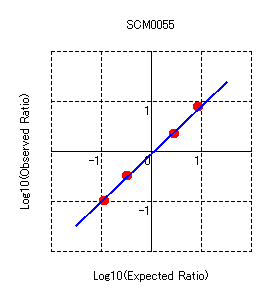

Supplement: Additional file 5 — Evaluation of 5,038 GSPs. A mini-website to browse plots similar to those shown in Additional data file 4 for all the 5,038 GSPs. [file 1471-2164-9-574-S5.zip › image/SCM0055.png]

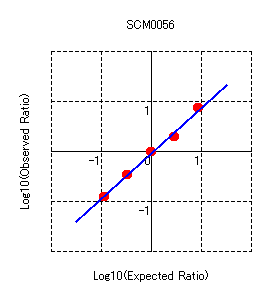

Supplement: Additional file 5 — Evaluation of 5,038 GSPs. A mini-website to browse plots similar to those shown in Additional data file 4 for all the 5,038 GSPs. [file 1471-2164-9-574-S5.zip › image/SCM0056.png]

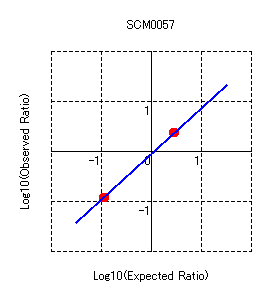

Supplement: Additional file 5 — Evaluation of 5,038 GSPs. A mini-website to browse plots similar to those shown in Additional data file 4 for all the 5,038 GSPs. [file 1471-2164-9-574-S5.zip › image/SCM0057.png]

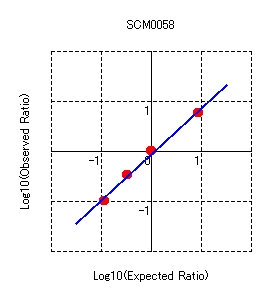

Supplement: Additional file 5 — Evaluation of 5,038 GSPs. A mini-website to browse plots similar to those shown in Additional data file 4 for all the 5,038 GSPs. [file 1471-2164-9-574-S5.zip › image/SCM0058.png]

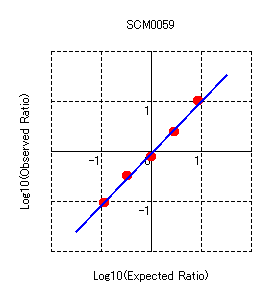

Supplement: Additional file 5 — Evaluation of 5,038 GSPs. A mini-website to browse plots similar to those shown in Additional data file 4 for all the 5,038 GSPs. [file 1471-2164-9-574-S5.zip › image/SCM0059.png]

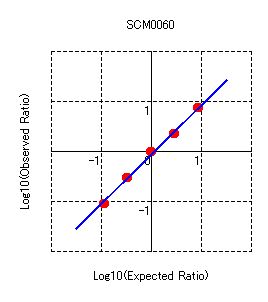

Supplement: Additional file 5 — Evaluation of 5,038 GSPs. A mini-website to browse plots similar to those shown in Additional data file 4 for all the 5,038 GSPs. [file 1471-2164-9-574-S5.zip › image/SCM0060.png]

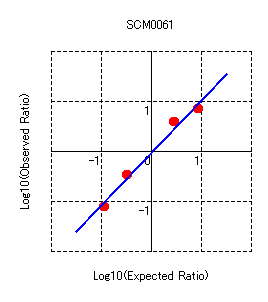

Supplement: Additional file 5 — Evaluation of 5,038 GSPs. A mini-website to browse plots similar to those shown in Additional data file 4 for all the 5,038 GSPs. [file 1471-2164-9-574-S5.zip › image/SCM0061.png]

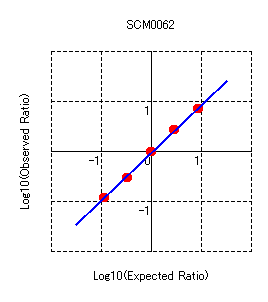

Supplement: Additional file 5 — Evaluation of 5,038 GSPs. A mini-website to browse plots similar to those shown in Additional data file 4 for all the 5,038 GSPs. [file 1471-2164-9-574-S5.zip › image/SCM0062.png]

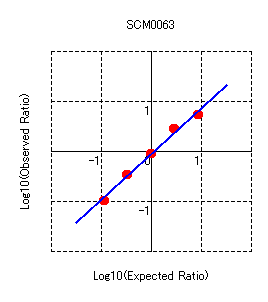

Supplement: Additional file 5 — Evaluation of 5,038 GSPs. A mini-website to browse plots similar to those shown in Additional data file 4 for all the 5,038 GSPs. [file 1471-2164-9-574-S5.zip › image/SCM0063.png]

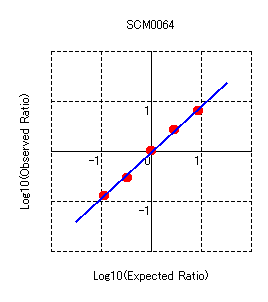

Supplement: Additional file 5 — Evaluation of 5,038 GSPs. A mini-website to browse plots similar to those shown in Additional data file 4 for all the 5,038 GSPs. [file 1471-2164-9-574-S5.zip › image/SCM0064.png]

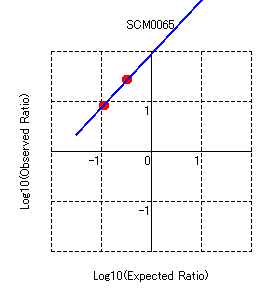

Supplement: Additional file 5 — Evaluation of 5,038 GSPs. A mini-website to browse plots similar to those shown in Additional data file 4 for all the 5,038 GSPs. [file 1471-2164-9-574-S5.zip › image/SCM0065.png]

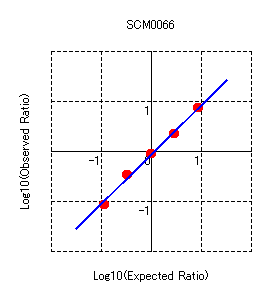

Supplement: Additional file 5 — Evaluation of 5,038 GSPs. A mini-website to browse plots similar to those shown in Additional data file 4 for all the 5,038 GSPs. [file 1471-2164-9-574-S5.zip › image/SCM0066.png]

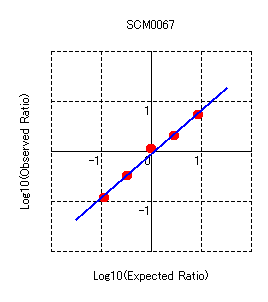

Supplement: Additional file 5 — Evaluation of 5,038 GSPs. A mini-website to browse plots similar to those shown in Additional data file 4 for all the 5,038 GSPs. [file 1471-2164-9-574-S5.zip › image/SCM0067.png]

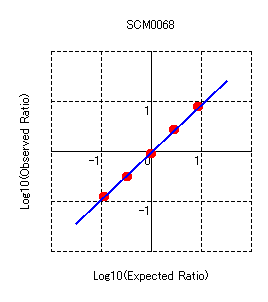

Supplement: Additional file 5 — Evaluation of 5,038 GSPs. A mini-website to browse plots similar to those shown in Additional data file 4 for all the 5,038 GSPs. [file 1471-2164-9-574-S5.zip › image/SCM0068.png]

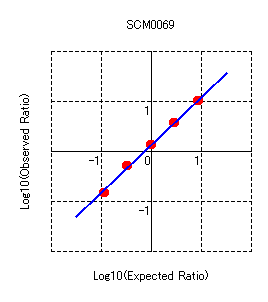

Supplement: Additional file 5 — Evaluation of 5,038 GSPs. A mini-website to browse plots similar to those shown in Additional data file 4 for all the 5,038 GSPs. [file 1471-2164-9-574-S5.zip › image/SCM0069.png]

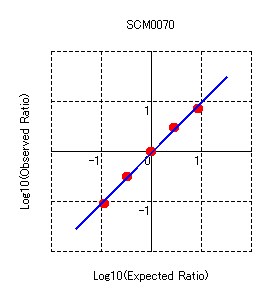

Supplement: Additional file 5 — Evaluation of 5,038 GSPs. A mini-website to browse plots similar to those shown in Additional data file 4 for all the 5,038 GSPs. [file 1471-2164-9-574-S5.zip › image/SCM0070.png]

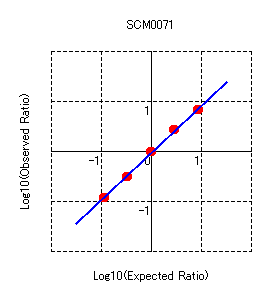

Supplement: Additional file 5 — Evaluation of 5,038 GSPs. A mini-website to browse plots similar to those shown in Additional data file 4 for all the 5,038 GSPs. [file 1471-2164-9-574-S5.zip › image/SCM0071.png]

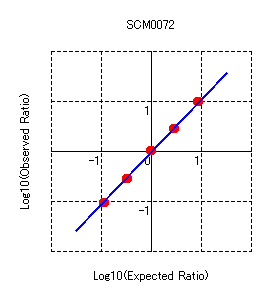

Supplement: Additional file 5 — Evaluation of 5,038 GSPs. A mini-website to browse plots similar to those shown in Additional data file 4 for all the 5,038 GSPs. [file 1471-2164-9-574-S5.zip › image/SCM0072.png]

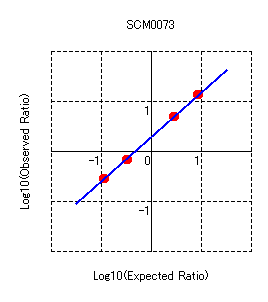

Supplement: Additional file 5 — Evaluation of 5,038 GSPs. A mini-website to browse plots similar to those shown in Additional data file 4 for all the 5,038 GSPs. [file 1471-2164-9-574-S5.zip › image/SCM0073.png]

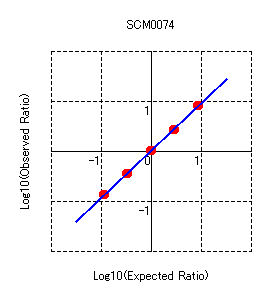

Supplement: Additional file 5 — Evaluation of 5,038 GSPs. A mini-website to browse plots similar to those shown in Additional data file 4 for all the 5,038 GSPs. [file 1471-2164-9-574-S5.zip › image/SCM0074.png]

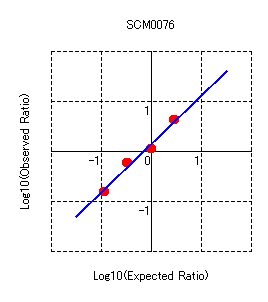

Supplement: Additional file 5 — Evaluation of 5,038 GSPs. A mini-website to browse plots similar to those shown in Additional data file 4 for all the 5,038 GSPs. [file 1471-2164-9-574-S5.zip › image/SCM0076.png]

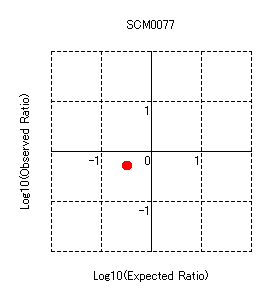

Supplement: Additional file 5 — Evaluation of 5,038 GSPs. A mini-website to browse plots similar to those shown in Additional data file 4 for all the 5,038 GSPs. [file 1471-2164-9-574-S5.zip › image/SCM0077.png]

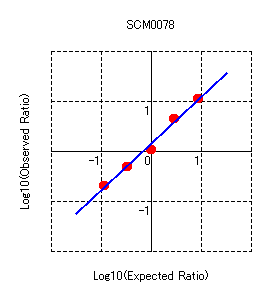

Supplement: Additional file 5 — Evaluation of 5,038 GSPs. A mini-website to browse plots similar to those shown in Additional data file 4 for all the 5,038 GSPs. [file 1471-2164-9-574-S5.zip › image/SCM0078.png]

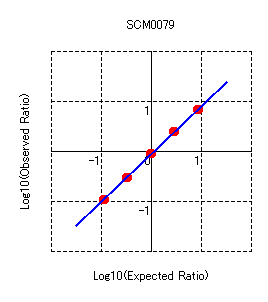

Supplement: Additional file 5 — Evaluation of 5,038 GSPs. A mini-website to browse plots similar to those shown in Additional data file 4 for all the 5,038 GSPs. [file 1471-2164-9-574-S5.zip › image/SCM0079.png]

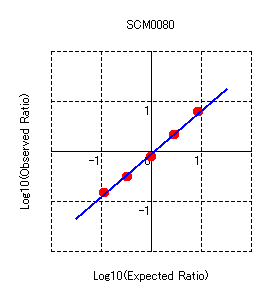

Supplement: Additional file 5 — Evaluation of 5,038 GSPs. A mini-website to browse plots similar to those shown in Additional data file 4 for all the 5,038 GSPs. [file 1471-2164-9-574-S5.zip › image/SCM0080.png]

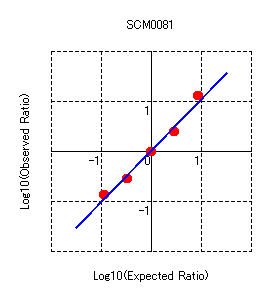

Supplement: Additional file 5 — Evaluation of 5,038 GSPs. A mini-website to browse plots similar to those shown in Additional data file 4 for all the 5,038 GSPs. [file 1471-2164-9-574-S5.zip › image/SCM0081.png]

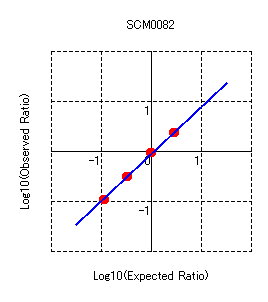

Supplement: Additional file 5 — Evaluation of 5,038 GSPs. A mini-website to browse plots similar to those shown in Additional data file 4 for all the 5,038 GSPs. [file 1471-2164-9-574-S5.zip › image/SCM0082.png]

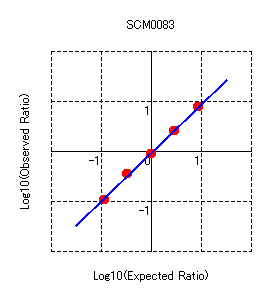

Supplement: Additional file 5 — Evaluation of 5,038 GSPs. A mini-website to browse plots similar to those shown in Additional data file 4 for all the 5,038 GSPs. [file 1471-2164-9-574-S5.zip › image/SCM0083.png]

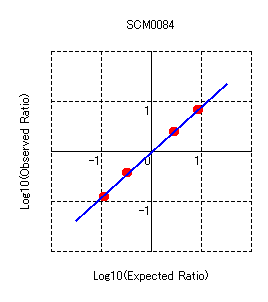

Supplement: Additional file 5 — Evaluation of 5,038 GSPs. A mini-website to browse plots similar to those shown in Additional data file 4 for all the 5,038 GSPs. [file 1471-2164-9-574-S5.zip › image/SCM0084.png]

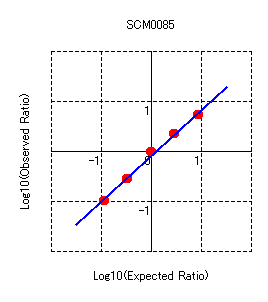

Supplement: Additional file 5 — Evaluation of 5,038 GSPs. A mini-website to browse plots similar to those shown in Additional data file 4 for all the 5,038 GSPs. [file 1471-2164-9-574-S5.zip › image/SCM0085.png]

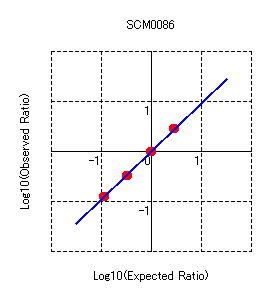

Supplement: Additional file 5 — Evaluation of 5,038 GSPs. A mini-website to browse plots similar to those shown in Additional data file 4 for all the 5,038 GSPs. [file 1471-2164-9-574-S5.zip › image/SCM0086.png]

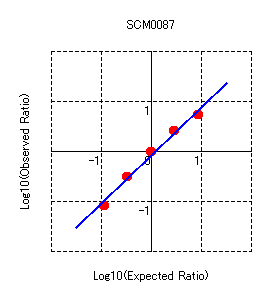

Supplement: Additional file 5 — Evaluation of 5,038 GSPs. A mini-website to browse plots similar to those shown in Additional data file 4 for all the 5,038 GSPs. [file 1471-2164-9-574-S5.zip › image/SCM0087.png]

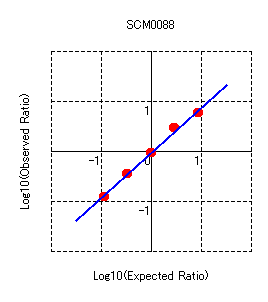

Supplement: Additional file 5 — Evaluation of 5,038 GSPs. A mini-website to browse plots similar to those shown in Additional data file 4 for all the 5,038 GSPs. [file 1471-2164-9-574-S5.zip › image/SCM0088.png]

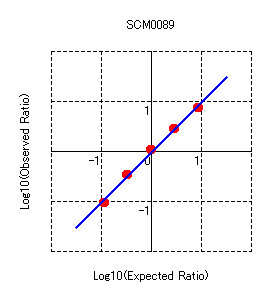

Supplement: Additional file 5 — Evaluation of 5,038 GSPs. A mini-website to browse plots similar to those shown in Additional data file 4 for all the 5,038 GSPs. [file 1471-2164-9-574-S5.zip › image/SCM0089.png]

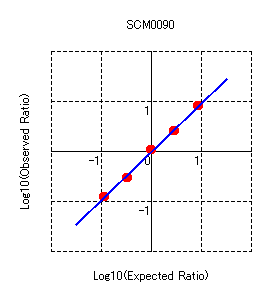

Supplement: Additional file 5 — Evaluation of 5,038 GSPs. A mini-website to browse plots similar to those shown in Additional data file 4 for all the 5,038 GSPs. [file 1471-2164-9-574-S5.zip › image/SCM0090.png]

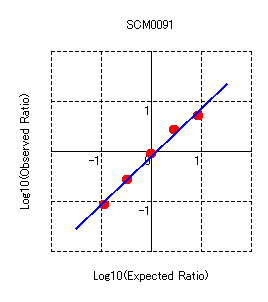

Supplement: Additional file 5 — Evaluation of 5,038 GSPs. A mini-website to browse plots similar to those shown in Additional data file 4 for all the 5,038 GSPs. [file 1471-2164-9-574-S5.zip › image/SCM0091.png]

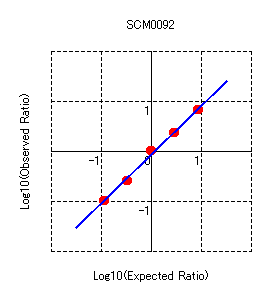

Supplement: Additional file 5 — Evaluation of 5,038 GSPs. A mini-website to browse plots similar to those shown in Additional data file 4 for all the 5,038 GSPs. [file 1471-2164-9-574-S5.zip › image/SCM0092.png]

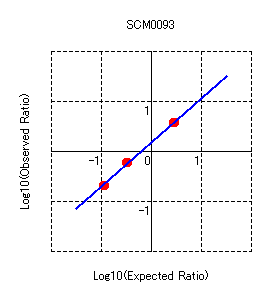

Supplement: Additional file 5 — Evaluation of 5,038 GSPs. A mini-website to browse plots similar to those shown in Additional data file 4 for all the 5,038 GSPs. [file 1471-2164-9-574-S5.zip › image/SCM0093.png]

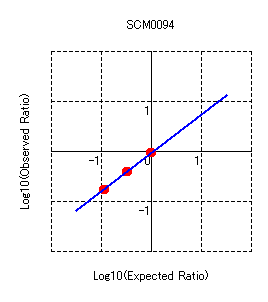

Supplement: Additional file 5 — Evaluation of 5,038 GSPs. A mini-website to browse plots similar to those shown in Additional data file 4 for all the 5,038 GSPs. [file 1471-2164-9-574-S5.zip › image/SCM0094.png]

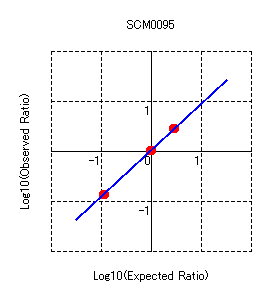

Supplement: Additional file 5 — Evaluation of 5,038 GSPs. A mini-website to browse plots similar to those shown in Additional data file 4 for all the 5,038 GSPs. [file 1471-2164-9-574-S5.zip › image/SCM0095.png]

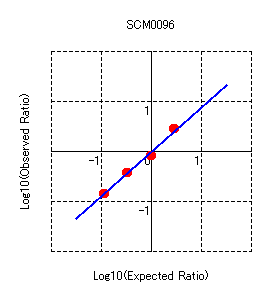

Supplement: Additional file 5 — Evaluation of 5,038 GSPs. A mini-website to browse plots similar to those shown in Additional data file 4 for all the 5,038 GSPs. [file 1471-2164-9-574-S5.zip › image/SCM0096.png]

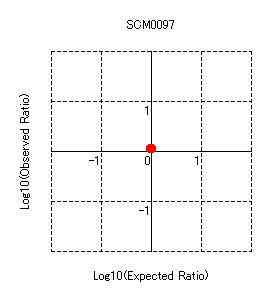

Supplement: Additional file 5 — Evaluation of 5,038 GSPs. A mini-website to browse plots similar to those shown in Additional data file 4 for all the 5,038 GSPs. [file 1471-2164-9-574-S5.zip › image/SCM0097.png]

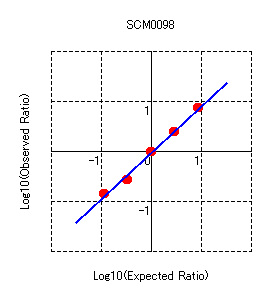

Supplement: Additional file 5 — Evaluation of 5,038 GSPs. A mini-website to browse plots similar to those shown in Additional data file 4 for all the 5,038 GSPs. [file 1471-2164-9-574-S5.zip › image/SCM0098.png]
