# Supplementary figures and images for: Absolute quantification of the budding yeast transcriptome by means of competitive PCR between genomic and complementary DNAs (part 3 of 3)
Source: BMC Genomics. 2008 Nov 29;9:574. doi: 10.1186/1471-2164-9-574 (PMC2612024; doi:10.1186/1471-2164-9-574)

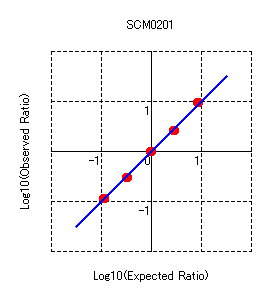

Supplement: Additional file 5 — Evaluation of 5,038 GSPs. A mini-website to browse plots similar to those shown in Additional data file 4 for all the 5,038 GSPs. [file 1471-2164-9-574-S5.zip › image/SCM0201.png]

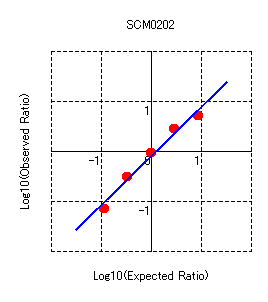

Supplement: Additional file 5 — Evaluation of 5,038 GSPs. A mini-website to browse plots similar to those shown in Additional data file 4 for all the 5,038 GSPs. [file 1471-2164-9-574-S5.zip › image/SCM0202.png]

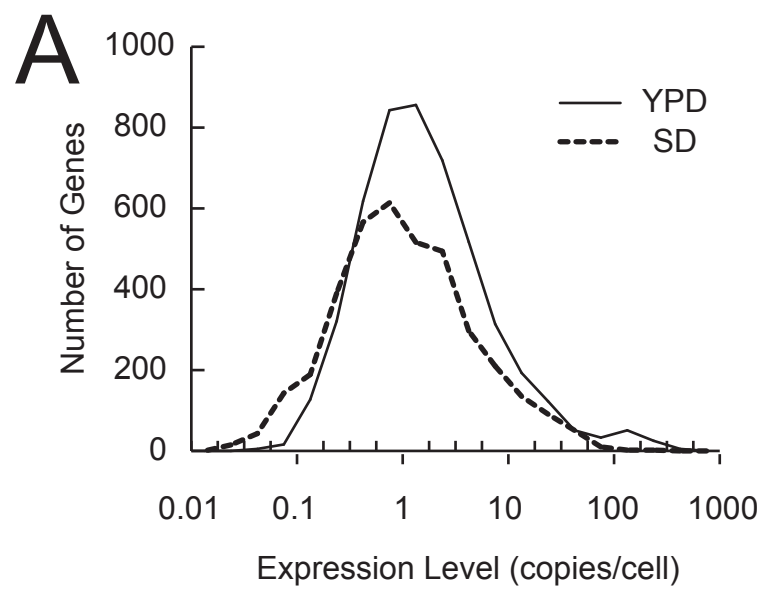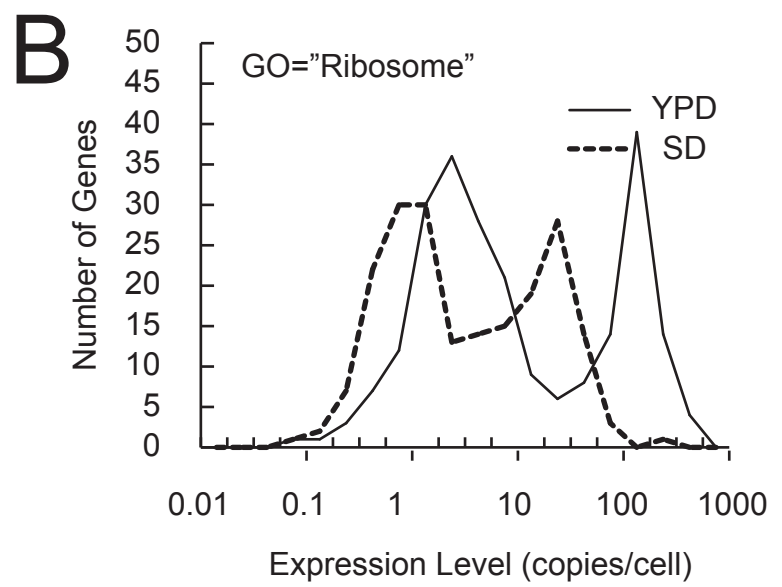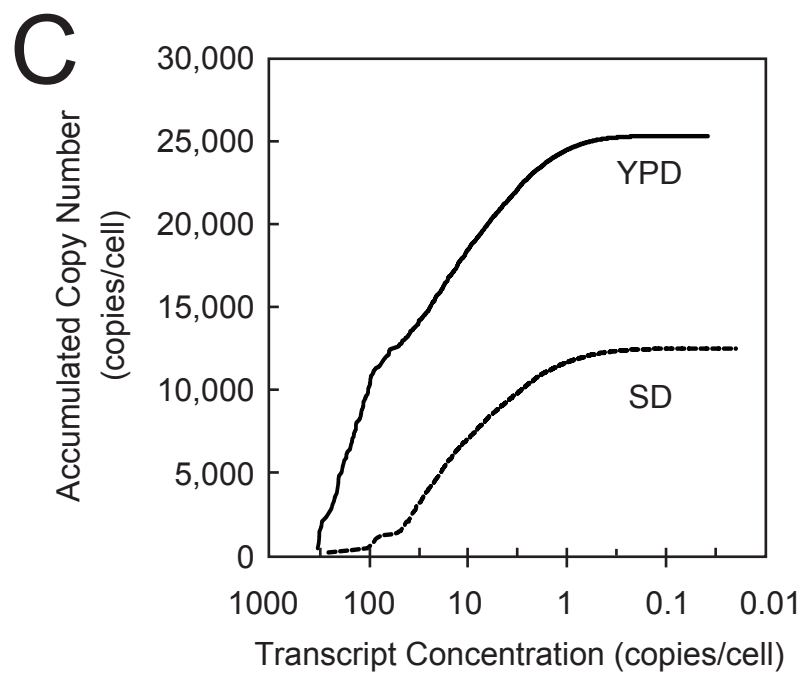

Supplement: Additional file 7 — Comparison of transcriptome between cells grown in YPD and SD media. (A) Distribution of transcript abundances in cells grown in YPD and SD media. The plot is similar to that in Figure 3C but contains every gene quantified in each condition. (B) Distribution of transcript abundances for genes to which GO slim term "Ribosome" is assigned. Data are shown for both cells grown in YPD and SD media. The plot includes every gene in the category successfully quantified in each condition. (C) "Virtual R0t" curve for 3,351 genes detectably expressed in cells grown in YPD and SD media. [file 1471-2164-9-574-S7.pdf]
