# Supplementary figures and images for: Absolute quantification of the budding yeast transcriptome by means of competitive PCR between genomic and complementary DNAs (part 2 of 3)
Source: BMC Genomics. 2008 Nov 29;9:574. doi: 10.1186/1471-2164-9-574 (PMC2612024; doi:10.1186/1471-2164-9-574)

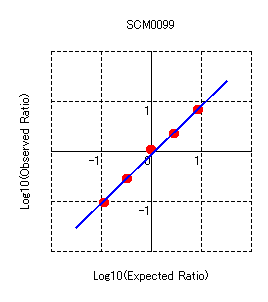

Supplement: Additional file 5 — Evaluation of 5,038 GSPs. A mini-website to browse plots similar to those shown in Additional data file 4 for all the 5,038 GSPs. [file 1471-2164-9-574-S5.zip › image/SCM0099.png]

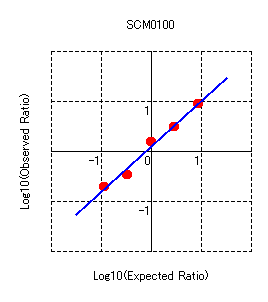

Supplement: Additional file 5 — Evaluation of 5,038 GSPs. A mini-website to browse plots similar to those shown in Additional data file 4 for all the 5,038 GSPs. [file 1471-2164-9-574-S5.zip › image/SCM0100.png]

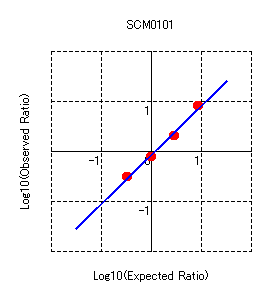

Supplement: Additional file 5 — Evaluation of 5,038 GSPs. A mini-website to browse plots similar to those shown in Additional data file 4 for all the 5,038 GSPs. [file 1471-2164-9-574-S5.zip › image/SCM0101.png]

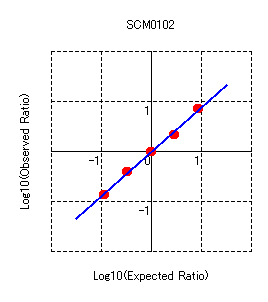

Supplement: Additional file 5 — Evaluation of 5,038 GSPs. A mini-website to browse plots similar to those shown in Additional data file 4 for all the 5,038 GSPs. [file 1471-2164-9-574-S5.zip › image/SCM0102.png]

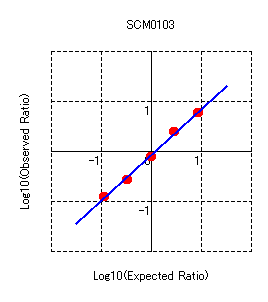

Supplement: Additional file 5 — Evaluation of 5,038 GSPs. A mini-website to browse plots similar to those shown in Additional data file 4 for all the 5,038 GSPs. [file 1471-2164-9-574-S5.zip › image/SCM0103.png]

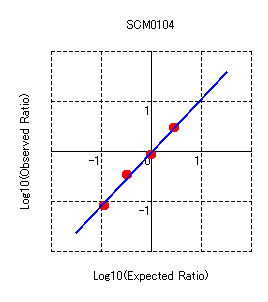

Supplement: Additional file 5 — Evaluation of 5,038 GSPs. A mini-website to browse plots similar to those shown in Additional data file 4 for all the 5,038 GSPs. [file 1471-2164-9-574-S5.zip › image/SCM0104.png]

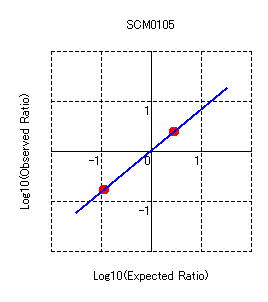

Supplement: Additional file 5 — Evaluation of 5,038 GSPs. A mini-website to browse plots similar to those shown in Additional data file 4 for all the 5,038 GSPs. [file 1471-2164-9-574-S5.zip › image/SCM0105.png]

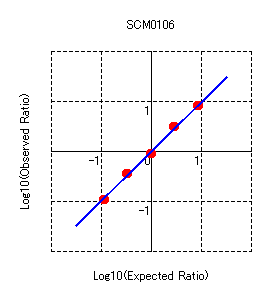

Supplement: Additional file 5 — Evaluation of 5,038 GSPs. A mini-website to browse plots similar to those shown in Additional data file 4 for all the 5,038 GSPs. [file 1471-2164-9-574-S5.zip › image/SCM0106.png]

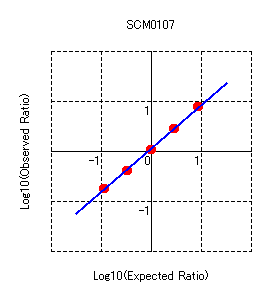

Supplement: Additional file 5 — Evaluation of 5,038 GSPs. A mini-website to browse plots similar to those shown in Additional data file 4 for all the 5,038 GSPs. [file 1471-2164-9-574-S5.zip › image/SCM0107.png]

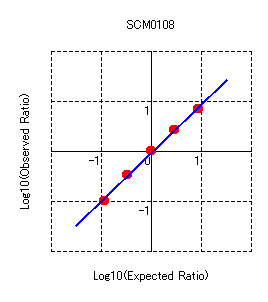

Supplement: Additional file 5 — Evaluation of 5,038 GSPs. A mini-website to browse plots similar to those shown in Additional data file 4 for all the 5,038 GSPs. [file 1471-2164-9-574-S5.zip › image/SCM0108.png]

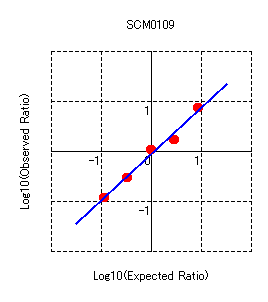

Supplement: Additional file 5 — Evaluation of 5,038 GSPs. A mini-website to browse plots similar to those shown in Additional data file 4 for all the 5,038 GSPs. [file 1471-2164-9-574-S5.zip › image/SCM0109.png]

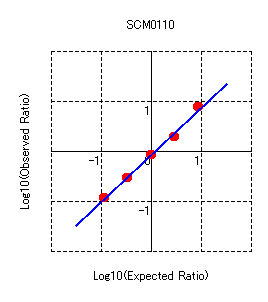

Supplement: Additional file 5 — Evaluation of 5,038 GSPs. A mini-website to browse plots similar to those shown in Additional data file 4 for all the 5,038 GSPs. [file 1471-2164-9-574-S5.zip › image/SCM0110.png]

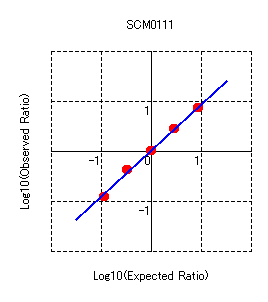

Supplement: Additional file 5 — Evaluation of 5,038 GSPs. A mini-website to browse plots similar to those shown in Additional data file 4 for all the 5,038 GSPs. [file 1471-2164-9-574-S5.zip › image/SCM0111.png]

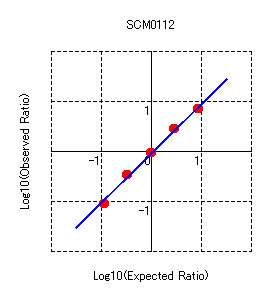

Supplement: Additional file 5 — Evaluation of 5,038 GSPs. A mini-website to browse plots similar to those shown in Additional data file 4 for all the 5,038 GSPs. [file 1471-2164-9-574-S5.zip › image/SCM0112.png]

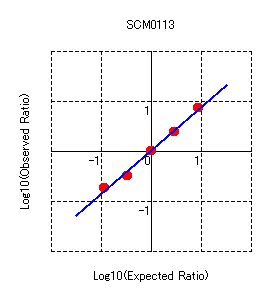

Supplement: Additional file 5 — Evaluation of 5,038 GSPs. A mini-website to browse plots similar to those shown in Additional data file 4 for all the 5,038 GSPs. [file 1471-2164-9-574-S5.zip › image/SCM0113.png]

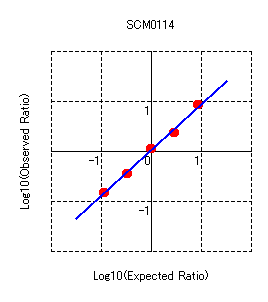

Supplement: Additional file 5 — Evaluation of 5,038 GSPs. A mini-website to browse plots similar to those shown in Additional data file 4 for all the 5,038 GSPs. [file 1471-2164-9-574-S5.zip › image/SCM0114.png]

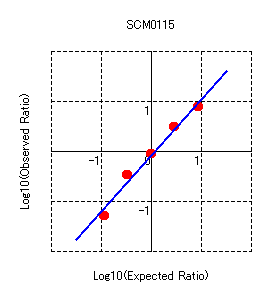

Supplement: Additional file 5 — Evaluation of 5,038 GSPs. A mini-website to browse plots similar to those shown in Additional data file 4 for all the 5,038 GSPs. [file 1471-2164-9-574-S5.zip › image/SCM0115.png]

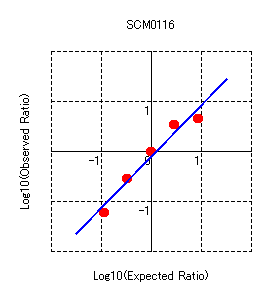

Supplement: Additional file 5 — Evaluation of 5,038 GSPs. A mini-website to browse plots similar to those shown in Additional data file 4 for all the 5,038 GSPs. [file 1471-2164-9-574-S5.zip › image/SCM0116.png]

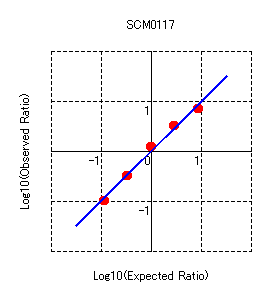

Supplement: Additional file 5 — Evaluation of 5,038 GSPs. A mini-website to browse plots similar to those shown in Additional data file 4 for all the 5,038 GSPs. [file 1471-2164-9-574-S5.zip › image/SCM0117.png]

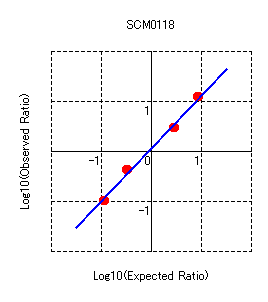

Supplement: Additional file 5 — Evaluation of 5,038 GSPs. A mini-website to browse plots similar to those shown in Additional data file 4 for all the 5,038 GSPs. [file 1471-2164-9-574-S5.zip › image/SCM0118.png]

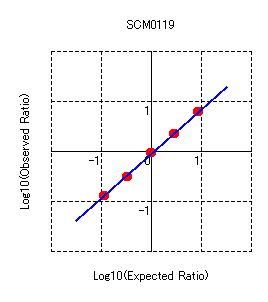

Supplement: Additional file 5 — Evaluation of 5,038 GSPs. A mini-website to browse plots similar to those shown in Additional data file 4 for all the 5,038 GSPs. [file 1471-2164-9-574-S5.zip › image/SCM0119.png]

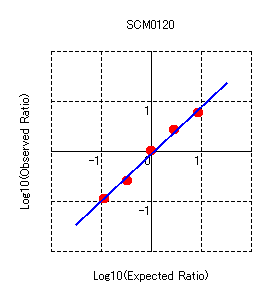

Supplement: Additional file 5 — Evaluation of 5,038 GSPs. A mini-website to browse plots similar to those shown in Additional data file 4 for all the 5,038 GSPs. [file 1471-2164-9-574-S5.zip › image/SCM0120.png]

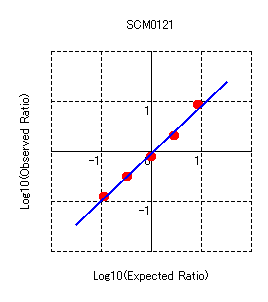

Supplement: Additional file 5 — Evaluation of 5,038 GSPs. A mini-website to browse plots similar to those shown in Additional data file 4 for all the 5,038 GSPs. [file 1471-2164-9-574-S5.zip › image/SCM0121.png]

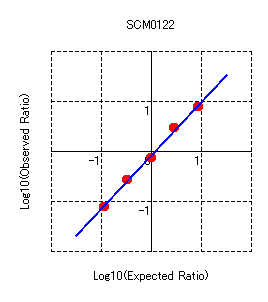

Supplement: Additional file 5 — Evaluation of 5,038 GSPs. A mini-website to browse plots similar to those shown in Additional data file 4 for all the 5,038 GSPs. [file 1471-2164-9-574-S5.zip › image/SCM0122.png]

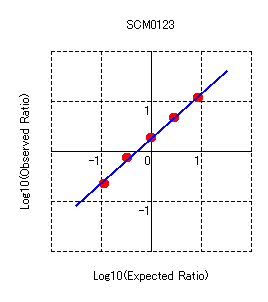

Supplement: Additional file 5 — Evaluation of 5,038 GSPs. A mini-website to browse plots similar to those shown in Additional data file 4 for all the 5,038 GSPs. [file 1471-2164-9-574-S5.zip › image/SCM0123.png]

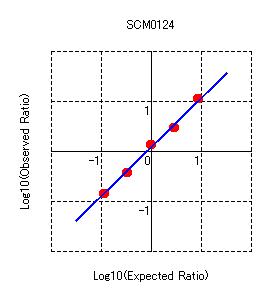

Supplement: Additional file 5 — Evaluation of 5,038 GSPs. A mini-website to browse plots similar to those shown in Additional data file 4 for all the 5,038 GSPs. [file 1471-2164-9-574-S5.zip › image/SCM0124.png]

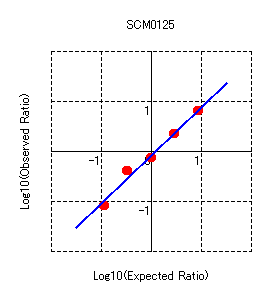

Supplement: Additional file 5 — Evaluation of 5,038 GSPs. A mini-website to browse plots similar to those shown in Additional data file 4 for all the 5,038 GSPs. [file 1471-2164-9-574-S5.zip › image/SCM0125.png]

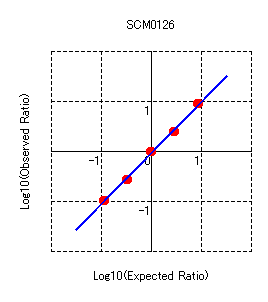

Supplement: Additional file 5 — Evaluation of 5,038 GSPs. A mini-website to browse plots similar to those shown in Additional data file 4 for all the 5,038 GSPs. [file 1471-2164-9-574-S5.zip › image/SCM0126.png]

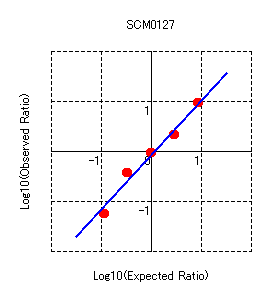

Supplement: Additional file 5 — Evaluation of 5,038 GSPs. A mini-website to browse plots similar to those shown in Additional data file 4 for all the 5,038 GSPs. [file 1471-2164-9-574-S5.zip › image/SCM0127.png]

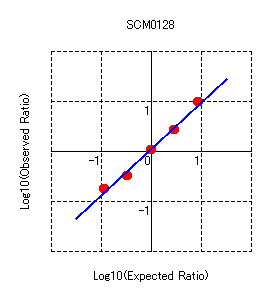

Supplement: Additional file 5 — Evaluation of 5,038 GSPs. A mini-website to browse plots similar to those shown in Additional data file 4 for all the 5,038 GSPs. [file 1471-2164-9-574-S5.zip › image/SCM0128.png]

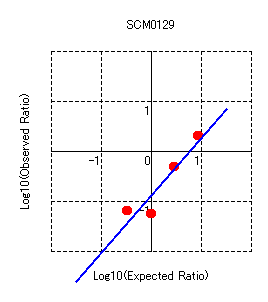

Supplement: Additional file 5 — Evaluation of 5,038 GSPs. A mini-website to browse plots similar to those shown in Additional data file 4 for all the 5,038 GSPs. [file 1471-2164-9-574-S5.zip › image/SCM0129.png]

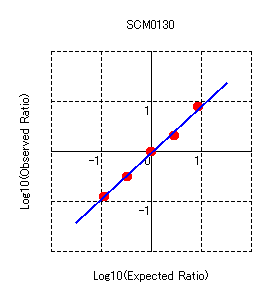

Supplement: Additional file 5 — Evaluation of 5,038 GSPs. A mini-website to browse plots similar to those shown in Additional data file 4 for all the 5,038 GSPs. [file 1471-2164-9-574-S5.zip › image/SCM0130.png]

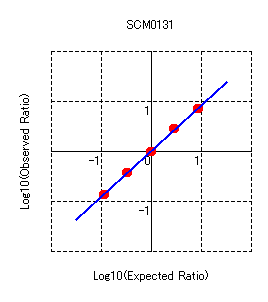

Supplement: Additional file 5 — Evaluation of 5,038 GSPs. A mini-website to browse plots similar to those shown in Additional data file 4 for all the 5,038 GSPs. [file 1471-2164-9-574-S5.zip › image/SCM0131.png]

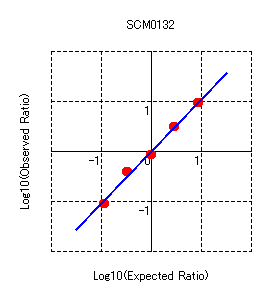

Supplement: Additional file 5 — Evaluation of 5,038 GSPs. A mini-website to browse plots similar to those shown in Additional data file 4 for all the 5,038 GSPs. [file 1471-2164-9-574-S5.zip › image/SCM0132.png]

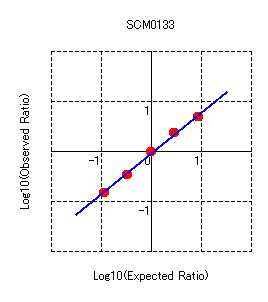

Supplement: Additional file 5 — Evaluation of 5,038 GSPs. A mini-website to browse plots similar to those shown in Additional data file 4 for all the 5,038 GSPs. [file 1471-2164-9-574-S5.zip › image/SCM0133.png]

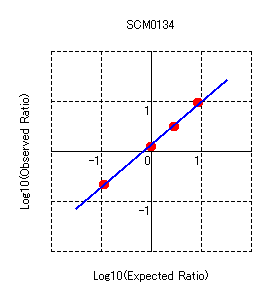

Supplement: Additional file 5 — Evaluation of 5,038 GSPs. A mini-website to browse plots similar to those shown in Additional data file 4 for all the 5,038 GSPs. [file 1471-2164-9-574-S5.zip › image/SCM0134.png]

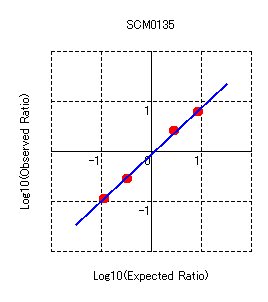

Supplement: Additional file 5 — Evaluation of 5,038 GSPs. A mini-website to browse plots similar to those shown in Additional data file 4 for all the 5,038 GSPs. [file 1471-2164-9-574-S5.zip › image/SCM0135.png]

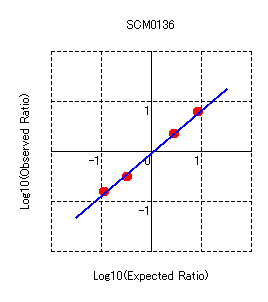

Supplement: Additional file 5 — Evaluation of 5,038 GSPs. A mini-website to browse plots similar to those shown in Additional data file 4 for all the 5,038 GSPs. [file 1471-2164-9-574-S5.zip › image/SCM0136.png]

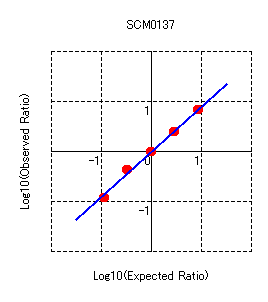

Supplement: Additional file 5 — Evaluation of 5,038 GSPs. A mini-website to browse plots similar to those shown in Additional data file 4 for all the 5,038 GSPs. [file 1471-2164-9-574-S5.zip › image/SCM0137.png]

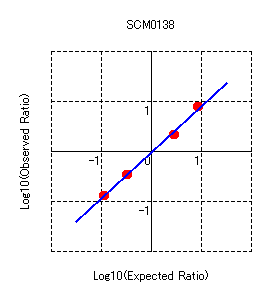

Supplement: Additional file 5 — Evaluation of 5,038 GSPs. A mini-website to browse plots similar to those shown in Additional data file 4 for all the 5,038 GSPs. [file 1471-2164-9-574-S5.zip › image/SCM0138.png]

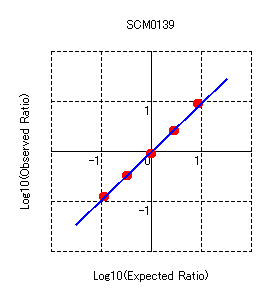

Supplement: Additional file 5 — Evaluation of 5,038 GSPs. A mini-website to browse plots similar to those shown in Additional data file 4 for all the 5,038 GSPs. [file 1471-2164-9-574-S5.zip › image/SCM0139.png]

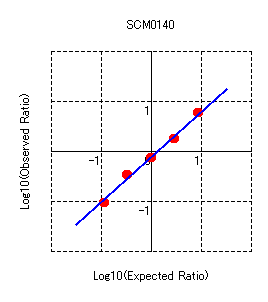

Supplement: Additional file 5 — Evaluation of 5,038 GSPs. A mini-website to browse plots similar to those shown in Additional data file 4 for all the 5,038 GSPs. [file 1471-2164-9-574-S5.zip › image/SCM0140.png]

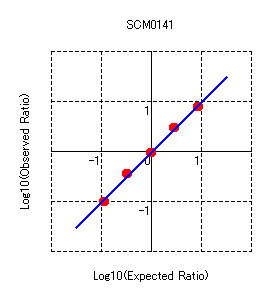

Supplement: Additional file 5 — Evaluation of 5,038 GSPs. A mini-website to browse plots similar to those shown in Additional data file 4 for all the 5,038 GSPs. [file 1471-2164-9-574-S5.zip › image/SCM0141.png]

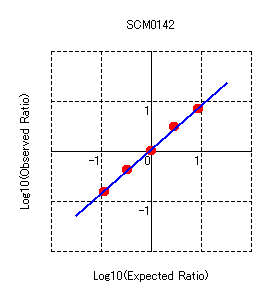

Supplement: Additional file 5 — Evaluation of 5,038 GSPs. A mini-website to browse plots similar to those shown in Additional data file 4 for all the 5,038 GSPs. [file 1471-2164-9-574-S5.zip › image/SCM0142.png]

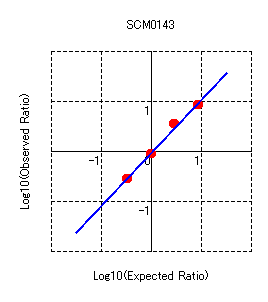

Supplement: Additional file 5 — Evaluation of 5,038 GSPs. A mini-website to browse plots similar to those shown in Additional data file 4 for all the 5,038 GSPs. [file 1471-2164-9-574-S5.zip › image/SCM0143.png]

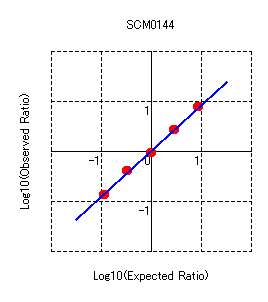

Supplement: Additional file 5 — Evaluation of 5,038 GSPs. A mini-website to browse plots similar to those shown in Additional data file 4 for all the 5,038 GSPs. [file 1471-2164-9-574-S5.zip › image/SCM0144.png]

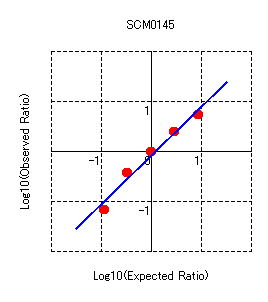

Supplement: Additional file 5 — Evaluation of 5,038 GSPs. A mini-website to browse plots similar to those shown in Additional data file 4 for all the 5,038 GSPs. [file 1471-2164-9-574-S5.zip › image/SCM0145.png]

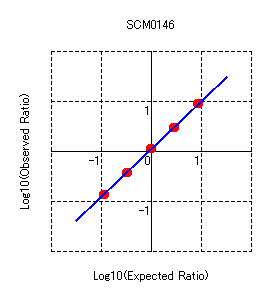

Supplement: Additional file 5 — Evaluation of 5,038 GSPs. A mini-website to browse plots similar to those shown in Additional data file 4 for all the 5,038 GSPs. [file 1471-2164-9-574-S5.zip › image/SCM0146.png]

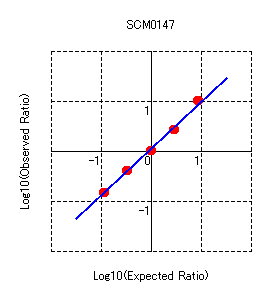

Supplement: Additional file 5 — Evaluation of 5,038 GSPs. A mini-website to browse plots similar to those shown in Additional data file 4 for all the 5,038 GSPs. [file 1471-2164-9-574-S5.zip › image/SCM0147.png]

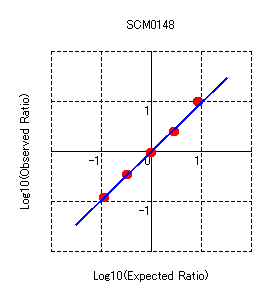

Supplement: Additional file 5 — Evaluation of 5,038 GSPs. A mini-website to browse plots similar to those shown in Additional data file 4 for all the 5,038 GSPs. [file 1471-2164-9-574-S5.zip › image/SCM0148.png]

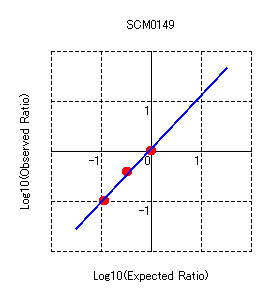

Supplement: Additional file 5 — Evaluation of 5,038 GSPs. A mini-website to browse plots similar to those shown in Additional data file 4 for all the 5,038 GSPs. [file 1471-2164-9-574-S5.zip › image/SCM0149.png]

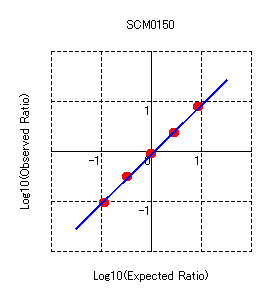

Supplement: Additional file 5 — Evaluation of 5,038 GSPs. A mini-website to browse plots similar to those shown in Additional data file 4 for all the 5,038 GSPs. [file 1471-2164-9-574-S5.zip › image/SCM0150.png]

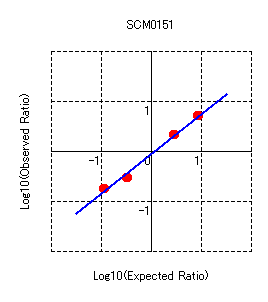

Supplement: Additional file 5 — Evaluation of 5,038 GSPs. A mini-website to browse plots similar to those shown in Additional data file 4 for all the 5,038 GSPs. [file 1471-2164-9-574-S5.zip › image/SCM0151.png]

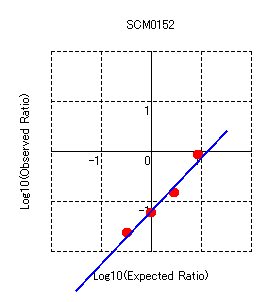

Supplement: Additional file 5 — Evaluation of 5,038 GSPs. A mini-website to browse plots similar to those shown in Additional data file 4 for all the 5,038 GSPs. [file 1471-2164-9-574-S5.zip › image/SCM0152.png]

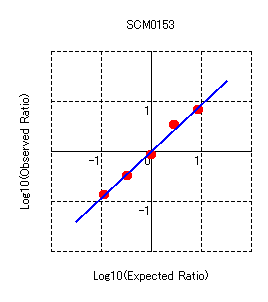

Supplement: Additional file 5 — Evaluation of 5,038 GSPs. A mini-website to browse plots similar to those shown in Additional data file 4 for all the 5,038 GSPs. [file 1471-2164-9-574-S5.zip › image/SCM0153.png]

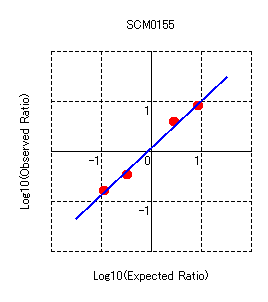

Supplement: Additional file 5 — Evaluation of 5,038 GSPs. A mini-website to browse plots similar to those shown in Additional data file 4 for all the 5,038 GSPs. [file 1471-2164-9-574-S5.zip › image/SCM0155.png]

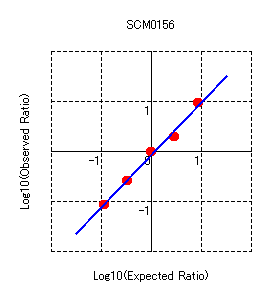

Supplement: Additional file 5 — Evaluation of 5,038 GSPs. A mini-website to browse plots similar to those shown in Additional data file 4 for all the 5,038 GSPs. [file 1471-2164-9-574-S5.zip › image/SCM0156.png]

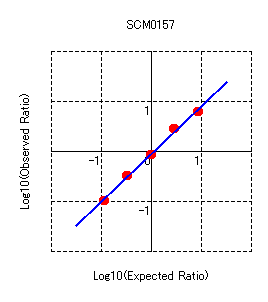

Supplement: Additional file 5 — Evaluation of 5,038 GSPs. A mini-website to browse plots similar to those shown in Additional data file 4 for all the 5,038 GSPs. [file 1471-2164-9-574-S5.zip › image/SCM0157.png]

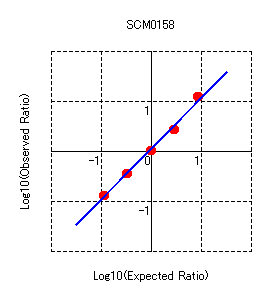

Supplement: Additional file 5 — Evaluation of 5,038 GSPs. A mini-website to browse plots similar to those shown in Additional data file 4 for all the 5,038 GSPs. [file 1471-2164-9-574-S5.zip › image/SCM0158.png]

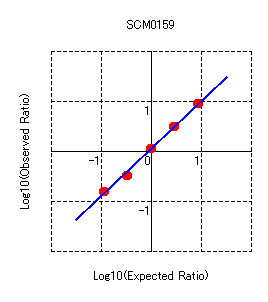

Supplement: Additional file 5 — Evaluation of 5,038 GSPs. A mini-website to browse plots similar to those shown in Additional data file 4 for all the 5,038 GSPs. [file 1471-2164-9-574-S5.zip › image/SCM0159.png]

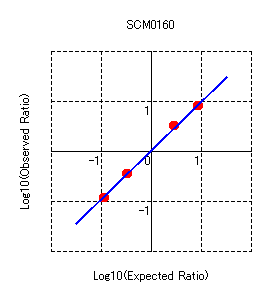

Supplement: Additional file 5 — Evaluation of 5,038 GSPs. A mini-website to browse plots similar to those shown in Additional data file 4 for all the 5,038 GSPs. [file 1471-2164-9-574-S5.zip › image/SCM0160.png]

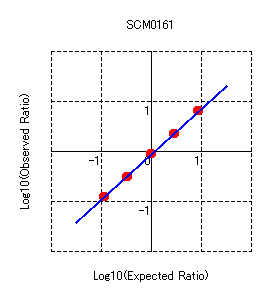

Supplement: Additional file 5 — Evaluation of 5,038 GSPs. A mini-website to browse plots similar to those shown in Additional data file 4 for all the 5,038 GSPs. [file 1471-2164-9-574-S5.zip › image/SCM0161.png]

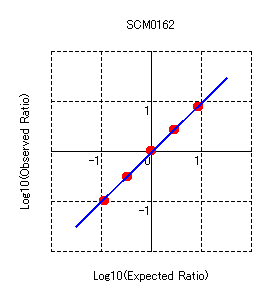

Supplement: Additional file 5 — Evaluation of 5,038 GSPs. A mini-website to browse plots similar to those shown in Additional data file 4 for all the 5,038 GSPs. [file 1471-2164-9-574-S5.zip › image/SCM0162.png]

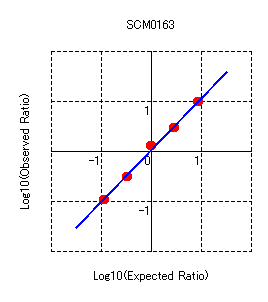

Supplement: Additional file 5 — Evaluation of 5,038 GSPs. A mini-website to browse plots similar to those shown in Additional data file 4 for all the 5,038 GSPs. [file 1471-2164-9-574-S5.zip › image/SCM0163.png]

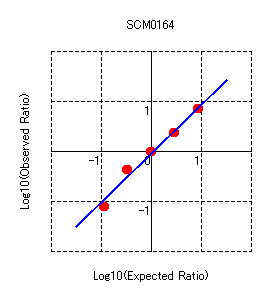

Supplement: Additional file 5 — Evaluation of 5,038 GSPs. A mini-website to browse plots similar to those shown in Additional data file 4 for all the 5,038 GSPs. [file 1471-2164-9-574-S5.zip › image/SCM0164.png]

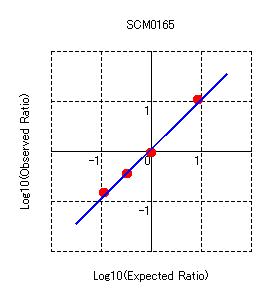

Supplement: Additional file 5 — Evaluation of 5,038 GSPs. A mini-website to browse plots similar to those shown in Additional data file 4 for all the 5,038 GSPs. [file 1471-2164-9-574-S5.zip › image/SCM0165.png]

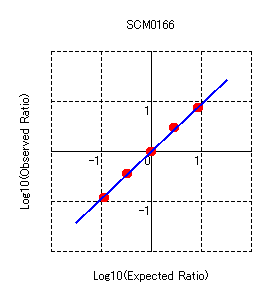

Supplement: Additional file 5 — Evaluation of 5,038 GSPs. A mini-website to browse plots similar to those shown in Additional data file 4 for all the 5,038 GSPs. [file 1471-2164-9-574-S5.zip › image/SCM0166.png]

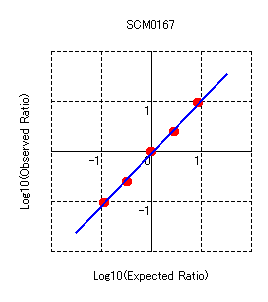

Supplement: Additional file 5 — Evaluation of 5,038 GSPs. A mini-website to browse plots similar to those shown in Additional data file 4 for all the 5,038 GSPs. [file 1471-2164-9-574-S5.zip › image/SCM0167.png]

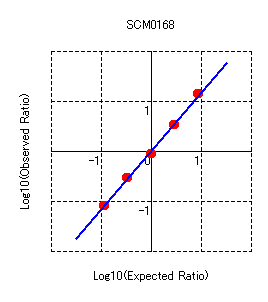

Supplement: Additional file 5 — Evaluation of 5,038 GSPs. A mini-website to browse plots similar to those shown in Additional data file 4 for all the 5,038 GSPs. [file 1471-2164-9-574-S5.zip › image/SCM0168.png]

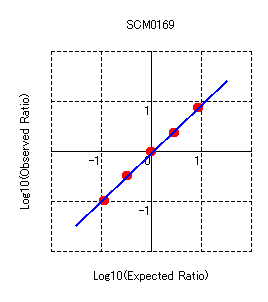

Supplement: Additional file 5 — Evaluation of 5,038 GSPs. A mini-website to browse plots similar to those shown in Additional data file 4 for all the 5,038 GSPs. [file 1471-2164-9-574-S5.zip › image/SCM0169.png]

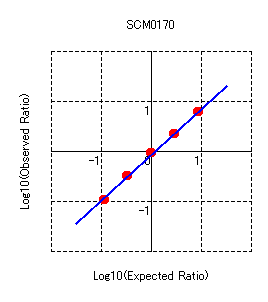

Supplement: Additional file 5 — Evaluation of 5,038 GSPs. A mini-website to browse plots similar to those shown in Additional data file 4 for all the 5,038 GSPs. [file 1471-2164-9-574-S5.zip › image/SCM0170.png]

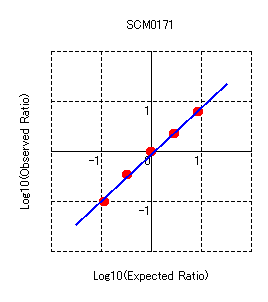

Supplement: Additional file 5 — Evaluation of 5,038 GSPs. A mini-website to browse plots similar to those shown in Additional data file 4 for all the 5,038 GSPs. [file 1471-2164-9-574-S5.zip › image/SCM0171.png]

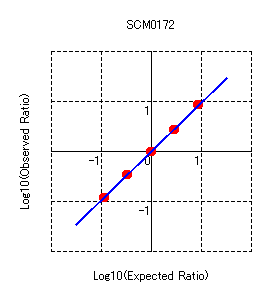

Supplement: Additional file 5 — Evaluation of 5,038 GSPs. A mini-website to browse plots similar to those shown in Additional data file 4 for all the 5,038 GSPs. [file 1471-2164-9-574-S5.zip › image/SCM0172.png]

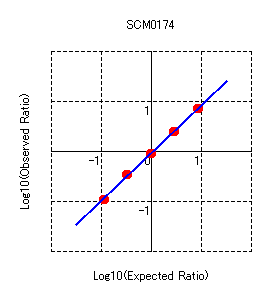

Supplement: Additional file 5 — Evaluation of 5,038 GSPs. A mini-website to browse plots similar to those shown in Additional data file 4 for all the 5,038 GSPs. [file 1471-2164-9-574-S5.zip › image/SCM0174.png]

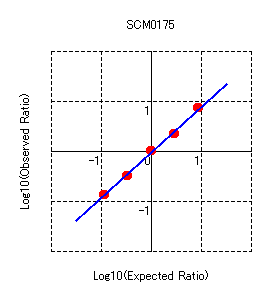

Supplement: Additional file 5 — Evaluation of 5,038 GSPs. A mini-website to browse plots similar to those shown in Additional data file 4 for all the 5,038 GSPs. [file 1471-2164-9-574-S5.zip › image/SCM0175.png]

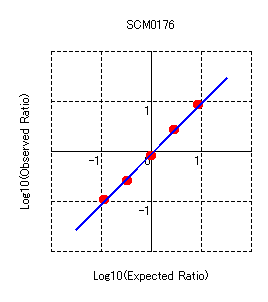

Supplement: Additional file 5 — Evaluation of 5,038 GSPs. A mini-website to browse plots similar to those shown in Additional data file 4 for all the 5,038 GSPs. [file 1471-2164-9-574-S5.zip › image/SCM0176.png]

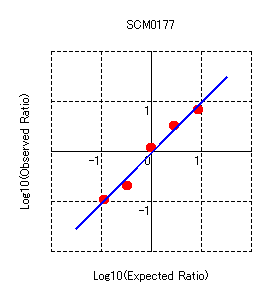

Supplement: Additional file 5 — Evaluation of 5,038 GSPs. A mini-website to browse plots similar to those shown in Additional data file 4 for all the 5,038 GSPs. [file 1471-2164-9-574-S5.zip › image/SCM0177.png]

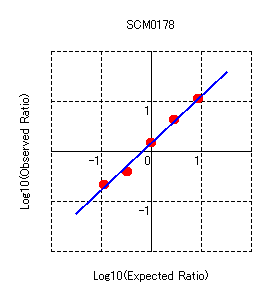

Supplement: Additional file 5 — Evaluation of 5,038 GSPs. A mini-website to browse plots similar to those shown in Additional data file 4 for all the 5,038 GSPs. [file 1471-2164-9-574-S5.zip › image/SCM0178.png]

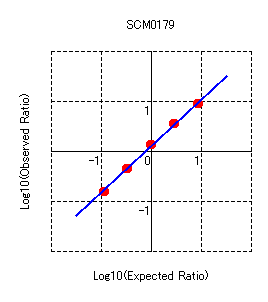

Supplement: Additional file 5 — Evaluation of 5,038 GSPs. A mini-website to browse plots similar to those shown in Additional data file 4 for all the 5,038 GSPs. [file 1471-2164-9-574-S5.zip › image/SCM0179.png]

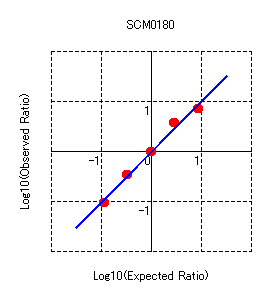

Supplement: Additional file 5 — Evaluation of 5,038 GSPs. A mini-website to browse plots similar to those shown in Additional data file 4 for all the 5,038 GSPs. [file 1471-2164-9-574-S5.zip › image/SCM0180.png]

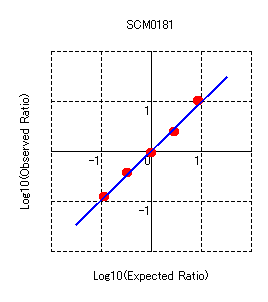

Supplement: Additional file 5 — Evaluation of 5,038 GSPs. A mini-website to browse plots similar to those shown in Additional data file 4 for all the 5,038 GSPs. [file 1471-2164-9-574-S5.zip › image/SCM0181.png]

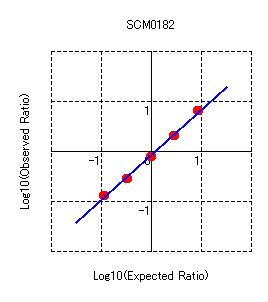

Supplement: Additional file 5 — Evaluation of 5,038 GSPs. A mini-website to browse plots similar to those shown in Additional data file 4 for all the 5,038 GSPs. [file 1471-2164-9-574-S5.zip › image/SCM0182.png]

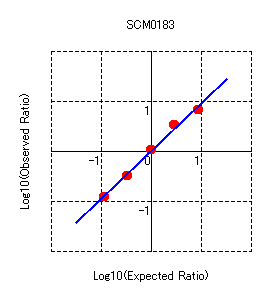

Supplement: Additional file 5 — Evaluation of 5,038 GSPs. A mini-website to browse plots similar to those shown in Additional data file 4 for all the 5,038 GSPs. [file 1471-2164-9-574-S5.zip › image/SCM0183.png]

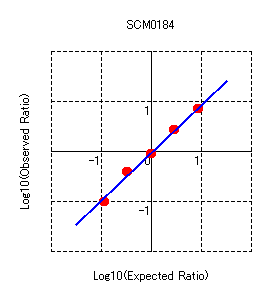

Supplement: Additional file 5 — Evaluation of 5,038 GSPs. A mini-website to browse plots similar to those shown in Additional data file 4 for all the 5,038 GSPs. [file 1471-2164-9-574-S5.zip › image/SCM0184.png]

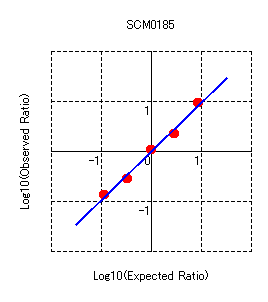

Supplement: Additional file 5 — Evaluation of 5,038 GSPs. A mini-website to browse plots similar to those shown in Additional data file 4 for all the 5,038 GSPs. [file 1471-2164-9-574-S5.zip › image/SCM0185.png]

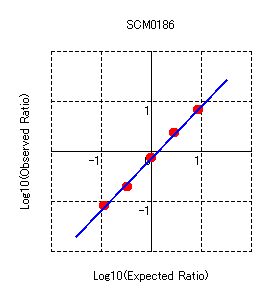

Supplement: Additional file 5 — Evaluation of 5,038 GSPs. A mini-website to browse plots similar to those shown in Additional data file 4 for all the 5,038 GSPs. [file 1471-2164-9-574-S5.zip › image/SCM0186.png]

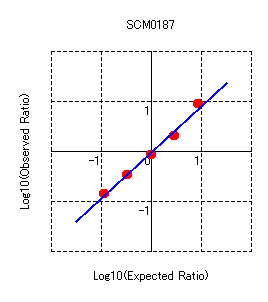

Supplement: Additional file 5 — Evaluation of 5,038 GSPs. A mini-website to browse plots similar to those shown in Additional data file 4 for all the 5,038 GSPs. [file 1471-2164-9-574-S5.zip › image/SCM0187.png]

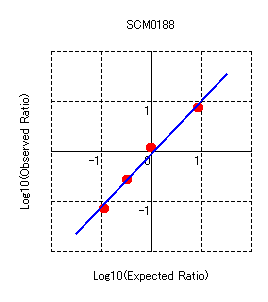

Supplement: Additional file 5 — Evaluation of 5,038 GSPs. A mini-website to browse plots similar to those shown in Additional data file 4 for all the 5,038 GSPs. [file 1471-2164-9-574-S5.zip › image/SCM0188.png]

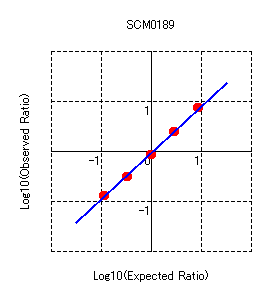

Supplement: Additional file 5 — Evaluation of 5,038 GSPs. A mini-website to browse plots similar to those shown in Additional data file 4 for all the 5,038 GSPs. [file 1471-2164-9-574-S5.zip › image/SCM0189.png]

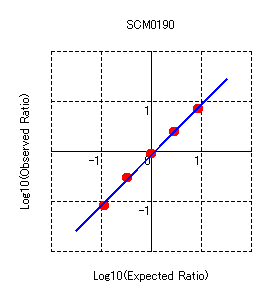

Supplement: Additional file 5 — Evaluation of 5,038 GSPs. A mini-website to browse plots similar to those shown in Additional data file 4 for all the 5,038 GSPs. [file 1471-2164-9-574-S5.zip › image/SCM0190.png]

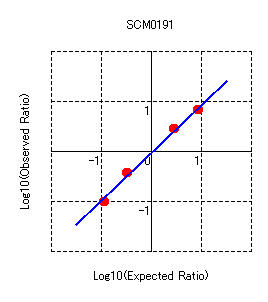

Supplement: Additional file 5 — Evaluation of 5,038 GSPs. A mini-website to browse plots similar to those shown in Additional data file 4 for all the 5,038 GSPs. [file 1471-2164-9-574-S5.zip › image/SCM0191.png]

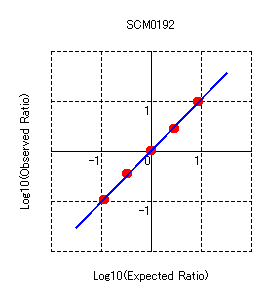

Supplement: Additional file 5 — Evaluation of 5,038 GSPs. A mini-website to browse plots similar to those shown in Additional data file 4 for all the 5,038 GSPs. [file 1471-2164-9-574-S5.zip › image/SCM0192.png]

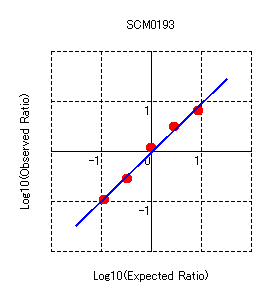

Supplement: Additional file 5 — Evaluation of 5,038 GSPs. A mini-website to browse plots similar to those shown in Additional data file 4 for all the 5,038 GSPs. [file 1471-2164-9-574-S5.zip › image/SCM0193.png]

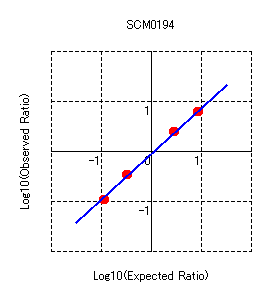

Supplement: Additional file 5 — Evaluation of 5,038 GSPs. A mini-website to browse plots similar to those shown in Additional data file 4 for all the 5,038 GSPs. [file 1471-2164-9-574-S5.zip › image/SCM0194.png]

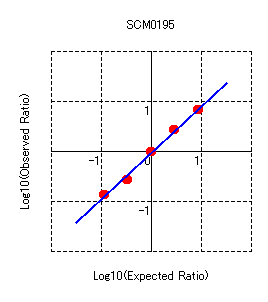

Supplement: Additional file 5 — Evaluation of 5,038 GSPs. A mini-website to browse plots similar to those shown in Additional data file 4 for all the 5,038 GSPs. [file 1471-2164-9-574-S5.zip › image/SCM0195.png]

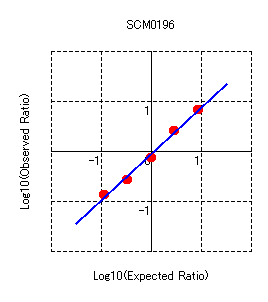

Supplement: Additional file 5 — Evaluation of 5,038 GSPs. A mini-website to browse plots similar to those shown in Additional data file 4 for all the 5,038 GSPs. [file 1471-2164-9-574-S5.zip › image/SCM0196.png]

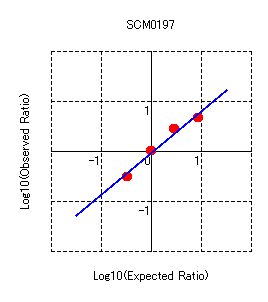

Supplement: Additional file 5 — Evaluation of 5,038 GSPs. A mini-website to browse plots similar to those shown in Additional data file 4 for all the 5,038 GSPs. [file 1471-2164-9-574-S5.zip › image/SCM0197.png]

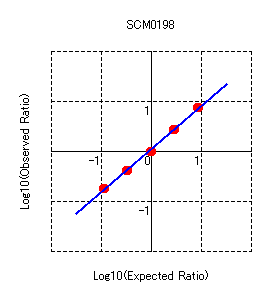

Supplement: Additional file 5 — Evaluation of 5,038 GSPs. A mini-website to browse plots similar to those shown in Additional data file 4 for all the 5,038 GSPs. [file 1471-2164-9-574-S5.zip › image/SCM0198.png]

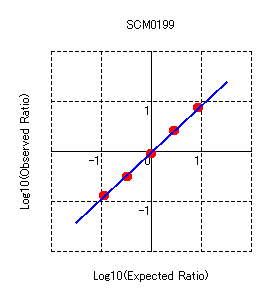

Supplement: Additional file 5 — Evaluation of 5,038 GSPs. A mini-website to browse plots similar to those shown in Additional data file 4 for all the 5,038 GSPs. [file 1471-2164-9-574-S5.zip › image/SCM0199.png]

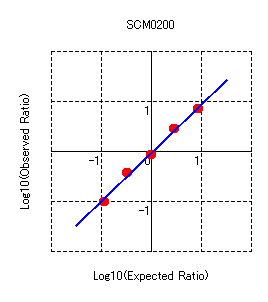

Supplement: Additional file 5 — Evaluation of 5,038 GSPs. A mini-website to browse plots similar to those shown in Additional data file 4 for all the 5,038 GSPs. [file 1471-2164-9-574-S5.zip › image/SCM0200.png]
