# Supplementary material for: Absolute quantification of the budding yeast transcriptome by means of competitive PCR between genomic and complementary DNAs
Source: BMC Genomics. 2008 Nov 29;9:574. doi: 10.1186/1471-2164-9-574 (PMC2612024; doi:10.1186/1471-2164-9-574)
Supplement: Additional file 5 — Evaluation of 5,038 GSPs. A mini-website to browse plots similar to those shown in Additional data file 4 for all the 5,038 GSPs. [file 1471-2164-9-574-S5.zip › index.html]

Additional data file 5 (Quantitativity of primers)


# Quantitativity of gene specific primers

Select primer
SCM0001
SCM0002
SCM0003
SCM0004
SCM0005
SCM0006
SCM0007
SCM0008
SCM0009
SCM0010
SCM0011
SCM0012
SCM0013
SCM0014
SCM0015
SCM0016
SCM0017
SCM0018
SCM0019
SCM0020
SCM0021
SCM0022
SCM0023
SCM0024
SCM0025
SCM0026
SCM0027
SCM0028
SCM0029
SCM0030
SCM0031
SCM0032
SCM0033
SCM0034
SCM0035
SCM0036
SCM0037
SCM0038
SCM0039
SCM0040
SCM0041
SCM0042
SCM0043
SCM0044
SCM0045
SCM0046
SCM0047
SCM0048
SCM0049
SCM0050
SCM0051
SCM0052
SCM0053
SCM0054
SCM0055
SCM0056
SCM0057
SCM0058
SCM0059
SCM0060
SCM0061
SCM0062
SCM0063
SCM0064
SCM0065
SCM0066
SCM0067
SCM0068
SCM0069
SCM0070
SCM0071
SCM0072
SCM0073
SCM0074
SCM0075
SCM0076
SCM0077
SCM0078
SCM0079
SCM0080
SCM0081
SCM0082
SCM0083
SCM0084
SCM0085
SCM0086
SCM0087
SCM0088
SCM0089
SCM0090
SCM0091
SCM0092
SCM0093
SCM0094
SCM0095
SCM0096
SCM0097
SCM0098
SCM0099
SCM0100
SCM0101
SCM0102
SCM0103
SCM0104
SCM0105
SCM0106
SCM0107
SCM0108
SCM0109
SCM0110
SCM0111
SCM0112
SCM0113
SCM0114
SCM0115
SCM0116
SCM0117
SCM0118
SCM0119
SCM0120
SCM0121
SCM0122
SCM0123
SCM0124
SCM0125
SCM0126
SCM0127
SCM0128
SCM0129
SCM0130
SCM0131
SCM0132
SCM0133
SCM0134
SCM0135
SCM0136
SCM0137
SCM0138
SCM0139
SCM0140
SCM0141
SCM0142
SCM0143
SCM0144
SCM0145
SCM0146
SCM0147
SCM0148
SCM0149
SCM0150
SCM0151
SCM0152
SCM0153
SCM0154
SCM0155
SCM0156
SCM0157
SCM0158
SCM0159
SCM0160
SCM0161
SCM0162
SCM0163
SCM0164
SCM0165
SCM0166
SCM0167
SCM0168
SCM0169
SCM0170
SCM0171
SCM0172
SCM0173
SCM0174
SCM0175
SCM0176
SCM0177
SCM0178
SCM0179
SCM0180
SCM0181
SCM0182
SCM0183
SCM0184
SCM0185
SCM0186
SCM0187
SCM0188
SCM0189
SCM0190
SCM0191
SCM0192
SCM0193
SCM0194
SCM0195
SCM0196
SCM0197
SCM0198
SCM0199
SCM0200
SCM0201
SCM0202
SCM0203
SCM0204
SCM0205
SCM0206
SCM0207
SCM0208
SCM0209
SCM0210
SCM0211
SCM0212
SCM0213
SCM0214
SCM0215
SCM0216
SCM0217
SCM0218
SCM0219
SCM0220
SCM0221
SCM0222
SCM0223
SCM0224
SCM0225
SCM0226
SCM0227
SCM0228
SCM0229
SCM0230
SCM0231
SCM0232
SCM0233
SCM0234
SCM0235
SCM0236
SCM0237
SCM0238
SCM0239
SCM0240
SCM0241
SCM0242
SCM0243
SCM0244
SCM0245
SCM0246
SCM0247
SCM0248
SCM0249
SCM0250
SCM0251
SCM0252
SCM0253
SCM0254
SCM0255
SCM0256
SCM0257
SCM0258
SCM0259
SCM0260
SCM0261
SCM0262
SCM0263
SCM0264
SCM0265
SCM0266
SCM0267
SCM0268
SCM0269
SCM0270
SCM0271
SCM0272
SCM0273
SCM0274
SCM0275
SCM0276
SCM0277
SCM0278
SCM0279
SCM0280
SCM0281
SCM0282
SCM0283
SCM0284
SCM0285
SCM0286
SCM0287
SCM0288
SCM0289
SCM0290
SCM0291
SCM0292
SCM0293
SCM0294
SCM0295
SCM0296
SCM0297
SCM0298
SCM0299
SCM0300
SCM0301
SCM0302
SCM0303
SCM0304
SCM0305
SCM0306
SCM0307
SCM0308
SCM0309
SCM0310
SCM0311
SCM0312
SCM0313
SCM0314
SCM0315
SCM0316
SCM0317
SCM0318
SCM0319
SCM0320
SCM0321
SCM0322
SCM0323
SCM0324
SCM0325
SCM0326
SCM0327
SCM0328
SCM0329
SCM0330
SCM0331
SCM0332
SCM0333
SCM0334
SCM0335
SCM0336
SCM0337
SCM0338
SCM0339
SCM0340
SCM0341
SCM0342
SCM0343
SCM0344
SCM0345
SCM0346
SCM0347
SCM0348
SCM0349
SCM0350
SCM0351
SCM0352
SCM0353
SCM0354
SCM0355
SCM0356
SCM0357
SCM0358
SCM0359
SCM0360
SCM0361
SCM0362
SCM0363
SCM0364
SCM0365
SCM0366
SCM0367
SCM0368
SCM0369
SCM0370
SCM0371
SCM0372
SCM0373
SCM0374
SCM0375
SCM0376
SCM0377
SCM0378
SCM0379
SCM0380
SCM0381
SCM0382
SCM0383
SCM0384
SCM0385
SCM0386
SCM0387
SCM0388
SCM0389
SCM0390
SCM0391
SCM0392
SCM0393
SCM0394
SCM0395
SCM0396
SCM0397
SCM0398
SCM0399
SCM0400
SCM0401
SCM0402
SCM0403
SCM0404
SCM0405
SCM0406
SCM0407
SCM0408
SCM0409
SCM0410
SCM0411
SCM0412
SCM0413
SCM0414
SCM0415
SCM0416
SCM0417
SCM0418
SCM0419
SCM0420
SCM0421
SCM0422
SCM0423
SCM0424
SCM0425
SCM0426
SCM0427
SCM0428
SCM0429
SCM0430
SCM0431
SCM0432
SCM0433
SCM0434
SCM0435
SCM0436
SCM0437
SCM0438
SCM0439
SCM0440
SCM0441
SCM0442
SCM0443
SCM0444
SCM0445
SCM0446
SCM0447
SCM0448
SCM0449
SCM0450
SCM0451
SCM0452
SCM0453
SCM0454
SCM0455
SCM0456
SCM0457
SCM0458
SCM0459
SCM0460
SCM0461
SCM0462
SCM0463
SCM0464
SCM0465
SCM0466
SCM0467
SCM0468
SCM0469
SCM0470
SCM0471
SCM0472
SCM0473
SCM0474
SCM0475
SCM0476
SCM0477
SCM0478
SCM0479
SCM0480
SCM0481
SCM0482
SCM0483
SCM0484
SCM0485
SCM0486
SCM0487
SCM0488
SCM0489
SCM0490
SCM0491
SCM0492
SCM0493
SCM0494
SCM0495
SCM0496
SCM0497
SCM0498
SCM0499
SCM0500
SCM0501
SCM0502
SCM0503
SCM0504
SCM0505
SCM0506
SCM0507
SCM0508
SCM0509
SCM0510
SCM0511
SCM0512
SCM0513
SCM0514
SCM0515
SCM0516
SCM0517
SCM0518
SCM0519
SCM0520
SCM0521
SCM0522
SCM0523
SCM0524
SCM0525
SCM0526
SCM0527
SCM0528
SCM0529
SCM0530
SCM0531
SCM0532
SCM0533
SCM0534
SCM0535
SCM0536
SCM0537
SCM0538
SCM0539
SCM0540
SCM0541
SCM0542
SCM0543
SCM0544
SCM0545
SCM0546
SCM0547
SCM0548
SCM0549
SCM0550
SCM0551
SCM0552
SCM0553
SCM0554
SCM0555
SCM0556
SCM0557
SCM0558
SCM0559
SCM0560
SCM0561
SCM0562
SCM0563
SCM0564
SCM0565
SCM0566
SCM0567
SCM0568
SCM0569
SCM0570
SCM0571
SCM0572
SCM0573
SCM0574
SCM0575
SCM0576
SCM0577
SCM0578
SCM0579
SCM0580
SCM0581
SCM0582
SCM0583
SCM0584
SCM0585
SCM0586
SCM0587
SCM0588
SCM0589
SCM0590
SCM0591
SCM0592
SCM0593
SCM0594
SCM0595
SCM0596
SCM0597
SCM0598
SCM0599
SCM0600
SCM0601
SCM0602
SCM0603
SCM0604
SCM0605
SCM0606
SCM0607
SCM0608
SCM0609
SCM0610
SCM0611
SCM0612
SCM0613
SCM0614
SCM0615
SCM0616
SCM0617
SCM0618
SCM0619
SCM0620
SCM0621
SCM0622
SCM0623
SCM0624
SCM0625
SCM0626
SCM0627
SCM0628
SCM0629
SCM0630
SCM0631
SCM0632
SCM0633
SCM0634
SCM0635
SCM0636
SCM0637
SCM0638
SCM0639
SCM0640
SCM0641
SCM0642
SCM0643
SCM0644
SCM0645
SCM0646
SCM0647
SCM0648
SCM0649
SCM0650
SCM0651
SCM0652
SCM0653
SCM0654
SCM0655
SCM0656
SCM0657
SCM0658
SCM0659
SCM0660
SCM0661
SCM0662
SCM0663
SCM0664
SCM0665
SCM0666
SCM0667
SCM0668
SCM0669
SCM0670
SCM0671
SCM0672
SCM0673
SCM0674
SCM0675
SCM0676
SCM0677
SCM0678
SCM0679
SCM0680
SCM0681
SCM0682
SCM0683
SCM0684
SCM0685
SCM0686
SCM0687
SCM0688
SCM0689
SCM0690
SCM0691
SCM0692
SCM0693
SCM0694
SCM0695
SCM0696
SCM0697
SCM0698
SCM0699
SCM0700
SCM0701
SCM0702
SCM0703
SCM0704
SCM0705
SCM0706
SCM0707
SCM0708
SCM0709
SCM0710
SCM0711
SCM0712
SCM0713
SCM0714
SCM0715
SCM0716
SCM0717
SCM0718
SCM0719
SCM0720
SCM0721
SCM0722
SCM0723
SCM0724
SCM0725
SCM0726
SCM0727
SCM0728
SCM0729
SCM0730
SCM0731
SCM0732
SCM0733
SCM0734
SCM0735
SCM0736
SCM0737
SCM0738
SCM0739
SCM0740
SCM0741
SCM0742
SCM0743
SCM0744
SCM0745
SCM0746
SCM0747
SCM0748
SCM0749
SCM0750
SCM0751
SCM0752
SCM0753
SCM0754
SCM0755
SCM0756
SCM0757
SCM0758
SCM0759
SCM0760
SCM0761
SCM0762
SCM0763
SCM0764
SCM0765
SCM0766
SCM0767
SCM0768
SCM0769
SCM0770
SCM0771
SCM0772
SCM0773
SCM0774
SCM0775
SCM0776
SCM0777
SCM0778
SCM0779
SCM0780
SCM0781
SCM0782
SCM0783
SCM0784
SCM0785
SCM0786
SCM0787
SCM0788
SCM0789
SCM0790
SCM0791
SCM0792
SCM0793
SCM0794
SCM0795
SCM0796
SCM0797
SCM0798
SCM0799
SCM0800
SCM0801
SCM0802
SCM0803
SCM0804
SCM0805
SCM0806
SCM0807
SCM0808
SCM0809
SCM0810
SCM0811
SCM0812
SCM0813
SCM0814
SCM0815
SCM0816
SCM0817
SCM0818
SCM0819
SCM0820
SCM0821
SCM0822
SCM0823
SCM0824
SCM0825
SCM0826
SCM0827
SCM0828
SCM0829
SCM0830
SCM0831
SCM0832
SCM0833
SCM0834
SCM0835
SCM0836
SCM0837
SCM0838
SCM0839
SCM0840
SCM0841
SCM0842
SCM0843
SCM0844
SCM0845
SCM0846
SCM0847
SCM0848
SCM0849
SCM0850
SCM0851
SCM0852
SCM0853
SCM0854
SCM0855
SCM0856
SCM0857
SCM0858
SCM0859
SCM0860
SCM0861
SCM0862
SCM0863
SCM0864
SCM0865
SCM0866
SCM0867
SCM0868
SCM0869
SCM0870
SCM0871
SCM0872
SCM0873
SCM0874
SCM0875
SCM0876
SCM0877
SCM0878
SCM0879
SCM0880
SCM0881
SCM0882
SCM0883
SCM0884
SCM0885
SCM0886
SCM0887
SCM0888
SCM0889
SCM0890
SCM0891
SCM0892
SCM0893
SCM0894
SCM0895
SCM0896
SCM0897
SCM0898
SCM0899
SCM0900
SCM0901
SCM0902
SCM0903
SCM0904
SCM0905
SCM0906
SCM0907
SCM0908
SCM0909
SCM0910
SCM0911
SCM0912
SCM0913
SCM0914
SCM0915
SCM0916
SCM0917
SCM0918
SCM0919
SCM0920
SCM0921
SCM0922
SCM0923
SCM0924
SCM0925
SCM0926
SCM0927
SCM0928
SCM0929
SCM0930
SCM0931
SCM0932
SCM0933
SCM0934
SCM0935
SCM0936
SCM0937
SCM0938
SCM0939
SCM0940
SCM0941
SCM0942
SCM0943
SCM0944
SCM0945
SCM0946
SCM0947
SCM0948
SCM0949
SCM0950
SCM0951
SCM0952
SCM0953
SCM0954
SCM0955
SCM0956
SCM0957
SCM0958
SCM0959
SCM0960
SCM0961
SCM0962
SCM0963
SCM0964
SCM0965
SCM0966
SCM0967
SCM0968
SCM0969
SCM0970
SCM0971
SCM0972
SCM0973
SCM0974
SCM0975
SCM0976
SCM0977
SCM0978
SCM0979
SCM0980
SCM0981
SCM0982
SCM0983
SCM0984
SCM0985
SCM0986
SCM0987
SCM0988
SCM0989
SCM0990
SCM0991
SCM0992
SCM0993
SCM0994
SCM0995
SCM0996
SCM0997
SCM0998
SCM0999
SCM1000
SCM1001
SCM1002
SCM1003
SCM1004
SCM1005
SCM1006
SCM1007
SCM1008
SCM1009
SCM1010
SCM1011
SCM1012
SCM1013
SCM1014
SCM1015
SCM1016
SCM1017
SCM1018
SCM1019
SCM1020
SCM1021
SCM1022
SCM1023
SCM1024
SCM1025
SCM1026
SCM1027
SCM1028
SCM1029
SCM1030
SCM1031
SCM1032
SCM1033
SCM1034
SCM1035
SCM1036
SCM1037
SCM1038
SCM1039
SCM1040
SCM1041
SCM1042
SCM1043
SCM1044
SCM1045
SCM1046
SCM1047
SCM1048
SCM1049
SCM1050
SCM1051
SCM1052
SCM1053
SCM1054
SCM1055
SCM1056
SCM1057
SCM1058
SCM1059
SCM1060
SCM1061
SCM1062
SCM1063
SCM1064
SCM1065
SCM1066
SCM1067
SCM1068
SCM1069
SCM1070
SCM1071
SCM1072
SCM1073
SCM1074
SCM1075
SCM1076
SCM1077
SCM1078
SCM1079
SCM1080
SCM1081
SCM1082
SCM1083
SCM1084
SCM1085
SCM1086
SCM1087
SCM1088
SCM1089
SCM1090
SCM1091
SCM1092
SCM1093
SCM1094
SCM1095
SCM1096
SCM1097
SCM1098
SCM1099
SCM1100
SCM1101
SCM1102
SCM1103
SCM1104
SCM1105
SCM1106
SCM1107
SCM1108
SCM1109
SCM1110
SCM1111
SCM1112
SCM1113
SCM1114
SCM1115
SCM1116
SCM1117
SCM1118
SCM1119
SCM1120
SCM1121
SCM1122
SCM1123
SCM1124
SCM1125
SCM1126
SCM1127
SCM1128
SCM1129
SCM1130
SCM1131
SCM1132
SCM1133
SCM1134
SCM1135
SCM1136
SCM1137
SCM1138
SCM1139
SCM1140
SCM1141
SCM1142
SCM1143
SCM1144
SCM1145
SCM1146
SCM1147
SCM1148
SCM1149
SCM1150
SCM1151
SCM1152
SCM1153
SCM1154
SCM1155
SCM1156
SCM1157
SCM1158
SCM1159
SCM1160
SCM1161
SCM1162
SCM1163
SCM1164
SCM1165
SCM1166
SCM1167
SCM1168
SCM1169
SCM1170
SCM1171
SCM1172
SCM1173
SCM1174
SCM1175
SCM1176
SCM1177
SCM1178
SCM1179
SCM1180
SCM1181
SCM1182
SCM1183
SCM1184
SCM1185
SCM1186
SCM1187
SCM1188
SCM1189
SCM1190
SCM1191
SCM1192
SCM1193
SCM1194
SCM1195
SCM1196
SCM1197
SCM1198
SCM1199
SCM1200
SCM1201
SCM1202
SCM1203
SCM1204
SCM1205
SCM1206
SCM1207
SCM1208
SCM1209
SCM1210
SCM1211
SCM1212
SCM1213
SCM1214
SCM1215
SCM1216
SCM1217
SCM1218
SCM1219
SCM1220
SCM1221
SCM1222
SCM1223
SCM1224
SCM1225
SCM1226
SCM1227
SCM1228
SCM1229
SCM1230
SCM1231
SCM1232
SCM1233
SCM1234
SCM1235
SCM1236
SCM1237
SCM1238
SCM1239
SCM1240
SCM1241
SCM1242
SCM1243
SCM1244
SCM1245
SCM1246
SCM1247
SCM1248
SCM1249
SCM1250
SCM1251
SCM1252
SCM1253
SCM1254
SCM1255
SCM1256
SCM1257
SCM1258
SCM1259
SCM1260
SCM1261
SCM1262
SCM1263
SCM1264
SCM1265
SCM1266
SCM1267
SCM1268
SCM1269
SCM1270
SCM1271
SCM1272
SCM1273
SCM1274
SCM1275
SCM1276
SCM1277
SCM1278
SCM1279
SCM1280
SCM1281
SCM1282
SCM1283
SCM1284
SCM1285
SCM1286
SCM1287
SCM1288
SCM1289
SCM1290
SCM1291
SCM1292
SCM1293
SCM1294
SCM1295
SCM1296
SCM1297
SCM1298
SCM1299
SCM1300
SCM1301
SCM1302
SCM1303
SCM1304
SCM1305
SCM1306
SCM1307
SCM1308
SCM1309
SCM1310
SCM1311
SCM1312
SCM1313
SCM1314
SCM1315
SCM1316
SCM1317
SCM1318
SCM1319
SCM1320
SCM1321
SCM1322
SCM1323
SCM1324
SCM1325
SCM1326
SCM1327
SCM1328
SCM1329
SCM1330
SCM1331
SCM1332
SCM1333
SCM1334
SCM1335
SCM1336
SCM1337
SCM1338
SCM1339
SCM1340
SCM1341
SCM1342
SCM1343
SCM1344
SCM1345
SCM1346
SCM1347
SCM1348
SCM1349
SCM1350
SCM1351
SCM1352
SCM1353
SCM1354
SCM1355
SCM1356
SCM1357
SCM1358
SCM1359
SCM1360
SCM1361
SCM1362
SCM1363
SCM1364
SCM1365
SCM1366
SCM1367
SCM1368
SCM1369
SCM1370
SCM1371
SCM1372
SCM1373
SCM1374
SCM1375
SCM1376
SCM1377
SCM1378
SCM1379
SCM1380
SCM1381
SCM1382
SCM1383
SCM1384
SCM1385
SCM1386
SCM1387
SCM1388
SCM1389
SCM1390
SCM1391
SCM1392
SCM1393
SCM1394
SCM1395
SCM1396
SCM1397
SCM1398
SCM1399
SCM1400
SCM1401
SCM1402
SCM1403
SCM1404
SCM1405
SCM1406
SCM1407
SCM1408
SCM1409
SCM1410
SCM1411
SCM1412
SCM1413
SCM1414
SCM1415
SCM1416
SCM1417
SCM1418
SCM1419
SCM1420
SCM1421
SCM1422
SCM1423
SCM1424
SCM1425
SCM1426
SCM1427
SCM1428
SCM1429
SCM1430
SCM1431
SCM1432
SCM1433
SCM1434
SCM1435
SCM1436
SCM1437
SCM1438
SCM1439
SCM1440
SCM1441
SCM1442
SCM1443
SCM1444
SCM1445
SCM1446
SCM1447
SCM1448
SCM1449
SCM1450
SCM1451
SCM1452
SCM1453
SCM1454
SCM1455
SCM1456
SCM1457
SCM1458
SCM1459
SCM1460
SCM1461
SCM1462
SCM1463
SCM1464
SCM1465
SCM1466
SCM1467
SCM1468
SCM1469
SCM1470
SCM1471
SCM1472
SCM1473
SCM1474
SCM1475
SCM1476
SCM1477
SCM1478
SCM1479
SCM1480
SCM1481
SCM1482
SCM1483
SCM1484
SCM1485
SCM1486
SCM1487
SCM1488
SCM1489
SCM1490
SCM1491
SCM1492
SCM1493
SCM1494
SCM1495
SCM1496
SCM1497
SCM1498
SCM1499
SCM1500
SCM1501
SCM1502
SCM1503
SCM1504
SCM1505
SCM1506
SCM1507
SCM1508
SCM1509
SCM1510
SCM1511
SCM1512
SCM1513
SCM1514
SCM1515
SCM1516
SCM1517
SCM1518
SCM1519
SCM1520
SCM1521
SCM1522
SCM1523
SCM1524
SCM1525
SCM1526
SCM1527
SCM1528
SCM1529
SCM1530
SCM1531
SCM1532
SCM1533
SCM1534
SCM1535
SCM1536
SCM1537
SCM1538
SCM1539
SCM1540
SCM1541
SCM1542
SCM1543
SCM1544
SCM1545
SCM1546
SCM1547
SCM1548
SCM1549
SCM1550
SCM1551
SCM1552
SCM1553
SCM1554
SCM1555
SCM1556
SCM1557
SCM1558
SCM1559
SCM1560
SCM1561
SCM1562
SCM1563
SCM1564
SCM1565
SCM1566
SCM1567
SCM1568
SCM1569
SCM1570
SCM1571
SCM1572
SCM1573
SCM1574
SCM1575
SCM1576
SCM1577
SCM1578
SCM1579
SCM1580
SCM1581
SCM1582
SCM1583
SCM1584
SCM1585
SCM1586
SCM1587
SCM1588
SCM1589
SCM1590
SCM1591
SCM1592
SCM1593
SCM1594
SCM1595
SCM1596
SCM1597
SCM1598
SCM1599
SCM1600
SCM1601
SCM1602
SCM1603
SCM1604
SCM1605
SCM1606
SCM1607
SCM1608
SCM1609
SCM1610
SCM1611
SCM1612
SCM1613
SCM1614
SCM1615
SCM1616
SCM1617
SCM1618
SCM1619
SCM1620
SCM1621
SCM1622
SCM1623
SCM1624
SCM1625
SCM1626
SCM1627
SCM1628
SCM1629
SCM1630
SCM1631
SCM1632
SCM1633
SCM1634
SCM1635
SCM1636
SCM1637
SCM1638
SCM1639
SCM1640
SCM1641
SCM1642
SCM1643
SCM1644
SCM1645
SCM1646
SCM1647
SCM1648
SCM1649
SCM1650
SCM1651
SCM1652
SCM1653
SCM1654
SCM1655
SCM1656
SCM1657
SCM1658
SCM1659
SCM1660
SCM1661
SCM1662
SCM1663
SCM1664
SCM1665
SCM1666
SCM1667
SCM1668
SCM1669
SCM1670
SCM1671
SCM1672
SCM1673
SCM1674
SCM1675
SCM1676
SCM1677
SCM1678
SCM1679
SCM1680
SCM1681
SCM1682
SCM1683
SCM1684
SCM1685
SCM1686
SCM1687
SCM1688
SCM1689
SCM1690
SCM1691
SCM1692
SCM1693
SCM1694
SCM1695
SCM1696
SCM1697
SCM1698
SCM1699
SCM1700
SCM1701
SCM1702
SCM1703
SCM1704
SCM1705
SCM1706
SCM1707
SCM1708
SCM1709
SCM1710
SCM1711
SCM1712
SCM1713
SCM1714
SCM1715
SCM1716
SCM1717
SCM1718
SCM1719
SCM1720
SCM1721
SCM1722
SCM1723
SCM1724
SCM1725
SCM1726
SCM1727
SCM1728
SCM1729
SCM1730
SCM1731
SCM1732
SCM1733
SCM1734
SCM1735
SCM1736
SCM1737
SCM1738
SCM1739
SCM1740
SCM1741
SCM1742
SCM1743
SCM1744
SCM1745
SCM1746
SCM1747
SCM1748
SCM1749
SCM1750
SCM1751
SCM1752
SCM1753
SCM1754
SCM1755
SCM1756
SCM1757
SCM1758
SCM1759
SCM1760
SCM1761
SCM1762
SCM1763
SCM1764
SCM1765
SCM1766
SCM1767
SCM1768
SCM1769
SCM1770
SCM1771
SCM1772
SCM1773
SCM1774
SCM1775
SCM1776
SCM1777
SCM1778
SCM1779
SCM1780
SCM1781
SCM1782
SCM1783
SCM1784
SCM1785
SCM1786
SCM1787
SCM1788
SCM1789
SCM1790
SCM1791
SCM1792
SCM1793
SCM1794
SCM1795
SCM1796
SCM1797
SCM1798
SCM1799
SCM1800
SCM1801
SCM1802
SCM1803
SCM1804
SCM1805
SCM1806
SCM1807
SCM1808
SCM1809
SCM1810
SCM1811
SCM1812
SCM1813
SCM1814
SCM1815
SCM1816
SCM1817
SCM1818
SCM1819
SCM1820
SCM1821
SCM1822
SCM1823
SCM1824
SCM1825
SCM1826
SCM1827
SCM1828
SCM1829
SCM1830
SCM1831
SCM1832
SCM1833
SCM1834
SCM1835
SCM1836
SCM1837
SCM1838
SCM1839
SCM1840
SCM1841
SCM1842
SCM1843
SCM1844
SCM1845
SCM1846
SCM1847
SCM1848
SCM1849
SCM1850
SCM1851
SCM1852
SCM1853
SCM1854
SCM1855
SCM1856
SCM1857
SCM1858
SCM1859
SCM1860
SCM1861
SCM1862
SCM1863
SCM1864
SCM1865
SCM1866
SCM1867
SCM1868
SCM1869
SCM1870
SCM1871
SCM1872
SCM1873
SCM1874
SCM1875
SCM1876
SCM1877
SCM1878
SCM1879
SCM1880
SCM1881
SCM1882
SCM1883
SCM1884
SCM1885
SCM1886
SCM1887
SCM1888
SCM1889
SCM1890
SCM1891
SCM1892
SCM1893
SCM1894
SCM1895
SCM1896
SCM1897
SCM1898
SCM1899
SCM1900
SCM1901
SCM1902
SCM1903
SCM1904
SCM1905
SCM1906
SCM1907
SCM1908
SCM1909
SCM1910
SCM1911
SCM1912
SCM1913
SCM1914
SCM1915
SCM1916
SCM1917
SCM1918
SCM1919
SCM1920
SCM1921
SCM1922
SCM1923
SCM1924
SCM1925
SCM1926
SCM1927
SCM1928
SCM1929
SCM1930
SCM1931
SCM1932
SCM1933
SCM1934
SCM1935
SCM1936
SCM1937
SCM1938
SCM1939
SCM1940
SCM1941
SCM1942
SCM1943
SCM1944
SCM1945
SCM1946
SCM1947
SCM1948
SCM1949
SCM1950
SCM1951
SCM1952
SCM1953
SCM1954
SCM1955
SCM1956
SCM1957
SCM1958
SCM1959
SCM1960
SCM1961
SCM1962
SCM1963
SCM1964
SCM1965
SCM1966
SCM1967
SCM1968
SCM1969
SCM1970
SCM1971
SCM1972
SCM1973
SCM1974
SCM1975
SCM1976
SCM1977
SCM1978
SCM1979
SCM1980
SCM1981
SCM1982
SCM1983
SCM1984
SCM1985
SCM1986
SCM1987
SCM1988
SCM1989
SCM1990
SCM1991
SCM1992
SCM1993
SCM1994
SCM1995
SCM1996
SCM1997
SCM1998
SCM1999
SCM2000
SCM2001
SCM2002
SCM2003
SCM2004
SCM2005
SCM2006
SCM2007
SCM2008
SCM2009
SCM2010
SCM2011
SCM2012
SCM2013
SCM2014
SCM2015
SCM2016
SCM2017
SCM2018
SCM2019
SCM2020
SCM2021
SCM2022
SCM2023
SCM2024
SCM2025
SCM2026
SCM2027
SCM2028
SCM2029
SCM2030
SCM2031
SCM2032
SCM2033
SCM2034
SCM2035
SCM2036
SCM2037
SCM2038
SCM2039
SCM2040
SCM2041
SCM2042
SCM2043
SCM2044
SCM2045
SCM2046
SCM2047
SCM2048
SCM2049
SCM2050
SCM2051
SCM2052
SCM2053
SCM2054
SCM2055
SCM2056
SCM2057
SCM2058
SCM2059
SCM2060
SCM2061
SCM2062
SCM2063
SCM2064
SCM2065
SCM2066
SCM2067
SCM2068
SCM2069
SCM2070
SCM2071
SCM2072
SCM2073
SCM2074
SCM2075
SCM2076
SCM2077
SCM2078
SCM2079
SCM2080
SCM2081
SCM2082
SCM2083
SCM2084
SCM2085
SCM2086
SCM2087
SCM2088
SCM2089
SCM2090
SCM2091
SCM2092
SCM2093
SCM2094
SCM2095
SCM2096
SCM2097
SCM2098
SCM2099
SCM2100
SCM2101
SCM2102
SCM2103
SCM2104
SCM2105
SCM2106
SCM2107
SCM2108
SCM2109
SCM2110
SCM2111
SCM2112
SCM2113
SCM2114
SCM2115
SCM2116
SCM2117
SCM2118
SCM2119
SCM2120
SCM2121
SCM2122
SCM2123
SCM2124
SCM2125
SCM2126
SCM2127
SCM2128
SCM2129
SCM2130
SCM2131
SCM2132
SCM2133
SCM2134
SCM2135
SCM2136
SCM2137
SCM2138
SCM2139
SCM2140
SCM2141
SCM2142
SCM2143
SCM2144
SCM2145
SCM2146
SCM2147
SCM2148
SCM2149
SCM2150
SCM2151
SCM2152
SCM2153
SCM2154
SCM2155
SCM2156
SCM2157
SCM2158
SCM2159
SCM2160
SCM2161
SCM2162
SCM2163
SCM2164
SCM2165
SCM2166
SCM2167
SCM2168
SCM2169
SCM2170
SCM2171
SCM2172
SCM2173
SCM2174
SCM2175
SCM2176
SCM2177
SCM2178
SCM2179
SCM2180
SCM2181
SCM2182
SCM2183
SCM2184
SCM2185
SCM2186
SCM2187
SCM2188
SCM2189
SCM2190
SCM2191
SCM2192
SCM2193
SCM2194
SCM2195
SCM2196
SCM2197
SCM2198
SCM2199
SCM2200
SCM2201
SCM2202
SCM2203
SCM2204
SCM2205
SCM2206
SCM2207
SCM2208
SCM2209
SCM2210
SCM2211
SCM2212
SCM2213
SCM2214
SCM2215
SCM2216
SCM2217
SCM2218
SCM2219
SCM2220
SCM2221
SCM2222
SCM2223
SCM2224
SCM2225
SCM2226
SCM2227
SCM2228
SCM2229
SCM2230
SCM2231
SCM2232
SCM2233
SCM2234
SCM2235
SCM2236
SCM2237
SCM2238
SCM2239
SCM2240
SCM2241
SCM2242
SCM2243
SCM2244
SCM2245
SCM2246
SCM2247
SCM2248
SCM2249
SCM2250
SCM2251
SCM2252
SCM2253
SCM2254
SCM2255
SCM2256
SCM2257
SCM2258
SCM2259
SCM2260
SCM2261
SCM2262
SCM2263
SCM2264
SCM2265
SCM2266
SCM2267
SCM2268
SCM2269
SCM2270
SCM2271
SCM2272
SCM2273
SCM2274
SCM2275
SCM2276
SCM2277
SCM2278
SCM2279
SCM2280
SCM2281
SCM2282
SCM2283
SCM2284
SCM2285
SCM2286
SCM2287
SCM2288
SCM2289
SCM2290
SCM2291
SCM2292
SCM2293
SCM2294
SCM2295
SCM2296
SCM2297
SCM2298
SCM2299
SCM2300
SCM2301
SCM2302
SCM2303
SCM2304
SCM2305
SCM2306
SCM2307
SCM2308
SCM2309
SCM2310
SCM2311
SCM2312
SCM2313
SCM2314
SCM2315
SCM2316
SCM2317
SCM2318
SCM2319
SCM2320
SCM2321
SCM2322
SCM2323
SCM2324
SCM2325
SCM2326
SCM2327
SCM2328
SCM2329
SCM2330
SCM2331
SCM2332
SCM2333
SCM2334
SCM2335
SCM2336
SCM2337
SCM2338
SCM2339
SCM2340
SCM2341
SCM2342
SCM2343
SCM2344
SCM2345
SCM2346
SCM2347
SCM2348
SCM2349
SCM2350
SCM2351
SCM2352
SCM2353
SCM2354
SCM2355
SCM2356
SCM2357
SCM2358
SCM2359
SCM2360
SCM2361
SCM2362
SCM2363
SCM2364
SCM2365
SCM2366
SCM2367
SCM2368
SCM2369
SCM2370
SCM2371
SCM2372
SCM2373
SCM2374
SCM2375
SCM2376
SCM2377
SCM2378
SCM2379
SCM2380
SCM2381
SCM2382
SCM2383
SCM2384
SCM2385
SCM2386
SCM2387
SCM2388
SCM2389
SCM2390
SCM2391
SCM2392
SCM2393
SCM2394
SCM2395
SCM2396
SCM2397
SCM2398
SCM2399
SCM2400
SCM2401
SCM2402
SCM2403
SCM2404
SCM2405
SCM2406
SCM2407
SCM2408
SCM2409
SCM2410
SCM2411
SCM2412
SCM2413
SCM2414
SCM2415
SCM2416
SCM2417
SCM2418
SCM2419
SCM2420
SCM2421
SCM2422
SCM2423
SCM2424
SCM2425
SCM2426
SCM2427
SCM2428
SCM2429
SCM2430
SCM2431
SCM2432
SCM2433
SCM2434
SCM2435
SCM2436
SCM2437
SCM2438
SCM2439
SCM2440
SCM2441
SCM2442
SCM2443
SCM2444
SCM2445
SCM2446
SCM2447
SCM2448
SCM2449
SCM2450
SCM2451
SCM2452
SCM2453
SCM2454
SCM2455
SCM2456
SCM2457
SCM2458
SCM2459
SCM2460
SCM2461
SCM2462
SCM2463
SCM2464
SCM2465
SCM2466
SCM2467
SCM2468
SCM2469
SCM2470
SCM2471
SCM2472
SCM2473
SCM2474
SCM2475
SCM2476
SCM2477
SCM2478
SCM2479
SCM2480
SCM2481
SCM2482
SCM2483
SCM2484
SCM2485
SCM2486
SCM2487
SCM2488
SCM2489
SCM2490
SCM2491
SCM2492
SCM2493
SCM2494
SCM2495
SCM2496
SCM2497
SCM2498
SCM2499
SCM2500
SCM2501
SCM2502
SCM2503
SCM2504
SCM2505
SCM2506
SCM2507
SCM2508
SCM2509
SCM2510
SCM2511
SCM2512
SCM2513
SCM2514
SCM2515
SCM2516
SCM2517
SCM2518
SCM2519
SCM2520
SCM2521
SCM2522
SCM2523
SCM2524
SCM2525
SCM2526
SCM2527
SCM2528
SCM2529
SCM2530
SCM2531
SCM2532
SCM2533
SCM2534
SCM2535
SCM2536
SCM2537
SCM2538
SCM2539
SCM2540
SCM2541
SCM2542
SCM2543
SCM2544
SCM2545
SCM2546
SCM2547
SCM2548
SCM2549
SCM2550
SCM2551
SCM2552
SCM2553
SCM2554
SCM2555
SCM2556
SCM2557
SCM2558
SCM2559
SCM2560
SCM2561
SCM2562
SCM2563
SCM2564
SCM2565
SCM2566
SCM2567
SCM2568
SCM2569
SCM2570
SCM2571
SCM2572
SCM2573
SCM2574
SCM2575
SCM2576
SCM2577
SCM2578
SCM2579
SCM2580
SCM2581
SCM2582
SCM2583
SCM2584
SCM2585
SCM2586
SCM2587
SCM2588
SCM2589
SCM2590
SCM2591
SCM2592
SCM2593
SCM2594
SCM2595
SCM2596
SCM2597
SCM2598
SCM2599
SCM2600
SCM2601
SCM2602
SCM2603
SCM2604
SCM2605
SCM2606
SCM2607
SCM2608
SCM2609
SCM2610
SCM2611
SCM2612
SCM2613
SCM2614
SCM2615
SCM2616
SCM2617
SCM2618
SCM2619
SCM2620
SCM2621
SCM2622
SCM2623
SCM2624
SCM2625
SCM2626
SCM2627
SCM2628
SCM2629
SCM2630
SCM2631
SCM2632
SCM2633
SCM2634
SCM2635
SCM2636
SCM2637
SCM2638
SCM2639
SCM2640
SCM2641
SCM2642
SCM2643
SCM2644
SCM2645
SCM2646
SCM2647
SCM2648
SCM2649
SCM2650
SCM2651
SCM2652
SCM2653
SCM2654
SCM2655
SCM2656
SCM2657
SCM2658
SCM2659
SCM2660
SCM2661
SCM2662
SCM2663
SCM2664
SCM2665
SCM2666
SCM2667
SCM2668
SCM2669
SCM2670
SCM2671
SCM2672
SCM2673
SCM2674
SCM2675
SCM2676
SCM2677
SCM2678
SCM2679
SCM2680
SCM2681
SCM2682
SCM2683
SCM2684
SCM2685
SCM2686
SCM2687
SCM2688
SCM2689
SCM2690
SCM2691
SCM2692
SCM2693
SCM2694
SCM2695
SCM2696
SCM2697
SCM2698
SCM2699
SCM2700
SCM2701
SCM2702
SCM2703
SCM2704
SCM2705
SCM2706
SCM2707
SCM2708
SCM2709
SCM2710
SCM2711
SCM2712
SCM2713
SCM2714
SCM2715
SCM2716
SCM2717
SCM2718
SCM2719
SCM2720
SCM2721
SCM2722
SCM2723
SCM2724
SCM2725
SCM2726
SCM2727
SCM2728
SCM2729
SCM2730
SCM2731
SCM2732
SCM2733
SCM2734
SCM2735
SCM2736
SCM2737
SCM2738
SCM2739
SCM2740
SCM2741
SCM2742
SCM2743
SCM2744
SCM2745
SCM2746
SCM2747
SCM2748
SCM2749
SCM2750
SCM2751
SCM2752
SCM2753
SCM2754
SCM2755
SCM2756
SCM2757
SCM2758
SCM2759
SCM2760
SCM2761
SCM2762
SCM2763
SCM2764
SCM2765
SCM2766
SCM2767
SCM2768
SCM2769
SCM2770
SCM2771
SCM2772
SCM2773
SCM2774
SCM2775
SCM2776
SCM2777
SCM2778
SCM2779
SCM2780
SCM2781
SCM2782
SCM2783
SCM2784
SCM2785
SCM2786
SCM2787
SCM2788
SCM2789
SCM2790
SCM2791
SCM2792
SCM2793
SCM2794
SCM2795
SCM2796
SCM2797
SCM2798
SCM2799
SCM2800
SCM2801
SCM2802
SCM2803
SCM2804
SCM2805
SCM2806
SCM2807
SCM2808
SCM2809
SCM2810
SCM2811
SCM2812
SCM2813
SCM2814
SCM2815
SCM2816
SCM2817
SCM2818
SCM2819
SCM2820
SCM2821
SCM2822
SCM2823
SCM2824
SCM2825
SCM2826
SCM2827
SCM2828
SCM2829
SCM2830
SCM2831
SCM2832
SCM2833
SCM2834
SCM2835
SCM2836
SCM2837
SCM2838
SCM2839
SCM2840
SCM2841
SCM2842
SCM2843
SCM2844
SCM2845
SCM2846
SCM2847
SCM2848
SCM2849
SCM2850
SCM2851
SCM2852
SCM2853
SCM2854
SCM2855
SCM2856
SCM2857
SCM2858
SCM2859
SCM2860
SCM2861
SCM2862
SCM2863
SCM2864
SCM2865
SCM2866
SCM2867
SCM2868
SCM2869
SCM2870
SCM2871
SCM2872
SCM2873
SCM2874
SCM2875
SCM2876
SCM2877
SCM2878
SCM2879
SCM2880
SCM2881
SCM2882
SCM2883
SCM2884
SCM2885
SCM2886
SCM2887
SCM2888
SCM2889
SCM2890
SCM2891
SCM2892
SCM2893
SCM2894
SCM2895
SCM2896
SCM2897
SCM2898
SCM2899
SCM2900
SCM2901
SCM2902
SCM2903
SCM2904
SCM2905
SCM2906
SCM2907
SCM2908
SCM2909
SCM2910
SCM2911
SCM2912
SCM2913
SCM2914
SCM2915
SCM2916
SCM2917
SCM2918
SCM2919
SCM2920
SCM2921
SCM2922
SCM2923
SCM2924
SCM2925
SCM2926
SCM2927
SCM2928
SCM2929
SCM2930
SCM2931
SCM2932
SCM2933
SCM2934
SCM2935
SCM2936
SCM2937
SCM2938
SCM2939
SCM2940
SCM2941
SCM2942
SCM2943
SCM2944
SCM2945
SCM2946
SCM2947
SCM2948
SCM2949
SCM2950
SCM2951
SCM2952
SCM2953
SCM2954
SCM2955
SCM2956
SCM2957
SCM2958
SCM2959
SCM2960
SCM2961
SCM2962
SCM2963
SCM2964
SCM2965
SCM2966
SCM2967
SCM2968
SCM2969
SCM2970
SCM2971
SCM2972
SCM2973
SCM2974
SCM2975
SCM2976
SCM2977
SCM2978
SCM2979
SCM2980
SCM2981
SCM2982
SCM2983
SCM2984
SCM2985
SCM2986
SCM2987
SCM2988
SCM2989
SCM2990
SCM2991
SCM2992
SCM2993
SCM2994
SCM2995
SCM2996
SCM2997
SCM2998
SCM2999
SCM3000
SCM3001
SCM3002
SCM3003
SCM3004
SCM3005
SCM3006
SCM3007
SCM3008
SCM3009
SCM3010
SCM3011
SCM3012
SCM3013
SCM3014
SCM3015
SCM3016
SCM3017
SCM3018
SCM3019
SCM3020
SCM3021
SCM3022
SCM3023
SCM3024
SCM3025
SCM3026
SCM3027
SCM3028
SCM3029
SCM3030
SCM3031
SCM3032
SCM3033
SCM3034
SCM3035
SCM3036
SCM3037
SCM3038
SCM3039
SCM3040
SCM3041
SCM3042
SCM3043
SCM3044
SCM3045
SCM3046
SCM3047
SCM3048
SCM3049
SCM3050
SCM3051
SCM3052
SCM3053
SCM3054
SCM3055
SCM3056
SCM3057
SCM3058
SCM3059
SCM3060
SCM3061
SCM3062
SCM3063
SCM3064
SCM3065
SCM3066
SCM3067
SCM3068
SCM3069
SCM3070
SCM3071
SCM3072
SCM3073
SCM3074
SCM3075
SCM3076
SCM3077
SCM3078
SCM3079
SCM3080
SCM3081
SCM3082
SCM3083
SCM3084
SCM3085
SCM3086
SCM3087
SCM3088
SCM3089
SCM3090
SCM3091
SCM3092
SCM3093
SCM3094
SCM3095
SCM3096
SCM3097
SCM3098
SCM3099
SCM3100
SCM3101
SCM3102
SCM3103
SCM3104
SCM3105
SCM3106
SCM3107
SCM3108
SCM3109
SCM3110
SCM3111
SCM3112
SCM3113
SCM3114
SCM3115
SCM3116
SCM3117
SCM3118
SCM3119
SCM3120
SCM3121
SCM3122
SCM3123
SCM3124
SCM3125
SCM3126
SCM3127
SCM3128
SCM3129
SCM3130
SCM3131
SCM3132
SCM3133
SCM3134
SCM3135
SCM3136
SCM3137
SCM3138
SCM3139
SCM3140
SCM3141
SCM3142
SCM3143
SCM3144
SCM3145
SCM3146
SCM3147
SCM3148
SCM3149
SCM3150
SCM3151
SCM3152
SCM3153
SCM3154
SCM3155
SCM3156
SCM3157
SCM3158
SCM3159
SCM3160
SCM3161
SCM3162
SCM3163
SCM3164
SCM3165
SCM3166
SCM3167
SCM3168
SCM3169
SCM3170
SCM3171
SCM3172
SCM3173
SCM3174
SCM3175
SCM3176
SCM3177
SCM3178
SCM3179
SCM3180
SCM3181
SCM3182
SCM3183
SCM3184
SCM3185
SCM3186
SCM3187
SCM3188
SCM3189
SCM3190
SCM3191
SCM3192
SCM3193
SCM3194
SCM3195
SCM3196
SCM3197
SCM3198
SCM3199
SCM3200
SCM3201
SCM3202
SCM3203
SCM3204
SCM3205
SCM3206
SCM3207
SCM3208
SCM3209
SCM3210
SCM3211
SCM3212
SCM3213
SCM3214
SCM3215
SCM3216
SCM3217
SCM3218
SCM3219
SCM3220
SCM3221
SCM3222
SCM3223
SCM3224
SCM3225
SCM3226
SCM3227
SCM3228
SCM3229
SCM3230
SCM3231
SCM3232
SCM3233
SCM3234
SCM3235
SCM3236
SCM3237
SCM3238
SCM3239
SCM3240
SCM3241
SCM3242
SCM3243
SCM3244
SCM3245
SCM3246
SCM3247
SCM3248
SCM3249
SCM3250
SCM3251
SCM3252
SCM3253
SCM3254
SCM3255
SCM3256
SCM3257
SCM3258
SCM3259
SCM3260
SCM3261
SCM3262
SCM3263
SCM3264
SCM3265
SCM3266
SCM3267
SCM3268
SCM3269
SCM3270
SCM3271
SCM3272
SCM3273
SCM3274
SCM3275
SCM3276
SCM3277
SCM3278
SCM3279
SCM3280
SCM3281
SCM3282
SCM3283
SCM3284
SCM3285
SCM3286
SCM3287
SCM3288
SCM3289
SCM3290
SCM3291
SCM3292
SCM3293
SCM3294
SCM3295
SCM3296
SCM3297
SCM3298
SCM3299
SCM3300
SCM3301
SCM3302
SCM3303
SCM3304
SCM3305
SCM3306
SCM3307
SCM3308
SCM3309
SCM3310
SCM3311
SCM3312
SCM3313
SCM3314
SCM3315
SCM3316
SCM3317
SCM3318
SCM3319
SCM3320
SCM3321
SCM3322
SCM3323
SCM3324
SCM3325
SCM3326
SCM3327
SCM3328
SCM3329
SCM3330
SCM3331
SCM3332
SCM3333
SCM3334
SCM3335
SCM3336
SCM3337
SCM3338
SCM3339
SCM3340
SCM3341
SCM3342
SCM3343
SCM3344
SCM3345
SCM3346
SCM3347
SCM3348
SCM3349
SCM3350
SCM3351
SCM3352
SCM3353
SCM3354
SCM3355
SCM3356
SCM3357
SCM3358
SCM3359
SCM3360
SCM3361
SCM3362
SCM3363
SCM3364
SCM3365
SCM3366
SCM3367
SCM3368
SCM3369
SCM3370
SCM3371
SCM3372
SCM3373
SCM3374
SCM3375
SCM3376
SCM3377
SCM3378
SCM3379
SCM3380
SCM3381
SCM3382
SCM3383
SCM3384
SCM3385
SCM3386
SCM3387
SCM3388
SCM3389
SCM3390
SCM3391
SCM3392
SCM3393
SCM3394
SCM3395
SCM3396
SCM3397
SCM3398
SCM3399
SCM3400
SCM3401
SCM3402
SCM3403
SCM3404
SCM3405
SCM3406
SCM3407
SCM3408
SCM3409
SCM3410
SCM3411
SCM3412
SCM3413
SCM3414
SCM3415
SCM3416
SCM3417
SCM3418
SCM3419
SCM3420
SCM3421
SCM3422
SCM3423
SCM3424
SCM3425
SCM3426
SCM3427
SCM3428
SCM3429
SCM3430
SCM3431
SCM3432
SCM3433
SCM3434
SCM3435
SCM3436
SCM3437
SCM3438
SCM3439
SCM3440
SCM3441
SCM3442
SCM3443
SCM3444
SCM3445
SCM3446
SCM3447
SCM3448
SCM3449
SCM3450
SCM3451
SCM3452
SCM3453
SCM3454
SCM3455
SCM3456
SCM3457
SCM3458
SCM3459
SCM3460
SCM3461
SCM3462
SCM3463
SCM3464
SCM3465
SCM3466
SCM3467
SCM3468
SCM3469
SCM3470
SCM3471
SCM3472
SCM3473
SCM3474
SCM3475
SCM3476
SCM3477
SCM3478
SCM3479
SCM3480
SCM3481
SCM3482
SCM3483
SCM3484
SCM3485
SCM3486
SCM3487
SCM3488
SCM3489
SCM3490
SCM3491
SCM3492
SCM3493
SCM3494
SCM3495
SCM3496
SCM3497
SCM3498
SCM3499
SCM3500
SCM3501
SCM3502
SCM3503
SCM3504
SCM3505
SCM3506
SCM3507
SCM3508
SCM3509
SCM3510
SCM3511
SCM3512
SCM3513
SCM3514
SCM3515
SCM3516
SCM3517
SCM3518
SCM3519
SCM3520
SCM3521
SCM3522
SCM3523
SCM3524
SCM3525
SCM3526
SCM3527
SCM3528
SCM3529
SCM3530
SCM3531
SCM3532
SCM3533
SCM3534
SCM3535
SCM3536
SCM3537
SCM3538
SCM3539
SCM3540
SCM3541
SCM3542
SCM3543
SCM3544
SCM3545
SCM3546
SCM3547
SCM3548
SCM3549
SCM3550
SCM3551
SCM3552
SCM3553
SCM3554
SCM3555
SCM3556
SCM3557
SCM3558
SCM3559
SCM3560
SCM3561
SCM3562
SCM3563
SCM3564
SCM3565
SCM3566
SCM3567
SCM3568
SCM3569
SCM3570
SCM3571
SCM3572
SCM3573
SCM3574
SCM3575
SCM3576
SCM3577
SCM3578
SCM3579
SCM3580
SCM3581
SCM3582
SCM3583
SCM3584
SCM3585
SCM3586
SCM3587
SCM3588
SCM3589
SCM3590
SCM3591
SCM3592
SCM3593
SCM3594
SCM3595
SCM3596
SCM3597
SCM3598
SCM3599
SCM3600
SCM3601
SCM3602
SCM3603
SCM3604
SCM3605
SCM3606
SCM3607
SCM3608
SCM3609
SCM3610
SCM3611
SCM3612
SCM3613
SCM3614
SCM3615
SCM3616
SCM3617
SCM3618
SCM3619
SCM3620
SCM3621
SCM3622
SCM3623
SCM3624
SCM3625
SCM3626
SCM3627
SCM3628
SCM3629
SCM3630
SCM3631
SCM3632
SCM3633
SCM3634
SCM3635
SCM3636
SCM3637
SCM3638
SCM3639
SCM3640
SCM3641
SCM3642
SCM3643
SCM3644
SCM3645
SCM3646
SCM3647
SCM3648
SCM3649
SCM3650
SCM3651
SCM3652
SCM3653
SCM3654
SCM3655
SCM3656
SCM3657
SCM3658
SCM3659
SCM3660
SCM3661
SCM3662
SCM3663
SCM3664
SCM3665
SCM3666
SCM3667
SCM3668
SCM3669
SCM3670
SCM3671
SCM3672
SCM3673
SCM3674
SCM3675
SCM3676
SCM3677
SCM3678
SCM3679
SCM3680
SCM3681
SCM3682
SCM3683
SCM3684
SCM3685
SCM3686
SCM3687
SCM3688
SCM3689
SCM3690
SCM3691
SCM3692
SCM3693
SCM3694
SCM3695
SCM3696
SCM3697
SCM3698
SCM3699
SCM3700
SCM3701
SCM3702
SCM3703
SCM3704
SCM3705
SCM3706
SCM3707
SCM3708
SCM3709
SCM3710
SCM3711
SCM3712
SCM3713
SCM3714
SCM3715
SCM3716
SCM3717
SCM3718
SCM3719
SCM3720
SCM3721
SCM3722
SCM3723
SCM3724
SCM3725
SCM3726
SCM3727
SCM3728
SCM3729
SCM3730
SCM3731
SCM3732
SCM3733
SCM3734
SCM3735
SCM3736
SCM3737
SCM3738
SCM3739
SCM3740
SCM3741
SCM3742
SCM3743
SCM3744
SCM3745
SCM3746
SCM3747
SCM3748
SCM3749
SCM3750
SCM3751
SCM3752
SCM3753
SCM3754
SCM3755
SCM3756
SCM3757
SCM3758
SCM3759
SCM3760
SCM3761
SCM3762
SCM3763
SCM3764
SCM3765
SCM3766
SCM3767
SCM3768
SCM3769
SCM3770
SCM3771
SCM3772
SCM3773
SCM3774
SCM3775
SCM3776
SCM3777
SCM3778
SCM3779
SCM3780
SCM3781
SCM3782
SCM3783
SCM3784
SCM3785
SCM3786
SCM3787
SCM3788
SCM3789
SCM3790
SCM3791
SCM3792
SCM3793
SCM3794
SCM3795
SCM3796
SCM3797
SCM3798
SCM3799
SCM3800
SCM3801
SCM3802
SCM3803
SCM3804
SCM3805
SCM3806
SCM3807
SCM3808
SCM3809
SCM3810
SCM3811
SCM3812
SCM3813
SCM3814
SCM3815
SCM3816
SCM3817
SCM3818
SCM3819
SCM3820
SCM3821
SCM3822
SCM3823
SCM3824
SCM3825
SCM3826
SCM3827
SCM3828
SCM3829
SCM3830
SCM3831
SCM3832
SCM3833
SCM3834
SCM3835
SCM3836
SCM3837
SCM3838
SCM3839
SCM3840
SCM3841
SCM3842
SCM3843
SCM3844
SCM3845
SCM3846
SCM3847
SCM3848
SCM3849
SCM3850
SCM3851
SCM3852
SCM3853
SCM3854
SCM3855
SCM3856
SCM3857
SCM3858
SCM3859
SCM3860
SCM3861
SCM3862
SCM3863
SCM3864
SCM3865
SCM3866
SCM3867
SCM3868
SCM3869
SCM3870
SCM3871
SCM3872
SCM3873
SCM3874
SCM3875
SCM3876
SCM3877
SCM3878
SCM3879
SCM3880
SCM3881
SCM3882
SCM3883
SCM3884
SCM3885
SCM3886
SCM3887
SCM3888
SCM3889
SCM3890
SCM3891
SCM3892
SCM3893
SCM3894
SCM3895
SCM3896
SCM3897
SCM3898
SCM3899
SCM3900
SCM3901
SCM3902
SCM3903
SCM3904
SCM3905
SCM3906
SCM3907
SCM3908
SCM3909
SCM3910
SCM3911
SCM3912
SCM3913
SCM3914
SCM3915
SCM3916
SCM3917
SCM3918
SCM3919
SCM3920
SCM3921
SCM3922
SCM3923
SCM3924
SCM3925
SCM3926
SCM3927
SCM3928
SCM3929
SCM3930
SCM3931
SCM3932
SCM3933
SCM3934
SCM3935
SCM3936
SCM3937
SCM3938
SCM3939
SCM3940
SCM3941
SCM3942
SCM3943
SCM3944
SCM3945
SCM3946
SCM3947
SCM3948
SCM3949
SCM3950
SCM3951
SCM3952
SCM3953
SCM3954
SCM3955
SCM3956
SCM3957
SCM3958
SCM3959
SCM3960
SCM3961
SCM3962
SCM3963
SCM3964
SCM3965
SCM3966
SCM3967
SCM3968
SCM3969
SCM3970
SCM3971
SCM3972
SCM3973
SCM3974
SCM3975
SCM3976
SCM3977
SCM3978
SCM3979
SCM3980
SCM3981
SCM3982
SCM3983
SCM3984
SCM3985
SCM3986
SCM3987
SCM3988
SCM3989
SCM3990
SCM3991
SCM3992
SCM3993
SCM3994
SCM3995
SCM3996
SCM3997
SCM3998
SCM3999
SCM4000
SCM4001
SCM4002
SCM4003
SCM4004
SCM4005
SCM4006
SCM4007
SCM4008
SCM4009
SCM4010
SCM4011
SCM4012
SCM4013
SCM4014
SCM4015
SCM4016
SCM4017
SCM4018
SCM4019
SCM4020
SCM4021
SCM4022
SCM4023
SCM4024
SCM4025
SCM4026
SCM4027
SCM4028
SCM4029
SCM4030
SCM4031
SCM4032
SCM4033
SCM4034
SCM4035
SCM4036
SCM4037
SCM4038
SCM4039
SCM4040
SCM4041
SCM4042
SCM4043
SCM4044
SCM4045
SCM4046
SCM4047
SCM4048
SCM4049
SCM4050
SCM4051
SCM4052
SCM4053
SCM4054
SCM4055
SCM4056
SCM4057
SCM4058
SCM4059
SCM4060
SCM4061
SCM4062
SCM4063
SCM4064
SCM4065
SCM4066
SCM4067
SCM4068
SCM4069
SCM4070
SCM4071
SCM4072
SCM4073
SCM4074
SCM4075
SCM4076
SCM4077
SCM4078
SCM4079
SCM4080
SCM4081
SCM4082
SCM4083
SCM4084
SCM4085
SCM4086
SCM4087
SCM4088
SCM4089
SCM4090
SCM4091
SCM4092
SCM4093
SCM4094
SCM4095
SCM4096
SCM4097
SCM4098
SCM4099
SCM4100
SCM4101
SCM4102
SCM4103
SCM4104
SCM4105
SCM4106
SCM4107
SCM4108
SCM4109
SCM4110
SCM4111
SCM4112
SCM4113
SCM4114
SCM4115
SCM4116
SCM4117
SCM4118
SCM4119
SCM4120
SCM4121
SCM4122
SCM4123
SCM4124
SCM4125
SCM4126
SCM4127
SCM4128
SCM4129
SCM4130
SCM4131
SCM4132
SCM4133
SCM4134
SCM4135
SCM4136
SCM4137
SCM4138
SCM4139
SCM4140
SCM4141
SCM4142
SCM4143
SCM4144
SCM4145
SCM4146
SCM4147
SCM4148
SCM4149
SCM4150
SCM4151
SCM4152
SCM4153
SCM4154
SCM4155
SCM4156
SCM4157
SCM4158
SCM4159
SCM4160
SCM4161
SCM4162
SCM4163
SCM4164
SCM4165
SCM4166
SCM4167
SCM4168
SCM4169
SCM4170
SCM4171
SCM4172
SCM4173
SCM4174
SCM4175
SCM4176
SCM4177
SCM4178
SCM4179
SCM4180
SCM4181
SCM4182
SCM4183
SCM4184
SCM4185
SCM4186
SCM4187
SCM4188
SCM4189
SCM4190
SCM4191
SCM4192
SCM4193
SCM4194
SCM4195
SCM4196
SCM4197
SCM4198
SCM4199
SCM4200
SCM4201
SCM4202
SCM4203
SCM4204
SCM4205
SCM4206
SCM4207
SCM4208
SCM4209
SCM4210
SCM4211
SCM4212
SCM4213
SCM4214
SCM4215
SCM4216
SCM4217
SCM4218
SCM4219
SCM4220
SCM4221
SCM4222
SCM4223
SCM4224
SCM4225
SCM4226
SCM4227
SCM4228
SCM4229
SCM4230
SCM4231
SCM4232
SCM4233
SCM4234
SCM4235
SCM4236
SCM4237
SCM4238
SCM4239
SCM4240
SCM4241
SCM4242
SCM4243
SCM4244
SCM4245
SCM4246
SCM4247
SCM4248
SCM4249
SCM4250
SCM4251
SCM4252
SCM4253
SCM4254
SCM4255
SCM4256
SCM4257
SCM4258
SCM4259
SCM4260
SCM4261
SCM4262
SCM4263
SCM4264
SCM4265
SCM4266
SCM4267
SCM4268
SCM4269
SCM4270
SCM4271
SCM4272
SCM4273
SCM4274
SCM4275
SCM4276
SCM4277
SCM4278
SCM4279
SCM4280
SCM4281
SCM4282
SCM4283
SCM4284
SCM4285
SCM4286
SCM4287
SCM4288
SCM4289
SCM4290
SCM4291
SCM4292
SCM4293
SCM4294
SCM4295
SCM4296
SCM4297
SCM4298
SCM4299
SCM4300
SCM4301
SCM4302
SCM4303
SCM4304
SCM4305
SCM4306
SCM4307
SCM4308
SCM4309
SCM4310
SCM4311
SCM4312
SCM4313
SCM4314
SCM4315
SCM4316
SCM4317
SCM4318
SCM4319
SCM4320
SCM4321
SCM4322
SCM4323
SCM4324
SCM4325
SCM4326
SCM4327
SCM4328
SCM4329
SCM4330
SCM4331
SCM4332
SCM4333
SCM4334
SCM4335
SCM4336
SCM4337
SCM4338
SCM4339
SCM4340
SCM4341
SCM4342
SCM4343
SCM4344
SCM4345
SCM4346
SCM4347
SCM4348
SCM4349
SCM4350
SCM4351
SCM4352
SCM4353
SCM4354
SCM4355
SCM4356
SCM4357
SCM4358
SCM4359
SCM4360
SCM4361
SCM4362
SCM4363
SCM4364
SCM4365
SCM4366
SCM4367
SCM4368
SCM4369
SCM4370
SCM4371
SCM4372
SCM4373
SCM4374
SCM4375
SCM4376
SCM4377
SCM4378
SCM4379
SCM4380
SCM4381
SCM4382
SCM4383
SCM4384
SCM4385
SCM4386
SCM4387
SCM4388
SCM4389
SCM4390
SCM4391
SCM4392
SCM4393
SCM4394
SCM4395
SCM4396
SCM4397
SCM4398
SCM4399
SCM4400
SCM4401
SCM4402
SCM4403
SCM4404
SCM4405
SCM4406
SCM4407
SCM4408
SCM4409
SCM4410
SCM4411
SCM4412
SCM4413
SCM4414
SCM4415
SCM4416
SCM4417
SCM4418
SCM4419
SCM4420
SCM4421
SCM4422
SCM4423
SCM4424
SCM4425
SCM4426
SCM4427
SCM4428
SCM4429
SCM4430
SCM4431
SCM4432
SCM4433
SCM4434
SCM4435
SCM4436
SCM4437
SCM4438
SCM4439
SCM4440
SCM4441
SCM4442
SCM4443
SCM4444
SCM4445
SCM4446
SCM4447
SCM4448
SCM4449
SCM4450
SCM4451
SCM4452
SCM4453
SCM4454
SCM4455
SCM4456
SCM4457
SCM4458
SCM4459
SCM4460
SCM4461
SCM4462
SCM4463
SCM4464
SCM4465
SCM4466
SCM4467
SCM4468
SCM4469
SCM4470
SCM4471
SCM4472
SCM4473
SCM4474
SCM4475
SCM4476
SCM4477
SCM4478
SCM4479
SCM4480
SCM4481
SCM4482
SCM4483
SCM4484
SCM4485
SCM4486
SCM4487
SCM4488
SCM4489
SCM4490
SCM4491
SCM4492
SCM4493
SCM4494
SCM4495
SCM4496
SCM4497
SCM4498
SCM4499
SCM4500
SCM4501
SCM4502
SCM4503
SCM4504
SCM4505
SCM4506
SCM4507
SCM4508
SCM4509
SCM4510
SCM4511
SCM4512
SCM4513
SCM4514
SCM4515
SCM4516
SCM4517
SCM4518
SCM4519
SCM4520
SCM4521
SCM4522
SCM4523
SCM4524
SCM4525
SCM4526
SCM4527
SCM4528
SCM4529
SCM4530
SCM4531
SCM4532
SCM4533
SCM4534
SCM4535
SCM4536
SCM4537
SCM4538
SCM4539
SCM4540
SCM4541
SCM4542
SCM4543
SCM4544
SCM4545
SCM4546
SCM4547
SCM4548
SCM4549
SCM4550
SCM4551
SCM4552
SCM4553
SCM4554
SCM4555
SCM4556
SCM4557
SCM4558
SCM4559
SCM4560
SCM4561
SCM4562
SCM4563
SCM4564
SCM4565
SCM4566
SCM4567
SCM4568
SCM4569
SCM4570
SCM4571
SCM4572
SCM4573
SCM4574
SCM4575
SCM4576
SCM4577
SCM4578
SCM4579
SCM4580
SCM4581
SCM4582
SCM4583
SCM4584
SCM4585
SCM4586
SCM4587
SCM4588
SCM4589
SCM4590
SCM4591
SCM4592
SCM4593
SCM4594
SCM4595
SCM4596
SCM4597
SCM4598
SCM4599
SCM4600
SCM4601
SCM4602
SCM4603
SCM4604
SCM4605
SCM4606
SCM4607
SCM4608
SCM4609
SCM4610
SCM4611
SCM4612
SCM4613
SCM4614
SCM4615
SCM4616
SCM4617
SCM4618
SCM4619
SCM4620
SCM4621
SCM4622
SCM4623
SCM4624
SCM4625
SCM4626
SCM4627
SCM4628
SCM4629
SCM4630
SCM4631
SCM4632
SCM4633
SCM4634
SCM4635
SCM4636
SCM4637
SCM4638
SCM4639
SCM4640
SCM4641
SCM4642
SCM4643
SCM4644
SCM4645
SCM4646
SCM4647
SCM4648
SCM4649
SCM4650
SCM4651
SCM4652
SCM4653
SCM4654
SCM4655
SCM4656
SCM4657
SCM4658
SCM4659
SCM4660
SCM4661
SCM4662
SCM4663
SCM4664
SCM4665
SCM4666
SCM4667
SCM4668
SCM4669
SCM4670
SCM4671
SCM4672
SCM4673
SCM4674
SCM4675
SCM4676
SCM4677
SCM4678
SCM4679
SCM4680
SCM4681
SCM4682
SCM4683
SCM4684
SCM4685
SCM4686
SCM4687
SCM4688
SCM4689
SCM4690
SCM4691
SCM4692
SCM4693
SCM4694
SCM4695
SCM4696
SCM4697
SCM4698
SCM4699
SCM4700
SCM4701
SCM4702
SCM4703
SCM4704
SCM4705
SCM4706
SCM4707
SCM4708
SCM4709
SCM4710
SCM4711
SCM4712
SCM4713
SCM4714
SCM4715
SCM4716
SCM4717
SCM4718
SCM4719
SCM4720
SCM4721
SCM4722
SCM4723
SCM4724
SCM4725
SCM4726
SCM4727
SCM4728
SCM4729
SCM4730
SCM4731
SCM4732
SCM4733
SCM4734
SCM4735
SCM4736
SCM4737
SCM4738
SCM4739
SCM4740
SCM4741
SCM4742
SCM4743
SCM4744
SCM4745
SCM4746
SCM4747
SCM4748
SCM4749
SCM4750
SCM4751
SCM4752
SCM4753
SCM4754
SCM4755
SCM4756
SCM4757
SCM4758
SCM4759
SCM4760
SCM4761
SCM4762
SCM4763
SCM4764
SCM4765
SCM4766
SCM4767
SCM4768
SCM4769
SCM4770
SCM4771
SCM4772
SCM4773
SCM4774
SCM4775
SCM4776
SCM4777
SCM4778
SCM4779
SCM4780
SCM4781
SCM4782
SCM4783
SCM4784
SCM4785
SCM4786
SCM4787
SCM4788
SCM4789
SCM4790
SCM4791
SCM4792
SCM4793
SCM4794
SCM4795
SCM4796
SCM4797
SCM4798
SCM4799
SCM4800
SCM4801
SCM4802
SCM4803
SCM4804
SCM4805
SCM4806
SCM4807
SCM4808
SCM4809
SCM4810
SCM4811
SCM4812
SCM4813
SCM4814
SCM4815
SCM4816
SCM4817
SCM4818
SCM4819
SCM4820
SCM4821
SCM4822
SCM4823
SCM4824
SCM4825
SCM4826
SCM4827
SCM4828
SCM4829
SCM4830
SCM4831
SCM4832
SCM4833
SCM4834
SCM4835
SCM4836
SCM4837
SCM4838
SCM4839
SCM4840
SCM4841
SCM4842
SCM4843
SCM4844
SCM4845
SCM4846
SCM4847
SCM4848
SCM4849
SCM4850
SCM4851
SCM4852
SCM4853
SCM4854
SCM4855
SCM4856
SCM4857
SCM4858
SCM4859
SCM4860
SCM4861
SCM4862
SCM4863
SCM4864
SCM4865
SCM4866
SCM4867
SCM4868
SCM4869
SCM4870
SCM4871
SCM4872
SCM4873
SCM4874
SCM4875
SCM4876
SCM4877
SCM4878
SCM4879
SCM4880
SCM4881
SCM4882
SCM4883
SCM4884
SCM4885
SCM4886
SCM4887
SCM4888
SCM4889
SCM4890
SCM4891
SCM4892
SCM4893
SCM4894
SCM4895
SCM4896
SCM4897
SCM4898
SCM4899
SCM4900
SCM4901
SCM4902
SCM4903
SCM4904
SCM4905
SCM4906
SCM4907
SCM4908
SCM4909
SCM4910
SCM4911
SCM4912
SCM4913
SCM4914
SCM4915
SCM4916
SCM4917
SCM4918
SCM4919
SCM4920
SCM4921
SCM4922
SCM4923
SCM4924
SCM4925
SCM4926
SCM4927
SCM4928
SCM4929
SCM4930
SCM4931
SCM4932
SCM4933
SCM4934
SCM4935
SCM4936
SCM4937
SCM4938
SCM4939
SCM4940
SCM4941
SCM4942
SCM4943
SCM4944
SCM4945
SCM4946
SCM4947
SCM4948
SCM4949
SCM4950
SCM4951
SCM4952
SCM4953
SCM4954
SCM4955
SCM4956
SCM4957
SCM4958
SCM4959
SCM4960
SCM4961
SCM4962
SCM4963
SCM4964
SCM4965
SCM4966
SCM4967
SCM4968
SCM4969
SCM4970
SCM4971
SCM4972
SCM4973
SCM4974
SCM4975
SCM4976
SCM4977
SCM4978
SCM4979
SCM4980
SCM4981
SCM4982
SCM4983
SCM4984
SCM4985
SCM4986
SCM4987
SCM4988
SCM4989
SCM4990
SCM4991
SCM4992
SCM4993
SCM4994
SCM4995
SCM4996
SCM4997
SCM4998
SCM4999
SCM5000
SCM5001
SCM5002
SCM5003
SCM5004
SCM5005
SCM5006
SCM5007
SCM5008
SCM5009
SCM5010
SCM5011
SCM5012
SCM5013
SCM5014
SCM5015
SCM5016
SCM5017
SCM5018
SCM5019
SCM5020
SCM5021
SCM5022
SCM5023
SCM5024
SCM5025
SCM5026
SCM5027
SCM5028
SCM5029
SCM5030
SCM5031
SCM5032
SCM5033
SCM5034
SCM5035
SCM5036
SCM5037
SCM5038
Select ORF
YAL001C
YAL002W
YAL004W
YAL005C
YAL008W
YAL009W
YAL010C
YAL011W
YAL012W
YAL013W
YAL014C
YAL015C
YAL016W
YAL017W
YAL018C
YAL019W
YAL020C
YAL021C
YAL022C
YAL023C
YAL024C
YAL025C
YAL026C
YAL027W
YAL028W
YAL029C
YAL030W
YAL031C
YAL032C
YAL033W
YAL034C
YAL035W
YAL036C
YAL037W
YAL038W
YAL039C
YAL040C
YAL041W
YAL042W
YAL043C
YAL044C
YAL046C
YAL047C
YAL048C
YAL049C
YAL051W
YAL053W
YAL054C
YAL055W
YAL056W
YAL058W
YAL059W
YAL060W
YAL061W
YAL062W
YAL065C
YAL067C
YAL069W
YAR002W
YAR003W
YAR007C
YAR008W
YAR014C
YAR015W
YAR018C
YAR019C
YAR023C
YAR027W
YAR028W
YAR030C
YAR031W
YAR033W
YAR035W
YAR042W
YAR044W
YAR047C
YAR050W
YAR053W
YAR071W
YAR073W
YAR075W
YBL001C
YBL002W
YBL003C
YBL004W
YBL005W
YBL007C
YBL008W
YBL009W
YBL010C
YBL011W
YBL012C
YBL013W
YBL014C
YBL015W
YBL016W
YBL017C
YBL018C
YBL020W
YBL021C
YBL022C
YBL023C
YBL027W
YBL028C
YBL029W
YBL030C
YBL031W
YBL032W
YBL033C
YBL034C
YBL035C
YBL036C
YBL038W
YBL039C
YBL040C
YBL041W
YBL042C
YBL043W
YBL044W
YBL045C
YBL046W
YBL047C
YBL049W
YBL050W
YBL051C
YBL052C
YBL054W
YBL055C
YBL056W
YBL057C
YBL058W
YBL060W
YBL061C
YBL062W
YBL063W
YBL064C
YBL065W
YBL066C
YBL067C
YBL068W
YBL069W
YBL070C
YBL071C
YBL073W
YBL074C
YBL075C
YBL076C
YBL077W
YBL079W
YBL080C
YBL081W
YBL082C
YBL083C
YBL084C
YBL085W
YBL086C
YBL088C
YBL089W
YBL090W
YBL091C
YBL092W
YBL093C
YBL095W
YBL096C
YBL097W
YBL098W
YBL099W
YBL101C
YBL103C
YBL104C
YBL105C
YBL107C
YBL111C
YBL113C
YBR001C
YBR003W
YBR004C
YBR005W
YBR006W
YBR007C
YBR008C
YBR009C
YBR010W
YBR011C
YBR017C
YBR018C
YBR019C
YBR020W
YBR021W
YBR022W
YBR023C
YBR024W
YBR025C
YBR026C
YBR027C
YBR028C
YBR029C
YBR033W
YBR034C
YBR035C
YBR036C
YBR037C
YBR038W
YBR039W
YBR040W
YBR041W
YBR042C
YBR043C
YBR045C
YBR046C
YBR047W
YBR048W
YBR049C
YBR050C
YBR053C
YBR054W
YBR055C
YBR056W
YBR057C
YBR058C
YBR059C
YBR061C
YBR063C
YBR064W
YBR065C
YBR068C
YBR069C
YBR070C
YBR071W
YBR072W
YBR073W
YBR074W
YBR075W
YBR076W
YBR078W
YBR079C
YBR080C
YBR081C
YBR082C
YBR083W
YBR084W
YBR085W
YBR086C
YBR087W
YBR088C
YBR089W
YBR090C
YBR091C
YBR092C
YBR093C
YBR094W
YBR095C
YBR097W
YBR098W
YBR100W
YBR101C
YBR102C
YBR103W
YBR104W
YBR105C
YBR106W
YBR108W
YBR109C
YBR110W
YBR111C
YBR112C
YBR114W
YBR115C
YBR116C
YBR117C
YBR118W
YBR119W
YBR120C
YBR121C
YBR122C
YBR123C
YBR124W
YBR126C
YBR127C
YBR129C
YBR130C
YBR131W
YBR132C
YBR133C
YBR134W
YBR136W
YBR137W
YBR139W
YBR140C
YBR141C
YBR142W
YBR143C
YBR145W
YBR146W
YBR147W
YBR148W
YBR150C
YBR151W
YBR153W
YBR154C
YBR155W
YBR158W
YBR159W
YBR160W
YBR162C
YBR163W
YBR164C
YBR165W
YBR166C
YBR167C
YBR168W
YBR169C
YBR170C
YBR171W
YBR172C
YBR173C
YBR175W
YBR178W
YBR179C
YBR180W
YBR181C
YBR182C
YBR183W
YBR185C
YBR186W
YBR187W
YBR188C
YBR189W
YBR191W
YBR192W
YBR193C
YBR194W
YBR195C
YBR196C
YBR197C
YBR198C
YBR199W
YBR200W
YBR202W
YBR203W
YBR204C
YBR205W
YBR209W
YBR210W
YBR212W
YBR213W
YBR215W
YBR216C
YBR218C
YBR219C
YBR220C
YBR221C
YBR222C
YBR223C
YBR224W
YBR225W
YBR228W
YBR229C
YBR231C
YBR233W
YBR235W
YBR236C
YBR237W
YBR238C
YBR239C
YBR240C
YBR241C
YBR242W
YBR243C
YBR245C
YBR246W
YBR247C
YBR248C
YBR249C
YBR250W
YBR251W
YBR252W
YBR253W
YBR255W
YBR256C
YBR257W
YBR258C
YBR259W
YBR261C
YBR262C
YBR263W
YBR267W
YBR268W
YBR269C
YBR270C
YBR271W
YBR273C
YBR275C
YBR278W
YBR279W
YBR280C
YBR281C
YBR282W
YBR283C
YBR284W
YBR286W
YBR287W
YBR288C
YBR289W
YBR290W
YBR291C
YBR293W
YBR294W
YBR295W
YBR297W
YBR298C
YBR299W
YCL002C
YCL006C
YCL008C
YCL009C
YCL010C
YCL011C
YCL012W
YCL014W
YCL016C
YCL017C
YCL018W
YCL019W
YCL020W
YCL022C
YCL024W
YCL025C
YCL027W
YCL028W
YCL029C
YCL030C
YCL031C
YCL032W
YCL034W
YCL035C
YCL036W
YCL037C
YCL038C
YCL039W
YCL040W
YCL042W
YCL043C
YCL044C
YCL045C
YCL047C
YCL048W
YCL049C
YCL050C
YCL051W
YCL052C
YCL055W
YCL056C
YCL057W
YCL058C
YCL059C
YCL061C
YCL063W
YCL064C
YCL073C
YCL074W
YCL075W
YCL076W
YCR002C
YCR003W
YCR005C
YCR007C
YCR008W
YCR009C
YCR010C
YCR011C
YCR012W
YCR013C
YCR014C
YCR015C
YCR016W
YCR017C
YCR018C
YCR019W
YCR020C
YCR021C
YCR022C
YCR023C
YCR024C
YCR025C
YCR026C
YCR028C
YCR030C
YCR031C
YCR032W
YCR033W
YCR035C
YCR036W
YCR037C
YCR041W
YCR042C
YCR043C
YCR044C
YCR045C
YCR046C
YCR047C
YCR048W
YCR051W
YCR052W
YCR053W
YCR054C
YCR057C
YCR059C
YCR060W
YCR061W
YCR062W
YCR063W
YCR064C
YCR065W
YCR066W
YCR067C
YCR068W
YCR069W
YCR071C
YCR072C
YCR073C
YCR075C
YCR076C
YCR077C
YCR079W
YCR081W
YCR082W
YCR084C
YCR086W
YCR087W
YCR088W
YCR089W
YCR090C
YCR091W
YCR092C
YCR093W
YCR094W
YCR095C
YCR097W
YCR098C
YCR099C
YCR100C
YCR101C
YCR102C
YCR105W
YCR106W
YDL001W
YDL002C
YDL003W
YDL004W
YDL005C
YDL006W
YDL007W
YDL008W
YDL010W
YDL011C
YDL013W
YDL014W
YDL015C
YDL017W
YDL019C
YDL020C
YDL021W
YDL022W
YDL023C
YDL024C
YDL025C
YDL027C
YDL028C
YDL029W
YDL030W
YDL031W
YDL033C
YDL034W
YDL036C
YDL037C
YDL038C
YDL040C
YDL041W
YDL042C
YDL043C
YDL044C
YDL045C
YDL046W
YDL047W
YDL048C
YDL049C
YDL050C
YDL051W
YDL052C
YDL053C
YDL054C
YDL055C
YDL056W
YDL057W
YDL058W
YDL059C
YDL060W
YDL062W
YDL063C
YDL064W
YDL065C
YDL066W
YDL067C
YDL069C
YDL070W
YDL072C
YDL073W
YDL074C
YDL076C
YDL077C
YDL079C
YDL080C
YDL082W
YDL083C
YDL084W
YDL085W
YDL086W
YDL088C
YDL089W
YDL090C
YDL091C
YDL092W
YDL093W
YDL094C
YDL095W
YDL096C
YDL097C
YDL099W
YDL100C
YDL101C
YDL102W
YDL103C
YDL104C
YDL105W
YDL106C
YDL108W
YDL109C
YDL110C
YDL111C
YDL113C
YDL114W
YDL115C
YDL116W
YDL117W
YDL118W
YDL119C
YDL121C
YDL122W
YDL123W
YDL124W
YDL126C
YDL127W
YDL128W
YDL129W
YDL131W
YDL132W
YDL133W
YDL134C
YDL135C
YDL136W
YDL137W
YDL138W
YDL139C
YDL140C
YDL141W
YDL142C
YDL143W
YDL144C
YDL145C
YDL146W
YDL147W
YDL148C
YDL149W
YDL150W
YDL151C
YDL153C
YDL154W
YDL155W
YDL156W
YDL158C
YDL159W
YDL160C
YDL162C
YDL164C
YDL165W
YDL166C
YDL167C
YDL168W
YDL169C
YDL170W
YDL171C
YDL173W
YDL174C
YDL175C
YDL176W
YDL178W
YDL180W
YDL182W
YDL183C
YDL185W
YDL186W
YDL188C
YDL189W
YDL190C
YDL191W
YDL192W
YDL193W
YDL194W
YDL195W
YDL196W
YDL197C
YDL198C
YDL199C
YDL200C
YDL201W
YDL202W
YDL203C
YDL204W
YDL205C
YDL206W
YDL207W
YDL208W
YDL209C
YDL210W
YDL211C
YDL212W
YDL213C
YDL214C
YDL215C
YDL216C
YDL217C
YDL218W
YDL220C
YDL221W
YDL223C
YDL224C
YDL226C
YDL227C
YDL230W
YDL231C
YDL232W
YDL233W
YDL234C
YDL236W
YDL237W
YDL238C
YDL239C
YDL240W
YDL242W
YDL248W
YDR003W
YDR004W
YDR008C
YDR009W
YDR010C
YDR011W
YDR014W
YDR015C
YDR016C
YDR017C
YDR018C
YDR019C
YDR020C
YDR021W
YDR022C
YDR023W
YDR025W
YDR026C
YDR028C
YDR030C
YDR032C
YDR033W
YDR034C
YDR035W
YDR036C
YDR037W
YDR038C
YDR040C
YDR041W
YDR044W
YDR046C
YDR047W
YDR048C
YDR049W
YDR050C
YDR051C
YDR052C
YDR053W
YDR054C
YDR056C
YDR057W
YDR058C
YDR059C
YDR060W
YDR061W
YDR062W
YDR063W
YDR064W
YDR065W
YDR066C
YDR067C
YDR068W
YDR069C
YDR071C
YDR072C
YDR073W
YDR074W
YDR075W
YDR076W
YDR079W
YDR080W
YDR081C
YDR082W
YDR083W
YDR084C
YDR085C
YDR087C
YDR088C
YDR089W
YDR090C
YDR091C
YDR092W
YDR093W
YDR094W
YDR096W
YDR097C
YDR099W
YDR101C
YDR102C
YDR103W
YDR104C
YDR105C
YDR107C
YDR108W
YDR109C
YDR110W
YDR111C
YDR113C
YDR114C
YDR116C
YDR117C
YDR118W
YDR119W
YDR120C
YDR122W
YDR123C
YDR124W
YDR125C
YDR126W
YDR127W
YDR128W
YDR129C
YDR130C
YDR131C
YDR132C
YDR135C
YDR136C
YDR137W
YDR138W
YDR139C
YDR140W
YDR142C
YDR143C
YDR144C
YDR145W
YDR146C
YDR147W
YDR148C
YDR150W
YDR151C
YDR152W
YDR153C
YDR157W
YDR158W
YDR159W
YDR160W
YDR161W
YDR162C
YDR163W
YDR164C
YDR165W
YDR166C
YDR167W
YDR168W
YDR169C
YDR170C
YDR171W
YDR172W
YDR173C
YDR175C
YDR176W
YDR177W
YDR178W
YDR179C
YDR180W
YDR181C
YDR182W
YDR183W
YDR184C
YDR185C
YDR186C
YDR187C
YDR188W
YDR189W
YDR190C
YDR191W
YDR192C
YDR194C
YDR195W
YDR196C
YDR197W
YDR198C
YDR200C
YDR202C
YDR203W
YDR204W
YDR205W
YDR206W
YDR207C
YDR208W
YDR209C
YDR211W
YDR212W
YDR213W
YDR214W
YDR216W
YDR217C
YDR218C
YDR219C
YDR221W
YDR222W
YDR223W
YDR224C
YDR225W
YDR226W
YDR227W
YDR228C
YDR229W
YDR230W
YDR231C
YDR232W
YDR233C
YDR234W
YDR235W
YDR237W
YDR238C
YDR239C
YDR240C
YDR241W
YDR242W
YDR243C
YDR244W
YDR245W
YDR247W
YDR248C
YDR249C
YDR251W
YDR252W
YDR253C
YDR254W
YDR255C
YDR256C
YDR257C
YDR258C
YDR259C
YDR261C
YDR262W
YDR263C
YDR264C
YDR265W
YDR266C
YDR267C
YDR268W
YDR270W
YDR272W
YDR273W
YDR274C
YDR275W
YDR276C
YDR277C
YDR279W
YDR280W
YDR282C
YDR283C
YDR284C
YDR285W
YDR286C
YDR287W
YDR288W
YDR291W
YDR292C
YDR293C
YDR294C
YDR295C
YDR297W
YDR298C
YDR299W
YDR300C
YDR301W
YDR302W
YDR303C
YDR304C
YDR305C
YDR306C
YDR307W
YDR310C
YDR311W
YDR312W
YDR313C
YDR314C
YDR315C
YDR316W
YDR317W
YDR318W
YDR319C
YDR320C
YDR321W
YDR322W
YDR323C
YDR324C
YDR325W
YDR326C
YDR327W
YDR328C
YDR329C
YDR330W
YDR331W
YDR332W
YDR333C
YDR335W
YDR336W
YDR337W
YDR338C
YDR339C
YDR341C
YDR345C
YDR346C
YDR347W
YDR348C
YDR349C
YDR350C
YDR351W
YDR352W
YDR353W
YDR354W
YDR356W
YDR357C
YDR358W
YDR359C
YDR361C
YDR362C
YDR363W
YDR364C
YDR365C
YDR366C
YDR367W
YDR368W
YDR369C
YDR370C
YDR371W
YDR372C
YDR374C
YDR375C
YDR376W
YDR379W
YDR380W
YDR381W
YDR383C
YDR384C
YDR387C
YDR388W
YDR389W
YDR390C
YDR391C
YDR392W
YDR393W
YDR394W
YDR395W
YDR396W
YDR397C
YDR398W
YDR399W
YDR400W
YDR401W
YDR402C
YDR403W
YDR405W
YDR406W
YDR408C
YDR409W
YDR410C
YDR411C
YDR414C
YDR415C
YDR416W
YDR417C
YDR418W
YDR419W
YDR420W
YDR421W
YDR422C
YDR423C
YDR425W
YDR426C
YDR427W
YDR428C
YDR429C
YDR430C
YDR432W
YDR433W
YDR434W
YDR435C
YDR436W
YDR437W
YDR438W
YDR439W
YDR441C
YDR442W
YDR443C
YDR444W
YDR445C
YDR446W
YDR447C
YDR449C
YDR450W
YDR451C
YDR452W
YDR453C
YDR455C
YDR456W
YDR457W
YDR458C
YDR459C
YDR460W
YDR462W
YDR463W
YDR464W
YDR466W
YDR467C
YDR468C
YDR469W
YDR470C
YDR471W
YDR474C
YDR475C
YDR476C
YDR477W
YDR478W
YDR479C
YDR480W
YDR481C
YDR482C
YDR483W
YDR484W
YDR485C
YDR486C
YDR487C
YDR488C
YDR489W
YDR490C
YDR492W
YDR493W
YDR494W
YDR495C
YDR496C
YDR497C
YDR498C
YDR499W
YDR500C
YDR502C
YDR503C
YDR504C
YDR505C
YDR506C
YDR507C
YDR508C
YDR510W
YDR512C
YDR513W
YDR514C
YDR515W
YDR516C
YDR517W
YDR518W
YDR519W
YDR520C
YDR521W
YDR522C
YDR523C
YDR524C
YDR526C
YDR527W
YDR528W
YDR529C
YDR530C
YDR531W
YDR533C
YDR534C
YDR535C
YDR536W
YDR537C
YDR538W
YDR539W
YDR540C
YDR541C
YEL002C
YEL004W
YEL006W
YEL007W
YEL008W
YEL009C
YEL010W
YEL011W
YEL012W
YEL013W
YEL014C
YEL015W
YEL016C
YEL017W
YEL018W
YEL019C
YEL020C
YEL021W
YEL023C
YEL024W
YEL025C
YEL028W
YEL029C
YEL030W
YEL031W
YEL032W
YEL036C
YEL037C
YEL038W
YEL040W
YEL041W
YEL042W
YEL043W
YEL044W
YEL046C
YEL047C
YEL049W
YEL050C
YEL051W
YEL052W
YEL053C
YEL054C
YEL055C
YEL056W
YEL057C
YEL058W
YEL060C
YEL061C
YEL062W
YEL063C
YEL064C
YEL067C
YEL068C
YEL069C
YEL070W
YEL071W
YEL072W
YEL073C
YEL077C
YER001W
YER002W
YER003C
YER004W
YER006W
YER007W
YER008C
YER009W
YER010C
YER011W
YER012W
YER013W
YER014W
YER015W
YER016W
YER017C
YER019W
YER020W
YER021W
YER022W
YER023W
YER024W
YER025W
YER027C
YER028C
YER029C
YER031C
YER032W
YER033C
YER034W
YER035W
YER036C
YER037W
YER038C
YER039C
YER040W
YER041W
YER043C
YER044C
YER045C
YER046W
YER047C
YER048C
YER049W
YER050C
YER051W
YER052C
YER053C
YER054C
YER055C
YER056C
YER057C
YER058W
YER059W
YER060W
YER061C
YER062C
YER064C
YER065C
YER066W
YER067W
YER068W
YER070W
YER074W
YER075C
YER076C
YER077C
YER078C
YER079W
YER080W
YER081W
YER082C
YER083C
YER084W
YER085C
YER086W
YER087W
YER088C
YER089C
YER090W
YER091C
YER092W
YER093C
YER094C
YER095W
YER097W
YER098W
YER099C
YER100W
YER101C
YER102W
YER103W
YER104W
YER105C
YER106W
YER107C
YER110C
YER111C
YER113C
YER114C
YER116C
YER118C
YER119C
YER122C
YER123W
YER124C
YER125W
YER126C
YER127W
YER128W
YER129W
YER130C
YER131W
YER132C
YER133W
YER134C
YER135C
YER136W
YER137C
YER139C
YER140W
YER141W
YER142C
YER143W
YER144C
YER145C
YER147C
YER148W
YER149C
YER151C
YER152C
YER153C
YER154W
YER155C
YER156C
YER158C
YER159C
YER161C
YER162C
YER163C
YER164W
YER166W
YER167W
YER168C
YER169W
YER170W
YER171W
YER172C
YER173W
YER175C
YER176W
YER177W
YER178W
YER179W
YER180C
YER181C
YER182W
YER183C
YER184C
YER185W
YER186C
YER187W
YER188W
YFL001W
YFL002C
YFL003C
YFL004W
YFL005W
YFL006W
YFL008W
YFL009W
YFL011W
YFL012W
YFL013C
YFL015C
YFL016C
YFL018C
YFL021W
YFL022C
YFL023W
YFL024C
YFL025C
YFL026W
YFL027C
YFL028C
YFL029C
YFL030W
YFL031W
YFL033C
YFL034W
YFL036W
YFL037W
YFL038C
YFL039C
YFL040W
YFL041W
YFL042C
YFL043C
YFL044C
YFL045C
YFL046W
YFL047W
YFL048C
YFL049W
YFL050C
YFL051C
YFL052W
YFL053W
YFL054C
YFL055W
YFL057C
YFL059W
YFL060C
YFL063W
YFL064C
YFR002W
YFR003C
YFR004W
YFR006W
YFR007W
YFR009W
YFR010W
YFR011C
YFR012W
YFR013W
YFR014C
YFR015C
YFR016C
YFR017C
YFR018C
YFR019W
YFR020W
YFR021W
YFR022W
YFR023W
YFR024C
YFR025C
YFR026C
YFR028C
YFR029W
YFR030W
YFR031C
YFR032C
YFR033C
YFR034C
YFR036W
YFR037C
YFR038W
YFR039C
YFR040W
YFR041C
YFR042W
YFR043C
YFR044C
YFR045W
YFR046C
YFR047C
YFR048W
YFR050C
YFR051C
YFR052W
YFR053C
YFR055W
YFR056C
YFR057W
YGL001C
YGL003C
YGL004C
YGL005C
YGL006W
YGL007W
YGL008C
YGL009C
YGL010W
YGL011C
YGL012W
YGL013C
YGL014W
YGL015C
YGL017W
YGL018C
YGL019W
YGL020C
YGL021W
YGL022W
YGL024W
YGL025C
YGL026C
YGL027C
YGL030W
YGL031C
YGL033W
YGL035C
YGL037C
YGL038C
YGL039W
YGL040C
YGL041C
YGL042C
YGL043W
YGL044C
YGL045W
YGL046W
YGL047W
YGL048C
YGL049C
YGL050W
YGL051W
YGL052W
YGL053W
YGL055W
YGL057C
YGL058W
YGL059W
YGL060W
YGL061C
YGL062W
YGL063W
YGL064C
YGL065C
YGL066W
YGL067W
YGL068W
YGL069C
YGL070C
YGL071W
YGL072C
YGL073W
YGL074C
YGL076C
YGL077C
YGL078C
YGL079W
YGL080W
YGL081W
YGL082W
YGL083W
YGL084C
YGL085W
YGL086W
YGL087C
YGL088W
YGL089C
YGL090W
YGL091C
YGL092W
YGL094C
YGL095C
YGL096W
YGL097W
YGL098W
YGL099W
YGL100W
YGL101W
YGL102C
YGL103W
YGL104C
YGL105W
YGL107C
YGL108C
YGL110C
YGL111W
YGL112C
YGL113W
YGL114W
YGL115W
YGL116W
YGL117W
YGL119W
YGL120C
YGL121C
YGL122C
YGL123W
YGL124C
YGL125W
YGL126W
YGL127C
YGL128C
YGL129C
YGL130W
YGL131C
YGL132W
YGL133W
YGL134W
YGL136C
YGL137W
YGL138C
YGL139W
YGL140C
YGL141W
YGL142C
YGL143C
YGL144C
YGL145W
YGL146C
YGL147C
YGL148W
YGL149W
YGL150C
YGL151W
YGL152C
YGL153W
YGL154C
YGL155W
YGL156W
YGL157W
YGL158W
YGL159W
YGL160W
YGL161C
YGL162W
YGL163C
YGL164C
YGL165C
YGL166W
YGL167C
YGL169W
YGL170C
YGL171W
YGL172W
YGL173C
YGL174W
YGL175C
YGL176C
YGL177W
YGL178W
YGL179C
YGL180W
YGL181W
YGL182C
YGL183C
YGL184C
YGL185C
YGL186C
YGL187C
YGL188C
YGL189C
YGL190C
YGL192W
YGL193C
YGL194C
YGL195W
YGL196W
YGL197W
YGL200C
YGL201C
YGL202W
YGL203C
YGL205W
YGL206C
YGL207W
YGL208W
YGL209W
YGL210W
YGL211W
YGL212W
YGL213C
YGL214W
YGL215W
YGL216W
YGL218W
YGL219C
YGL220W
YGL221C
YGL223C
YGL224C
YGL225W
YGL226W
YGL227W
YGL228W
YGL229C
YGL232W
YGL233W
YGL235W
YGL236C
YGL237C
YGL238W
YGL239C
YGL240W
YGL242C
YGL244W
YGL245W
YGL247W
YGL248W
YGL249W
YGL250W
YGL251C
YGL252C
YGL253W
YGL254W
YGL255W
YGL256W
YGL259W
YGL261C
YGL262W
YGL263W
YGR001C
YGR002C
YGR003W
YGR004W
YGR005C
YGR006W
YGR007W
YGR008C
YGR009C
YGR010W
YGR012W
YGR013W
YGR014W
YGR015C
YGR016W
YGR017W
YGR018C
YGR019W
YGR020C
YGR021W
YGR026W
YGR028W
YGR029W
YGR030C
YGR031W
YGR032W
YGR033C
YGR034W
YGR037C
YGR038W
YGR039W
YGR040W
YGR041W
YGR042W
YGR043C
YGR044C
YGR045C
YGR046W
YGR047C
YGR048W
YGR050C
YGR052W
YGR053C
YGR054W
YGR055W
YGR056W
YGR057C
YGR058W
YGR059W
YGR060W
YGR061C
YGR062C
YGR063C
YGR064W
YGR065C
YGR066C
YGR067C
YGR068C
YGR069W
YGR070W
YGR071C
YGR072W
YGR074W
YGR076C
YGR077C
YGR078C
YGR079W
YGR080W
YGR081C
YGR082W
YGR083C
YGR084C
YGR085C
YGR086C
YGR087C
YGR088W
YGR089W
YGR090W
YGR091W
YGR092W
YGR093W
YGR094W
YGR095C
YGR096W
YGR097W
YGR098C
YGR099W
YGR100W
YGR101W
YGR102C
YGR103W
YGR104C
YGR105W
YGR107W
YGR108W
YGR109C
YGR110W
YGR111W
YGR112W
YGR115C
YGR116W
YGR117C
YGR118W
YGR119C
YGR121C
YGR122W
YGR123C
YGR124W
YGR125W
YGR126W
YGR127W
YGR128C
YGR129W
YGR130C
YGR131W
YGR132C
YGR133W
YGR134W
YGR136W
YGR138C
YGR140W
YGR141W
YGR143W
YGR144W
YGR145W
YGR146C
YGR147C
YGR148C
YGR149W
YGR150C
YGR152C
YGR153W
YGR154C
YGR155W
YGR156W
YGR157W
YGR158C
YGR159C
YGR161C
YGR162W
YGR164W
YGR165W
YGR166W
YGR167W
YGR168C
YGR169C
YGR170W
YGR171C
YGR172C
YGR173W
YGR174C
YGR175C
YGR176W
YGR177C
YGR178C
YGR179C
YGR180C
YGR182C
YGR184C
YGR185C
YGR186W
YGR187C
YGR188C
YGR190C
YGR191W
YGR193C
YGR195W
YGR196C
YGR197C
YGR198W
YGR200C
YGR201C
YGR202C
YGR203W
YGR204W
YGR205W
YGR206W
YGR207C
YGR208W
YGR210C
YGR212W
YGR213C
YGR214W
YGR215W
YGR216C
YGR217W
YGR218W
YGR220C
YGR221C
YGR222W
YGR223C
YGR224W
YGR226C
YGR227W
YGR229C
YGR231C
YGR232W
YGR233C
YGR235C
YGR237C
YGR238C
YGR240C
YGR241C
YGR243W
YGR244C
YGR245C
YGR246C
YGR247W
YGR248W
YGR249W
YGR250C
YGR251W
YGR252W
YGR253C
YGR254W
YGR255C
YGR256W
YGR257C
YGR258C
YGR259C
YGR260W
YGR261C
YGR262C
YGR263C
YGR264C
YGR266W
YGR267C
YGR268C
YGR270W
YGR271W
YGR273C
YGR274C
YGR276C
YGR277C
YGR278W
YGR280C
YGR281W
YGR283C
YGR284C
YGR285C
YGR286C
YGR287C
YGR295C
YHL001W
YHL002W
YHL003C
YHL004W
YHL006C
YHL007C
YHL008C
YHL009C
YHL010C
YHL011C
YHL012W
YHL013C
YHL014C
YHL015W
YHL016C
YHL017W
YHL018W
YHL019C
YHL020C
YHL021C
YHL023C
YHL025W
YHL026C
YHL027W
YHL028W
YHL029C
YHL030W
YHL031C
YHL032C
YHL033C
YHL034C
YHL035C
YHL036W
YHL037C
YHL038C
YHL039W
YHL041W
YHL042W
YHL044W
YHL046C
YHL047C
YHL048W
YHL050C
YHR001W
YHR002W
YHR003C
YHR005C
YHR006W
YHR007C
YHR008C
YHR009C
YHR010W
YHR011W
YHR012W
YHR013C
YHR015W
YHR016C
YHR017W
YHR018C
YHR019C
YHR020W
YHR021C
YHR022C
YHR023W
YHR024C
YHR025W
YHR026W
YHR027C
YHR028C
YHR029C
YHR030C
YHR032W
YHR033W
YHR034C
YHR035W
YHR036W
YHR037W
YHR039C
YHR040W
YHR042W
YHR043C
YHR044C
YHR045W
YHR046C
YHR047C
YHR048W
YHR049W
YHR050W
YHR052W
YHR056C
YHR057C
YHR058C
YHR059W
YHR060W
YHR061C
YHR062C
YHR063C
YHR064C
YHR065C
YHR066W
YHR067W
YHR068W
YHR070W
YHR071W
YHR072W
YHR073W
YHR074W
YHR075C
YHR076W
YHR077C
YHR078W
YHR079C
YHR080C
YHR081W
YHR082C
YHR083W
YHR084W
YHR085W
YHR086W
YHR087W
YHR088W
YHR089C
YHR090C
YHR091C
YHR092C
YHR094C
YHR095W
YHR096C
YHR097C
YHR099W
YHR100C
YHR101C
YHR102W
YHR103W
YHR104W
YHR106W
YHR107C
YHR108W
YHR109W
YHR111W
YHR112C
YHR113W
YHR114W
YHR115C
YHR116W
YHR117W
YHR118C
YHR119W
YHR120W
YHR121W
YHR122W
YHR123W
YHR124W
YHR125W
YHR126C
YHR127W
YHR129C
YHR130C
YHR131C
YHR132C
YHR133C
YHR134W
YHR135C
YHR136C
YHR137W
YHR139C
YHR140W
YHR144C
YHR146W
YHR147C
YHR149C
YHR150W
YHR151C
YHR152W
YHR153C
YHR154W
YHR155W
YHR156C
YHR157W
YHR158C
YHR159W
YHR161C
YHR163W
YHR164C
YHR165C
YHR166C
YHR167W
YHR168W
YHR169W
YHR170W
YHR172W
YHR173C
YHR174W
YHR175W
YHR176W
YHR177W
YHR178W
YHR179W
YHR180W
YHR181W
YHR182W
YHR183W
YHR184W
YHR186C
YHR187W
YHR188C
YHR189W
YHR190W
YHR192W
YHR193C
YHR194W
YHR196W
YHR197W
YHR198C
YHR199C
YHR200W
YHR202W
YHR204W
YHR205W
YHR206W
YHR207C
YHR208W
YHR209W
YHR210C
YHR218W
YIL001W
YIL002C
YIL003W
YIL004C
YIL005W
YIL006W
YIL007C
YIL008W
YIL009W
YIL010W
YIL013C
YIL014W
YIL015W
YIL016W
YIL017C
YIL018W
YIL019W
YIL020C
YIL021W
YIL022W
YIL023C
YIL024C
YIL025C
YIL026C
YIL027C
YIL029C
YIL030C
YIL031W
YIL033C
YIL034C
YIL035C
YIL036W
YIL037C
YIL038C
YIL039W
YIL041W
YIL042C
YIL043C
YIL044C
YIL045W
YIL046W
YIL047C
YIL048W
YIL049W
YIL050W
YIL051C
YIL053W
YIL054W
YIL055C
YIL056W
YIL057C
YIL058W
YIL061C
YIL062C
YIL063C
YIL064W
YIL065C
YIL066C
YIL067C
YIL068C
YIL071C
YIL072W
YIL074C
YIL075C
YIL076W
YIL077C
YIL078W
YIL079C
YIL080W
YIL082W
YIL083C
YIL084C
YIL085C
YIL087C
YIL088C
YIL089W
YIL090W
YIL091C
YIL092W
YIL093C
YIL094C
YIL095W
YIL096C
YIL097W
YIL098C
YIL099W
YIL101C
YIL103W
YIL104C
YIL105C
YIL107C
YIL108W
YIL109C
YIL110W
YIL111W
YIL112W
YIL113W
YIL115C
YIL116W
YIL117C
YIL118W
YIL119C
YIL120W
YIL121W
YIL122W
YIL123W
YIL125W
YIL126W
YIL127C
YIL128W
YIL130W
YIL131C
YIL132C
YIL134W
YIL135C
YIL136W
YIL137C
YIL138C
YIL139C
YIL140W
YIL142W
YIL143C
YIL144W
YIL145C
YIL146C
YIL147C
YIL148W
YIL149C
YIL150C
YIL151C
YIL152W
YIL153W
YIL154C
YIL155C
YIL156W
YIL157C
YIL158W
YIL159W
YIL160C
YIL162W
YIL163C
YIL164C
YIL165C
YIL166C
YIL167W
YIL168W
YIL169C
YIL170W
YIL177C
YIR001C
YIR002C
YIR003W
YIR004W
YIR005W
YIR006C
YIR007W
YIR008C
YIR009W
YIR010W
YIR011C
YIR012W
YIR014W
YIR015W
YIR016W
YIR017C
YIR018W
YIR019C
YIR020C
YIR021W
YIR022W
YIR023W
YIR024C
YIR025W
YIR026C
YIR027C
YIR028W
YIR029W
YIR030C
YIR031C
YIR032C
YIR033W
YIR034C
YIR035C
YIR038C
YIR039C
YIR042C
YJL001W
YJL002C
YJL003W
YJL004C
YJL005W
YJL006C
YJL007C
YJL008C
YJL010C
YJL011C
YJL012C
YJL013C
YJL014W
YJL015C
YJL016W
YJL017W
YJL018W
YJL019W
YJL020C
YJL021C
YJL022W
YJL023C
YJL024C
YJL025W
YJL026W
YJL027C
YJL028W
YJL029C
YJL030W
YJL031C
YJL032W
YJL033W
YJL034W
YJL035C
YJL036W
YJL037W
YJL039C
YJL041W
YJL042W
YJL043W
YJL044C
YJL045W
YJL046W
YJL048C
YJL049W
YJL050W
YJL051W
YJL052W
YJL053W
YJL054W
YJL056C
YJL057C
YJL058C
YJL059W
YJL060W
YJL061W
YJL063C
YJL065C
YJL066C
YJL067W
YJL068C
YJL069C
YJL070C
YJL071W
YJL072C
YJL073W
YJL074C
YJL075C
YJL077C
YJL078C
YJL079C
YJL080C
YJL081C
YJL082W
YJL083W
YJL084C
YJL085W
YJL086C
YJL087C
YJL088W
YJL089W
YJL090C
YJL091C
YJL092W
YJL093C
YJL094C
YJL095W
YJL097W
YJL099W
YJL100W
YJL101C
YJL102W
YJL103C
YJL105W
YJL106W
YJL107C
YJL108C
YJL109C
YJL110C
YJL111W
YJL112W
YJL113W
YJL114W
YJL115W
YJL116C
YJL117W
YJL118W
YJL119C
YJL120W
YJL122W
YJL123C
YJL125C
YJL126W
YJL127C
YJL128C
YJL129C
YJL130C
YJL131C
YJL132W
YJL133W
YJL134W
YJL136C
YJL137C
YJL138C
YJL139C
YJL140W
YJL141C
YJL143W
YJL144W
YJL145W
YJL146W
YJL147C
YJL148W
YJL149W
YJL150W
YJL151C
YJL152W
YJL153C
YJL154C
YJL155C
YJL156C
YJL157C
YJL158C
YJL161W
YJL162C
YJL163C
YJL164C
YJL165C
YJL167W
YJL168C
YJL171C
YJL172W
YJL173C
YJL176C
YJL179W
YJL180C
YJL181W
YJL183W
YJL184W
YJL185C
YJL187C
YJL188C
YJL190C
YJL191W
YJL192C
YJL193W
YJL194W
YJL195C
YJL196C
YJL197W
YJL198W
YJL199C
YJL200C
YJL201W
YJL204C
YJL206C
YJL207C
YJL208C
YJL209W
YJL210W
YJL211C
YJL212C
YJL213W
YJL214W
YJL216C
YJL217W
YJL218W
YJL225C
YJR001W
YJR002W
YJR003C
YJR004C
YJR005W
YJR006W
YJR007W
YJR008W
YJR009C
YJR010W
YJR011C
YJR012C
YJR013W
YJR014W
YJR015W
YJR016C
YJR017C
YJR018W
YJR019C
YJR020W
YJR021C
YJR024C
YJR025C
YJR030C
YJR031C
YJR032W
YJR033C
YJR034W
YJR035W
YJR036C
YJR037W
YJR039W
YJR040W
YJR041C
YJR042W
YJR043C
YJR044C
YJR045C
YJR047C
YJR049C
YJR050W
YJR051W
YJR052W
YJR054W
YJR055W
YJR056C
YJR057W
YJR058C
YJR059W
YJR060W
YJR061W
YJR062C
YJR063W
YJR064W
YJR065C
YJR066W
YJR067C
YJR068W
YJR069C
YJR070C
YJR072C
YJR073C
YJR074W
YJR075W
YJR076C
YJR077C
YJR078W
YJR080C
YJR082C
YJR083C
YJR084W
YJR086W
YJR087W
YJR088C
YJR090C
YJR091C
YJR092W
YJR093C
YJR094C
YJR096W
YJR097W
YJR098C
YJR100C
YJR101W
YJR102C
YJR103W
YJR104C
YJR105W
YJR106W
YJR107W
YJR109C
YJR110W
YJR111C
YJR112W
YJR113C
YJR115W
YJR116W
YJR117W
YJR118C
YJR119C
YJR120W
YJR121W
YJR122W
YJR124C
YJR126C
YJR127C
YJR128W
YJR129C
YJR130C
YJR131W
YJR132W
YJR134C
YJR135C
YJR137C
YJR138W
YJR139C
YJR140C
YJR142W
YJR143C
YJR144W
YJR145C
YJR146W
YJR148W
YJR149W
YJR150C
YJR151C
YJR152W
YJR153W
YJR154W
YJR161C
YKL002W
YKL003C
YKL004W
YKL005C
YKL006W
YKL007W
YKL009W
YKL010C
YKL011C
YKL012W
YKL014C
YKL015W
YKL016C
YKL017C
YKL018W
YKL019W
YKL020C
YKL021C
YKL022C
YKL023W
YKL024C
YKL025C
YKL026C
YKL027W
YKL028W
YKL030W
YKL031W
YKL032C
YKL033W
YKL034W
YKL036C
YKL037W
YKL038W
YKL039W
YKL041W
YKL042W
YKL043W
YKL045W
YKL046C
YKL047W
YKL048C
YKL049C
YKL050C
YKL051W
YKL052C
YKL054C
YKL056C
YKL057C
YKL059C
YKL060C
YKL062W
YKL063C
YKL064W
YKL065C
YKL066W
YKL068W
YKL069W
YKL071W
YKL072W
YKL073W
YKL074C
YKL075C
YKL076C
YKL077W
YKL079W
YKL080W
YKL081W
YKL082C
YKL084W
YKL085W
YKL086W
YKL087C
YKL088W
YKL089W
YKL090W
YKL091C
YKL092C
YKL093W
YKL094W
YKL095W
YKL096W
YKL097C
YKL098W
YKL099C
YKL100C
YKL101W
YKL103C
YKL104C
YKL105C
YKL106W
YKL107W
YKL109W
YKL110C
YKL112W
YKL113C
YKL114C
YKL116C
YKL117W
YKL119C
YKL121W
YKL122C
YKL123W
YKL124W
YKL125W
YKL126W
YKL127W
YKL128C
YKL129C
YKL131W
YKL132C
YKL133C
YKL135C
YKL136W
YKL137W
YKL138C
YKL139W
YKL140W
YKL141W
YKL142W
YKL143W
YKL144C
YKL145W
YKL146W
YKL147C
YKL148C
YKL149C
YKL150W
YKL151C
YKL152C
YKL153W
YKL155C
YKL156W
YKL157W
YKL158W
YKL159C
YKL161C
YKL162C
YKL163W
YKL165C
YKL166C
YKL167C
YKL168C
YKL171W
YKL173W
YKL174C
YKL175W
YKL176C
YKL178C
YKL179C
YKL181W
YKL182W
YKL183W
YKL184W
YKL185W
YKL186C
YKL187C
YKL188C
YKL189W
YKL191W
YKL193C
YKL194C
YKL195W
YKL196C
YKL197C
YKL198C
YKL201C
YKL203C
YKL204W
YKL205W
YKL206C
YKL207W
YKL208W
YKL209C
YKL210W
YKL211C
YKL212W
YKL213C
YKL214C
YKL215C
YKL216W
YKL217W
YKL218C
YKL220C
YKL221W
YKL222C
YKR001C
YKR002W
YKR003W
YKR004C
YKR005C
YKR006C
YKR007W
YKR008W
YKR009C
YKR010C
YKR011C
YKR012C
YKR014C
YKR015C
YKR016W
YKR017C
YKR018C
YKR019C
YKR020W
YKR021W
YKR022C
YKR023W
YKR024C
YKR025W
YKR026C
YKR027W
YKR028W
YKR029C
YKR030W
YKR031C
YKR032W
YKR034W
YKR036C
YKR038C
YKR039W
YKR042W
YKR043C
YKR044W
YKR045C
YKR046C
YKR047W
YKR048C
YKR050W
YKR051W
YKR053C
YKR054C
YKR055W
YKR056W
YKR057W
YKR058W
YKR060W
YKR061W
YKR062W
YKR063C
YKR064W
YKR065C
YKR066C
YKR067W
YKR068C
YKR069W
YKR070W
YKR071C
YKR072C
YKR073C
YKR074W
YKR075C
YKR076W
YKR077W
YKR078W
YKR079C
YKR080W
YKR082W
YKR083C
YKR084C
YKR085C
YKR086W
YKR087C
YKR088C
YKR089C
YKR090W
YKR092C
YKR093W
YKR094C
YKR095W
YKR096W
YKR097W
YKR098C
YKR099W
YKR100C
YKR101W
YKR102W
YKR103W
YKR104W
YKR105C
YKR106W
YLL001W
YLL002W
YLL003W
YLL004W
YLL005C
YLL006W
YLL007C
YLL008W
YLL010C
YLL011W
YLL012W
YLL013C
YLL014W
YLL016W
YLL018C
YLL019C
YLL020C
YLL021W
YLL022C
YLL023C
YLL024C
YLL026W
YLL029W
YLL031C
YLL032C
YLL033W
YLL034C
YLL035W
YLL036C
YLL039C
YLL040C
YLL041C
YLL043W
YLL044W
YLL045C
YLL046C
YLL048C
YLL049W
YLL050C
YLL051C
YLL053C
YLL054C
YLL055W
YLL056C
YLL057C
YLL059C
YLL060C
YLL062C
YLL063C
YLL066C
YLL067C
YLR001C
YLR002C
YLR003C
YLR004C
YLR005W
YLR006C
YLR007W
YLR008C
YLR009W
YLR011W
YLR012C
YLR014C
YLR015W
YLR016C
YLR017W
YLR018C
YLR019W
YLR020C
YLR021W
YLR022C
YLR023C
YLR024C
YLR025W
YLR026C
YLR027C
YLR028C
YLR029C
YLR030W
YLR031W
YLR032W
YLR035C
YLR036C
YLR037C
YLR038C
YLR039C
YLR041W
YLR042C
YLR044C
YLR045C
YLR047C
YLR049C
YLR050C
YLR051C
YLR054C
YLR055C
YLR056W
YLR057W
YLR058C
YLR059C
YLR060W
YLR061W
YLR062C
YLR063W
YLR064W
YLR066W
YLR067C
YLR068W
YLR069C
YLR070C
YLR071C
YLR072W
YLR074C
YLR075W
YLR077W
YLR078C
YLR079W
YLR080W
YLR081W
YLR083C
YLR084C
YLR085C
YLR086W
YLR087C
YLR088W
YLR089C
YLR090W
YLR091W
YLR092W
YLR095C
YLR096W
YLR097C
YLR098C
YLR099C
YLR100W
YLR101C
YLR102C
YLR103C
YLR104W
YLR105C
YLR106C
YLR107W
YLR108C
YLR109W
YLR113W
YLR114C
YLR115W
YLR116W
YLR117C
YLR118C
YLR119W
YLR120C
YLR121C
YLR125W
YLR126C
YLR127C
YLR128W
YLR129W
YLR130C
YLR131C
YLR132C
YLR133W
YLR134W
YLR135W
YLR136C
YLR137W
YLR138W
YLR139C
YLR141W
YLR142W
YLR143W
YLR144C
YLR145W
YLR146C
YLR147C
YLR148W
YLR149C
YLR150W
YLR151C
YLR152C
YLR153C
YLR154C
YLR162W
YLR163C
YLR164W
YLR166C
YLR167W
YLR168C
YLR171W
YLR172C
YLR173W
YLR174W
YLR175W
YLR176C
YLR177W
YLR178C
YLR180W
YLR181C
YLR182W
YLR183C
YLR185W
YLR186W
YLR187W
YLR188W
YLR190W
YLR191W
YLR193C
YLR194C
YLR195C
YLR196W
YLR197W
YLR199C
YLR201C
YLR203C
YLR204W
YLR205C
YLR207W
YLR208W
YLR209C
YLR210W
YLR211C
YLR212C
YLR214W
YLR215C
YLR216C
YLR217W
YLR219W
YLR220W
YLR221C
YLR222C
YLR223C
YLR224W
YLR225C
YLR227C
YLR228C
YLR229C
YLR230W
YLR231C
YLR233C
YLR234W
YLR237W
YLR238W
YLR239C
YLR240W
YLR241W
YLR242C
YLR243W
YLR244C
YLR247C
YLR248W
YLR249W
YLR250W
YLR251W
YLR253W
YLR254C
YLR256W
YLR257W
YLR258W
YLR259C
YLR260W
YLR262C
YLR263W
YLR266C
YLR268W
YLR269C
YLR270W
YLR271W
YLR273C
YLR274W
YLR275W
YLR276C
YLR277C
YLR278C
YLR281C
YLR283W
YLR285W
YLR286C
YLR287C
YLR288C
YLR289W
YLR290C
YLR291C
YLR292C
YLR294C
YLR296W
YLR297W
YLR298C
YLR299W
YLR300W
YLR301W
YLR302C
YLR303W
YLR304C
YLR305C
YLR306W
YLR307W
YLR308W
YLR309C
YLR310C
YLR312C
YLR313C
YLR314C
YLR315W
YLR316C
YLR317W
YLR318W
YLR319C
YLR320W
YLR321C
YLR322W
YLR323C
YLR324W
YLR325C
YLR326W
YLR328W
YLR329W
YLR333C
YLR334C
YLR335W
YLR336C
YLR337C
YLR340W
YLR341W
YLR342W
YLR343W
YLR344W
YLR345W
YLR346C
YLR347C
YLR348C
YLR349W
YLR351C
YLR352W
YLR353W
YLR354C
YLR355C
YLR356W
YLR357W
YLR358C
YLR359W
YLR360W
YLR361C
YLR362W
YLR363C
YLR364W
YLR365W
YLR366W
YLR367W
YLR368W
YLR369W
YLR370C
YLR373C
YLR374C
YLR375W
YLR376C
YLR377C
YLR378C
YLR379W
YLR381W
YLR382C
YLR383W
YLR384C
YLR385C
YLR386W
YLR387C
YLR388W
YLR389C
YLR392C
YLR393W
YLR394W
YLR396C
YLR397C
YLR398C
YLR399C
YLR400W
YLR401C
YLR403W
YLR404W
YLR405W
YLR408C
YLR409C
YLR410W
YLR411W
YLR412W
YLR413W
YLR414C
YLR416C
YLR417W
YLR418C
YLR419W
YLR420W
YLR421C
YLR422W
YLR423C
YLR424W
YLR426W
YLR427W
YLR429W
YLR430W
YLR431C
YLR432W
YLR433C
YLR436C
YLR437C
YLR438W
YLR439W
YLR440C
YLR441C
YLR442C
YLR443W
YLR445W
YLR446W
YLR447C
YLR448W
YLR449W
YLR450W
YLR451W
YLR452C
YLR453C
YLR454W
YLR455W
YLR456W
YLR457C
YLR458W
YLR459W
YLR460C
YLR462W
YLR464W
YLR465C
YML002W
YML003W
YML004C
YML005W
YML007W
YML008C
YML009C
YML010W
YML011C
YML012W
YML013W
YML014W
YML015C
YML016C
YML017W
YML018C
YML020W
YML021C
YML022W
YML023C
YML025C
YML026C
YML027W
YML028W
YML029W
YML030W
YML031W
YML032C
YML033W
YML034W
YML035C
YML036W
YML037C
YML041C
YML042W
YML043C
YML046W
YML047C
YML048W
YML049C
YML050W
YML051W
YML052W
YML053C
YML054C
YML055W
YML056C
YML057W
YML059C
YML060W
YML061C
YML062C
YML063W
YML064C
YML065W
YML066C
YML067C
YML068W
YML069W
YML070W
YML071C
YML072C
YML073C
YML075C
YML076C
YML077W
YML078W
YML079W
YML080W
YML081W
YML082W
YML083C
YML084W
YML085C
YML086C
YML087C
YML088W
YML089C
YML090W
YML091C
YML092C
YML093W
YML095C
YML096W
YML097C
YML099C
YML100W
YML101C
YML102W
YML103C
YML104C
YML105C
YML106W
YML107C
YML109W
YML112W
YML113W
YML114C
YML115C
YML116W
YML117W
YML118W
YML119W
YML120C
YML121W
YML122C
YML123C
YML124C
YML125C
YML126C
YML127W
YML128C
YML130C
YML131W
YML133C
YMR001C
YMR002W
YMR003W
YMR004W
YMR005W
YMR007W
YMR009W
YMR010W
YMR011W
YMR012W
YMR013C
YMR014W
YMR015C
YMR016C
YMR017W
YMR018W
YMR019W
YMR020W
YMR022W
YMR023C
YMR025W
YMR026C
YMR027W
YMR028W
YMR029C
YMR030W
YMR031C
YMR033W
YMR034C
YMR035W
YMR036C
YMR037C
YMR039C
YMR040W
YMR041C
YMR042W
YMR043W
YMR044W
YMR045C
YMR046C
YMR047C
YMR048W
YMR049C
YMR052W
YMR053C
YMR054W
YMR055C
YMR056C
YMR058W
YMR060C
YMR061W
YMR062C
YMR063W
YMR064W
YMR065W
YMR066W
YMR067C
YMR069W
YMR070W
YMR071C
YMR072W
YMR073C
YMR074C
YMR075W
YMR076C
YMR077C
YMR078C
YMR079W
YMR080C
YMR081C
YMR083W
YMR084W
YMR085W
YMR086W
YMR087W
YMR088C
YMR089C
YMR090W
YMR091C
YMR092C
YMR093W
YMR094W
YMR095C
YMR096W
YMR097C
YMR098C
YMR100W
YMR102C
YMR103C
YMR104C
YMR105C
YMR107W
YMR108W
YMR109W
YMR110C
YMR111C
YMR113W
YMR114C
YMR115W
YMR116C
YMR118C
YMR119W
YMR120C
YMR121C
YMR124W
YMR125W
YMR126C
YMR127C
YMR128W
YMR129W
YMR130W
YMR131C
YMR132C
YMR133W
YMR134W
YMR135C
YMR139W
YMR140W
YMR142C
YMR143W
YMR144W
YMR145C
YMR146C
YMR147W
YMR149W
YMR150C
YMR154C
YMR155W
YMR156C
YMR157C
YMR158W
YMR159C
YMR160W
YMR161W
YMR162C
YMR163C
YMR164C
YMR165C
YMR166C
YMR167W
YMR168C
YMR169C
YMR170C
YMR171C
YMR173W
YMR175W
YMR176W
YMR177W
YMR178W
YMR179W
YMR183C
YMR184W
YMR185W
YMR186W
YMR187C
YMR188C
YMR189W
YMR190C
YMR191W
YMR192W
YMR194W
YMR195W
YMR196W
YMR197C
YMR198W
YMR199W
YMR200W
YMR201C
YMR202W
YMR203W
YMR204C
YMR205C
YMR206W
YMR207C
YMR208W
YMR209C
YMR210W
YMR211W
YMR212C
YMR213W
YMR214W
YMR215W
YMR216C
YMR217W
YMR218C
YMR219W
YMR220W
YMR221C
YMR223W
YMR224C
YMR225C
YMR226C
YMR227C
YMR228W
YMR229C
YMR231W
YMR232W
YMR233W
YMR234W
YMR235C
YMR237W
YMR238W
YMR239C
YMR240C
YMR241W
YMR242C
YMR243C
YMR244W
YMR245W
YMR246W
YMR247C
YMR250W
YMR251W
YMR253C
YMR257C
YMR258C
YMR259C
YMR261C
YMR262W
YMR263W
YMR264W
YMR265C
YMR266W
YMR267W
YMR268C
YMR270C
YMR271C
YMR272C
YMR273C
YMR275C
YMR276W
YMR277W
YMR278W
YMR279C
YMR280C
YMR282C
YMR283C
YMR285C
YMR287C
YMR288W
YMR289W
YMR290C
YMR291W
YMR293C
YMR294W
YMR295C
YMR296C
YMR297W
YMR298W
YMR299C
YMR300C
YMR301C
YMR302C
YMR303C
YMR304W
YMR306W
YMR307W
YMR308C
YMR309C
YMR310C
YMR311C
YMR312W
YMR313C
YMR314W
YMR315W
YMR316W
YMR317W
YMR318C
YMR319C
YMR320W
YMR322C
YMR323W
YMR324C
YNL001W
YNL002C
YNL003C
YNL004W
YNL005C
YNL006W
YNL007C
YNL008C
YNL009W
YNL010W
YNL011C
YNL012W
YNL014W
YNL016W
YNL017C
YNL018C
YNL019C
YNL020C
YNL021W
YNL022C
YNL023C
YNL024C
YNL025C
YNL026W
YNL027W
YNL029C
YNL030W
YNL031C
YNL032W
YNL033W
YNL034W
YNL035C
YNL036W
YNL037C
YNL038W
YNL039W
YNL040W
YNL041C
YNL043C
YNL045W
YNL046W
YNL047C
YNL048W
YNL050C
YNL051W
YNL052W
YNL053W
YNL054W
YNL055C
YNL056W
YNL058C
YNL059C
YNL061W
YNL062C
YNL063W
YNL064C
YNL065W
YNL066W
YNL067W
YNL068C
YNL069C
YNL070W
YNL071W
YNL072W
YNL073W
YNL076W
YNL077W
YNL078W
YNL080C
YNL082W
YNL083W
YNL084C
YNL085W
YNL087W
YNL088W
YNL091W
YNL092W
YNL093W
YNL094W
YNL095C
YNL096C
YNL097C
YNL098C
YNL099C
YNL100W
YNL101W
YNL102W
YNL103W
YNL104C
YNL105W
YNL106C
YNL107W
YNL108C
YNL109W
YNL110C
YNL112W
YNL113W
YNL114C
YNL115C
YNL116W
YNL117W
YNL119W
YNL120C
YNL121C
YNL122C
YNL123W
YNL124W
YNL125C
YNL126W
YNL127W
YNL128W
YNL129W
YNL132W
YNL133C
YNL134C
YNL136W
YNL137C
YNL138W
YNL139C
YNL140C
YNL141W
YNL142W
YNL144C
YNL145W
YNL146W
YNL148C
YNL149C
YNL150W
YNL151C
YNL152W
YNL154C
YNL155W
YNL156C
YNL157W
YNL158W
YNL159C
YNL160W
YNL161W
YNL163C
YNL164C
YNL165W
YNL167C
YNL168C
YNL169C
YNL172W
YNL174W
YNL175C
YNL176C
YNL177C
YNL179C
YNL181W
YNL182C
YNL183C
YNL187W
YNL188W
YNL189W
YNL191W
YNL192W
YNL193W
YNL194C
YNL195C
YNL196C
YNL197C
YNL199C
YNL200C
YNL201C
YNL202W
YNL203C
YNL204C
YNL205C
YNL206C
YNL207W
YNL208W
YNL209W
YNL210W
YNL211C
YNL212W
YNL213C
YNL214W
YNL215W
YNL216W
YNL217W
YNL218W
YNL219C
YNL220W
YNL221C
YNL222W
YNL223W
YNL224C
YNL225C
YNL226W
YNL227C
YNL228W
YNL229C
YNL233W
YNL234W
YNL236W
YNL237W
YNL238W
YNL239W
YNL241C
YNL242W
YNL243W
YNL244C
YNL245C
YNL246W
YNL247W
YNL248C
YNL249C
YNL250W
YNL251C
YNL253W
YNL254C
YNL256W
YNL257C
YNL258C
YNL259C
YNL260C
YNL261W
YNL262W
YNL263C
YNL265C
YNL266W
YNL267W
YNL268W
YNL270C
YNL271C
YNL272C
YNL273W
YNL274C
YNL275W
YNL276C
YNL277W
YNL278W
YNL279W
YNL280C
YNL282W
YNL283C
YNL286W
YNL287W
YNL288W
YNL289W
YNL290W
YNL291C
YNL292W
YNL293W
YNL294C
YNL295W
YNL297C
YNL298W
YNL299W
YNL300W
YNL301C
YNL302C
YNL303W
YNL305C
YNL306W
YNL307C
YNL308C
YNL309W
YNL310C
YNL311C
YNL312W
YNL313C
YNL314W
YNL315C
YNL316C
YNL318C
YNL319W
YNL320W
YNL321W
YNL323W
YNL324W
YNL325C
YNL326C
YNL327W
YNL329C
YNL330C
YNL336W
YNR001C
YNR002C
YNR003C
YNR004W
YNR005C
YNR006W
YNR007C
YNR009W
YNR010W
YNR011C
YNR012W
YNR013C
YNR014W
YNR015W
YNR016C
YNR017W
YNR018W
YNR019W
YNR020C
YNR021W
YNR022C
YNR023W
YNR026C
YNR027W
YNR028W
YNR029C
YNR030W
YNR031C
YNR032W
YNR033W
YNR034W
YNR036C
YNR038W
YNR039C
YNR040W
YNR041C
YNR043W
YNR044W
YNR045W
YNR046W
YNR047W
YNR048W
YNR049C
YNR050C
YNR051C
YNR052C
YNR053C
YNR054C
YNR055C
YNR056C
YNR057C
YNR058W
YNR059W
YNR060W
YNR061C
YNR063W
YNR064C
YNR065C
YNR066C
YNR067C
YNR068C
YNR069C
YNR070W
YNR071C
YNR072W
YNR073C
YNR074C
YNR075W
YOL001W
YOL002C
YOL003C
YOL004W
YOL006C
YOL007C
YOL008W
YOL009C
YOL010W
YOL012C
YOL013C
YOL015W
YOL016C
YOL017W
YOL018C
YOL019W
YOL020W
YOL021C
YOL022C
YOL023W
YOL024W
YOL025W
YOL027C
YOL028C
YOL029C
YOL030W
YOL031C
YOL032W
YOL033W
YOL034W
YOL036W
YOL037C
YOL038W
YOL040C
YOL041C
YOL042W
YOL043C
YOL044W
YOL045W
YOL046C
YOL047C
YOL049W
YOL051W
YOL052C
YOL053W
YOL054W
YOL056W
YOL057W
YOL058W
YOL059W
YOL060C
YOL062C
YOL063C
YOL064C
YOL065C
YOL066C
YOL067C
YOL068C
YOL069W
YOL070C
YOL071W
YOL072W
YOL073C
YOL075C
YOL076W
YOL077C
YOL078W
YOL080C
YOL081W
YOL082W
YOL083W
YOL084W
YOL086C
YOL087C
YOL090W
YOL092W
YOL093W
YOL094C
YOL095C
YOL096C
YOL098C
YOL099C
YOL100W
YOL102C
YOL103W
YOL104C
YOL105C
YOL106W
YOL107W
YOL108C
YOL110W
YOL111C
YOL112W
YOL113W
YOL114C
YOL115W
YOL116W
YOL117W
YOL118C
YOL119C
YOL120C
YOL121C
YOL122C
YOL123W
YOL124C
YOL125W
YOL126C
YOL128C
YOL129W
YOL130W
YOL131W
YOL132W
YOL133W
YOL134C
YOL135C
YOL136C
YOL137W
YOL138C
YOL139C
YOL140W
YOL141W
YOL143C
YOL145C
YOL146W
YOL147C
YOL148C
YOL149W
YOL151W
YOL152W
YOL153C
YOL156W
YOL157C
YOL158C
YOL159C
YOL163W
YOL164W
YOR001W
YOR002W
YOR003W
YOR004W
YOR005C
YOR006C
YOR007C
YOR008C
YOR010C
YOR011W
YOR012W
YOR013W
YOR014W
YOR017W
YOR018W
YOR019W
YOR021C
YOR022C
YOR025W
YOR026W
YOR027W
YOR028C
YOR029W
YOR030W
YOR031W
YOR032C
YOR033C
YOR034C
YOR035C
YOR036W
YOR037W
YOR038C
YOR039W
YOR040W
YOR041C
YOR042W
YOR043W
YOR044W
YOR046C
YOR047C
YOR048C
YOR049C
YOR051C
YOR052C
YOR054C
YOR055W
YOR056C
YOR057W
YOR058C
YOR059C
YOR060C
YOR061W
YOR062C
YOR063W
YOR064C
YOR065W
YOR066W
YOR067C
YOR068C
YOR069W
YOR070C
YOR071C
YOR073W
YOR074C
YOR075W
YOR076C
YOR077W
YOR078W
YOR079C
YOR080W
YOR081C
YOR084W
YOR085W
YOR086C
YOR087W
YOR088W
YOR089C
YOR091W
YOR092W
YOR093C
YOR095C
YOR096W
YOR097C
YOR098C
YOR099W
YOR100C
YOR102W
YOR103C
YOR104W
YOR106W
YOR107W
YOR108W
YOR109W
YOR110W
YOR111W
YOR112W
YOR113W
YOR114W
YOR116C
YOR117W
YOR118W
YOR119C
YOR120W
YOR121C
YOR123C
YOR124C
YOR125C
YOR126C
YOR127W
YOR128C
YOR129C
YOR131C
YOR132W
YOR134W
YOR135C
YOR136W
YOR137C
YOR138C
YOR140W
YOR141C
YOR142W
YOR143C
YOR145C
YOR146W
YOR147W
YOR148C
YOR149C
YOR151C
YOR153W
YOR154W
YOR155C
YOR156C
YOR157C
YOR158W
YOR159C
YOR160W
YOR161C
YOR162C
YOR164C
YOR165W
YOR166C
YOR168W
YOR171C
YOR172W
YOR173W
YOR174W
YOR175C
YOR176W
YOR177C
YOR178C
YOR179C
YOR181W
YOR184W
YOR186W
YOR188W
YOR189W
YOR190W
YOR191W
YOR192C
YOR193W
YOR195W
YOR196C
YOR197W
YOR198C
YOR199W
YOR201C
YOR202W
YOR204W
YOR205C
YOR206W
YOR207C
YOR208W
YOR209C
YOR210W
YOR211C
YOR212W
YOR213C
YOR214C
YOR215C
YOR216C
YOR217W
YOR219C
YOR220W
YOR222W
YOR223W
YOR224C
YOR227W
YOR228C
YOR229W
YOR230W
YOR231W
YOR232W
YOR233W
YOR234C
YOR236W
YOR237W
YOR238W
YOR239W
YOR240W
YOR241W
YOR242C
YOR243C
YOR244W
YOR245C
YOR246C
YOR249C
YOR250C
YOR251C
YOR252W
YOR253W
YOR254C
YOR255W
YOR256C
YOR257W
YOR259C
YOR260W
YOR261C
YOR262W
YOR264W
YOR265W
YOR266W
YOR267C
YOR268C
YOR269W
YOR270C
YOR271C
YOR272W
YOR273C
YOR274W
YOR275C
YOR276W
YOR278W
YOR279C
YOR280C
YOR281C
YOR282W
YOR283W
YOR284W
YOR285W
YOR286W
YOR287C
YOR288C
YOR289W
YOR290C
YOR291W
YOR292C
YOR293W
YOR295W
YOR296W
YOR298W
YOR299W
YOR301W
YOR303W
YOR304W
YOR306C
YOR307C
YOR311C
YOR312C
YOR313C
YOR315W
YOR316C
YOR317W
YOR318C
YOR319W
YOR320C
YOR321W
YOR322C
YOR323C
YOR324C
YOR325W
YOR326W
YOR327C
YOR328W
YOR329C
YOR330C
YOR331C
YOR332W
YOR334W
YOR335C
YOR336W
YOR337W
YOR338W
YOR340C
YOR341W
YOR342C
YOR343C
YOR344C
YOR345C
YOR346W
YOR347C
YOR348C
YOR349W
YOR350C
YOR351C
YOR352W
YOR353C
YOR354C
YOR355W
YOR356W
YOR357C
YOR358W
YOR359W
YOR360C
YOR361C
YOR362C
YOR363C
YOR364W
YOR365C
YOR367W
YOR368W
YOR369C
YOR370C
YOR371C
YOR373W
YOR375C
YOR376W
YOR377W
YOR378W
YOR379C
YOR380W
YOR381W
YOR384W
YOR385W
YOR386W
YOR389W
YOR390W
YOR391C
YPL001W
YPL002C
YPL003W
YPL004C
YPL005W
YPL007C
YPL008W
YPL009C
YPL010W
YPL011C
YPL012W
YPL013C
YPL014W
YPL015C
YPL016W
YPL017C
YPL018W
YPL019C
YPL021W
YPL022W
YPL023C
YPL024W
YPL026C
YPL027W
YPL028W
YPL029W
YPL030W
YPL031C
YPL032C
YPL033C
YPL034W
YPL036W
YPL040C
YPL041C
YPL042C
YPL043W
YPL044C
YPL045W
YPL046C
YPL047W
YPL048W
YPL049C
YPL050C
YPL052W
YPL053C
YPL054W
YPL055C
YPL057C
YPL058C
YPL060W
YPL061W
YPL063W
YPL064C
YPL065W
YPL066W
YPL067C
YPL068C
YPL069C
YPL070W
YPL071C
YPL072W
YPL073C
YPL075W
YPL076W
YPL077C
YPL078C
YPL079W
YPL080C
YPL082C
YPL083C
YPL084W
YPL085W
YPL087W
YPL088W
YPL089C
YPL090C
YPL091W
YPL092W
YPL093W
YPL094C
YPL095C
YPL096W
YPL097W
YPL099C
YPL100W
YPL101W
YPL103C
YPL104W
YPL105C
YPL106C
YPL107W
YPL108W
YPL109C
YPL110C
YPL111W
YPL112C
YPL113C
YPL114W
YPL115C
YPL116W
YPL117C
YPL118W
YPL119C
YPL120W
YPL121C
YPL122C
YPL124W
YPL125W
YPL127C
YPL128C
YPL129W
YPL131W
YPL132W
YPL133C
YPL134C
YPL137C
YPL138C
YPL139C
YPL140C
YPL141C
YPL142C
YPL143W
YPL145C
YPL146C
YPL147W
YPL148C
YPL149W
YPL150W
YPL151C
YPL152W
YPL153C
YPL154C
YPL155C
YPL156C
YPL157W
YPL158C
YPL160W
YPL161C
YPL162C
YPL164C
YPL165C
YPL166W
YPL167C
YPL168W
YPL169C
YPL170W
YPL171C
YPL172C
YPL173W
YPL174C
YPL175W
YPL176C
YPL178W
YPL179W
YPL180W
YPL181W
YPL183C
YPL184C
YPL185W
YPL186C
YPL188W
YPL189W
YPL190C
YPL191C
YPL194W
YPL195W
YPL196W
YPL199C
YPL201C
YPL202C
YPL203W
YPL204W
YPL206C
YPL207W
YPL208W
YPL209C
YPL210C
YPL211W
YPL212C
YPL214C
YPL215W
YPL216W
YPL217C
YPL218W
YPL219W
YPL222W
YPL223C
YPL224C
YPL226W
YPL227C
YPL228W
YPL229W
YPL230W
YPL231W
YPL232W
YPL233W
YPL234C
YPL235W
YPL236C
YPL237W
YPL238C
YPL239W
YPL240C
YPL242C
YPL243W
YPL244C
YPL245W
YPL246C
YPL247C
YPL248C
YPL249C
YPL250C
YPL253C
YPL254W
YPL255W
YPL256C
YPL259C
YPL260W
YPL262W
YPL263C
YPL265W
YPL266W
YPL267W
YPL268W
YPL269W
YPL270W
YPL271W
YPL272C
YPL275W
YPL277C
YPL279C
YPR001W
YPR002W
YPR003C
YPR004C
YPR005C
YPR006C
YPR008W
YPR009W
YPR011C
YPR013C
YPR015C
YPR017C
YPR018W
YPR019W
YPR021C
YPR022C
YPR023C
YPR024W
YPR025C
YPR026W
YPR029C
YPR031W
YPR032W
YPR033C
YPR034W
YPR035W
YPR036W
YPR037C
YPR038W
YPR039W
YPR040W
YPR041W
YPR042C
YPR043W
YPR044C
YPR045C
YPR046W
YPR047W
YPR049C
YPR050C
YPR051W
YPR054W
YPR055W
YPR056W
YPR057W
YPR060C
YPR061C
YPR062W
YPR064W
YPR065W
YPR068C
YPR069C
YPR071W
YPR073C
YPR074C
YPR075C
YPR077C
YPR078C
YPR079W
YPR081C
YPR082C
YPR083W
YPR084W
YPR085C
YPR086W
YPR087W
YPR089W
YPR090W
YPR091C
YPR092W
YPR093C
YPR094W
YPR095C
YPR097W
YPR101W
YPR102C
YPR103W
YPR104C
YPR105C
YPR106W
YPR107C
YPR108W
YPR109W
YPR110C
YPR111W
YPR112C
YPR113W
YPR114W
YPR115W
YPR116W
YPR117W
YPR118W
YPR119W
YPR120C
YPR122W
YPR124W
YPR125W
YPR127W
YPR128C
YPR129W
YPR131C
YPR132W
YPR133C
YPR135W
YPR136C
YPR137W
YPR138C
YPR139C
YPR140W
YPR141C
YPR142C
YPR143W
YPR144C
YPR145W
YPR146C
YPR147C
YPR148C
YPR149W
YPR150W
YPR151C
YPR152C
YPR153W
YPR154W
YPR155C
YPR156C
YPR157W
YPR158W
YPR159W
YPR160W
YPR161C
YPR162C
YPR163C
YPR164W
YPR165W
YPR166C
YPR167C
YPR168W
YPR169W
YPR170C
YPR171W
YPR172W
YPR173C
YPR174C
YPR175W
YPR176C
YPR177C
YPR178W
YPR179C
YPR180W
YPR181C
YPR183W
YPR184W
YPR185W
YPR186C
YPR187W
YPR188C
YPR189W
YPR190C
YPR191W
YPR193C
YPR194C
YPR196W
YPR197C
YPR198W
YPR199C
YPR200C
YPR201W
Select Gene Name
A1
AAC1
AAC3
AAH1
AAP1'
AAR2
AAT1
AAT2
ABC1
ABD1
ABF1
ABF2
ABP1
ABZ1
ACB1
ACC1
ACE2
ACF2
ACH1
ACO1
ACS1
ACS2
ACT1
ADE1
ADE12
ADE13
ADE16
ADE17
ADE2
ADE3
ADE4
ADE6
ADE8
ADH1
ADH2
ADH3
ADH4
ADH5
ADK1
ADK2
ADP1
ADR1
AEP1
AEP2
AFG1
AFG2
AFG3
AFR1
AGA1
AGP1
AGP2
AGP3
AIP1
AIP2
AKR1
AKR2
ALA1
ALD2
ALD3
ALD6
ALF1
ALG1
ALG11
ALG2
ALG5
ALG6
ALG7
ALG8
ALG9
ALK1
ALO1
ALP1
ALR1
ALR2
AMD1
AMD2
AMS1
ANB1
ANC1
ANP1
AOS1
APA1
APA2
APC1
APC11
APC2
APC4
APC5
APC9
APE2
APE3
APG1
APG13
APG5
APL1
APL2
APL4
APL5
APL6
APM1
APM2
APM3
APM4
APN1
APS2
APS3
APT1
APT2
ARC1
ARC15
ARC18
ARD1
ARE1
ARE2
ARF1
ARF2
ARG1
ARG2
ARG3
ARG4
ARG8
ARG80
ARG81
ARG82
ARH1
ARK1
ARL1
ARO1
ARO2
ARO3
ARO4
ARO7
ARO8
ARO9
ARP1
ARP2
ARP3
ARP4
ARP5
ARP6
ARP7
ARP8
ARP9
ARR1
ARR2
ARR3
ARV1
ASC1
ASE1
ASF1
ASF2
ASH1
ASK10
ASM4
ASN1
ASN2
ASP1
AST1
AST2
ATC1
ATE1
ATF1
ATF2
ATH1
ATM1
ATP1
ATP10
ATP11
ATP12
ATP15
ATP16
ATP2
ATP3
ATP4
ATP5
ATP7
ATR1
ATS1
ATX1
ATX2
AUR1
AUT1
AUT2
AXL1
AYT1
AZF1
BAG7
BAP2
BAP3
BAR1
BAS1
BAT1
BAT2
BBP1
BCK1
BCK2
BCS1
BDF1
BDF2
BEM1
BEM2
BEM3
BEM4
BET1
BET2
BET3
BET4
BET5
BFR1
BFR2
BIG1
BIK1
BIM1
BIO2
BIO3
BIO4
BIO5
BMH1
BMH2
BNA1
BNI1
BNI4
BNR1
BOI1
BOI2
BOS1
BPH1
BPL1
BRF1
BRN1
BRO1
BRR1
BRR2
BSD2
BST1
BTS1
BTT1
BUB1
BUB2
BUB3
BUD2
BUD3
BUD4
BUD6
BUD7
BUD8
BUD9
BUL1
BUR6
CAC2
CAD1
CAF16
CAF17
CAF20
CAF4
CAJ1
CAK1
CAM1
CAN1
CAP1
CAP2
CAR1
CAR2
CAT2
CAT5
CAT8
CBF1
CBF2
CBF5
CBK1
CBP1
CBP2
CBP3
CBP4
CBP6
CBR1
CBS1
CBS2
CBT1
CCA1
CCC1
CCC2
CCE1
CCH1
CCL1
CCP1
CCR4
CCT2
CCT3
CCT4
CCT5
CCT6
CCT7
CCT8
CCZ1
CDA1
CDA2
CDC1
CDC10
CDC11
CDC12
CDC13
CDC14
CDC15
CDC16
CDC19
CDC2
CDC20
CDC21
CDC23
CDC24
CDC25
CDC26
CDC27
CDC28
CDC3
CDC31
CDC33
CDC34
CDC36
CDC37
CDC39
CDC4
CDC40
CDC42
CDC43
CDC45
CDC46
CDC47
CDC48
CDC5
CDC50
CDC53
CDC54
CDC55
CDC6
CDC60
CDC7
CDC73
CDC8
CDC9
CDC91
CDH1
CDS1
CEF1
CEG1
CEM1
CEP3
CET1
CFT1
CFT2
CHA1
CHA4
CHC1
CHD1
CHL1
CHL4
CHO2
CHS1
CHS2
CHS3
CHS6
CIK1
CIN1
CIN5
CIN8
CIS1
CIS3
CIT1
CIT2
CIT3
CKA1
CKA2
CKB1
CKB2
CKI1
CLA4
CLB1
CLB2
CLB3
CLB4
CLB5
CLB6
CLC1
CLG1
CLN1
CLN2
CLN3
CLP1
CLU1
CMD1
CMK1
CMK2
CMP2
CNA1
CNE1
CNM67
CNS1
COF1
COP1
COQ1
COQ2
COQ3
COQ4
COQ6
COR1
COS1
COS10
COS12
COS5
COS6
COS7
COS8
COT1
COX10
COX11
COX12
COX15
COX18
COX4
COX5A
COX5B
COX9
CPA1
CPA2
CPR3
CPR6
CPR7
CPR8
CPS1
CRD1
CRM1
CRN1
CRS5
CRZ1
CSE1
CSE2
CSE4
CSG2
CSI2
CTA1
CTF13
CTF18
CTF19
CTF4
CTH1
CTK1
CTK2
CTK3
CTP1
CTR1
CTR2
CTR3
CTR86
CTR9
CTS1
CTT1
CUE1
CUP2
CUS1
CUS2
CWH41
CWP1
CYB2
CYC2
CYC3
CYC8
CYP2
CYP5
CYR1
CYS3
CYS4
CYT1
CYT2
DAK1
DAK2
DAL1
DAL2
DAL3
DAL4
DAL5
DAL7
DAL80
DAL81
DAL82
DAN1
DAP2
DAT1
DBF2
DBF20
DBF4
DBP1
DBP10
DBP2
DBP3
DBP5
DBP6
DBP7
DBP8
DBP9
DBR1
DCD1
DCG1
DCP1
DDC1
DDI1
DDR48
DED1
DED81
DEG1
DEP1
DFG10
DFG16
DFG5
DFR1
DHH1
DHS1
DIB1
DIC1
DIE2
DIG1
DIG2
DIM1
DIN7
DIP2
DIP5
DIS3
DIT1
DIT2
DJP1
DLD1
DMC1
DNA2
DNA43
DNL4
DNM1
DOA1
DOA4
DOC1
DOG1
DOG2
DOM34
DOS2
DOT5
DOT6
DPB11
DPB2
DPB3
DPH2
DPH5
DPL1
DPM1
DPP1
DPS1
DRS1
DRS2
DSK2
DSS4
DST1
DUN1
DUO1
DUR3
DUT1
DYN1
DYS1
EBS1
ECM1
ECM10
ECM11
ECM13
ECM14
ECM15
ECM16
ECM17
ECM18
ECM2
ECM21
ECM22
ECM23
ECM25
ECM27
ECM29
ECM3
ECM30
ECM32
ECM33
ECM37
ECM38
ECM39
ECM4
ECM40
ECM5
ECM7
ECM8
ECM9
EGD2
EGT2
EKI1
ELC1
ELM1
ELO1
EMP24
EMP47
EMP70
ENA1
ENA5
END3
ENO1
ENO2
ENP1
EPL1
EPT1
ERD1
ERD2
ERG1
ERG10
ERG11
ERG12
ERG2
ERG20
ERG24
ERG25
ERG3
ERG4
ERG5
ERG6
ERG7
ERG8
ERG9
ERO1
ERS1
ERV1
ERV25
ESA1
ESBP6
ESC1
ESC2
ESC4
ESP1
ESR1
ESS1
EST1
EST2
EUG1
EXG1
EXG2
EXO70
FAA1
FAA2
FAA3
FAA4
FAB1
FAD1
FAL1
FAP1
FAR1
FAR3
FAS1
FAS2
FAT1
FAT2
FBA1
FBP1
FBP26
FCP1
FCY1
FCY2
FCY21
FEN2
FET3
FET4
FET5
FHL1
FIG1
FIG2
FIG4
FIP1
FIR1
FKB2
FKH1
FKH2
FKS1
FKS3
FLO1
FLO10
FLR1
FLX1
FMC1
FMS1
FOB1
FOL2
FOX2
FPR4
FPS1
FRE1
FRE2
FRE3
FRE4
FRE5
FRE6
FRE7
FRS1
FRS2
FTR1
FUI1
FUM1
FUN11
FUN12
FUN14
FUN19
FUN20
FUN21
FUN26
FUN30
FUN31
FUN34
FUN9
FUR4
FUS1
FUS2
FUS3
FZF1
FZO1
GAA1
GAC1
GAL1
GAL10
GAL11
GAL2
GAL3
GAL4
GAL7
GAL80
GAL83
GAP1
GAR1
GAS1
GAT1
GBP2
GCD1
GCD10
GCD11
GCD14
GCD2
GCD6
GCD7
GCN1
GCN2
GCN20
GCN3
GCN4
GCN5
GCR1
GCR2
GCS1
GCV1
GCV2
GCV3
GCY1
GDA1
GDH1
GDH2
GDH3
GDI1
GDS1
GEA1
GEF1
GFA1
GIC1
GIF1
GIN4
GIP1
GIP2
GIS1
GIT1
GLC3
GLC7
GLC8
GLE1
GLE2
GLG1
GLG2
GLK1
GLN1
GLN3
GLN4
GLO1
GLO2
GLO3
GLO4
GLR1
GLT1
GLY1
GND1
GND2
GNP1
GOG5
GOS1
GPA1
GPA2
GPD1
GPD2
GPH1
GPI1
GPI10
GPI2
GPI8
GPM1
GPM2
GPM3
GRD19
GRE1
GRE2
GRE3
GRF10
GRR1
GRS1
GSC2
GSF2
GSG1
GSH1
GSH2
GSY1
GSY2
GTR1
GTS1
GTT1
GTT2
GUA1
GUF1
GUT1
GUT2
GYP1
GYP6
GYP7
GZF3
HAC1
HAL1
HAL5
HAM1
HAP1
HAP2
HAP3
HAP4
HAP5
HAS1
HAT1
HAT2
HBS1
HCA4
HCM1
HCS1
HDA1
HEM1
HEM12
HEM13
HEM14
HEM15
HEM2
HEM3
HEM4
HES1
HEX3
HFA1
HFI1
HFM1
HGH1
HHF1
HHF2
HHO1
HHT1
HHT2
HIF1
HIG1
HIP1
HIR1
HIR2
HIR3
HIS1
HIS2
HIS3
HIS4
HIS5
HIS6
HIS7
HIT1
HKR1
HLJ1
HMG1
HMG2
HMGS
HMI1
HMS1
HMT1
HNM1
HNT2
HO
HOC1
HOG1
HOL1
HOM2
HOM3
HOM6
HOP1
HOP2
HOR2
HOS1
HOS2
HOS3
HPA2
HPC2
HPR1
HPR5
HPT1
HRB1
HRD1
HRD3
HRP1
HRR25
HRT1
HRT2
HSC82
HSF1
HSH49
HSL1
HSL7
HSN1
HSP104
HSP26
HSP30
HSP42
HSP60
HSP78
HSP82
HST1
HST2
HST3
HST4
HTA1
HTA2
HTA3
HTB1
HTB2
HTS1
HVG1
HXK1
HXK2
HXT1
HXT10
HXT11
HXT12
HXT13
HXT14
HXT17
HXT2
HXT3
HXT4
HXT5
HXT8
HYM1
HYS2
IAH1
ICL1
ICL2
IDH1
IDH2
IDI1
IDP1
IDP2
IDP3
IDS2
IFH1
IFM1
IKI1
IKI3
IKS1
ILS1
ILV1
ILV2
ILV3
ILV5
ILV6
IME1
IME2
IME4
IMG1
IMG2
IMH1
IMP1
IMP2
IMP2'
INO1
INO2
INO4
INO80
INP51
INP52
INP53
INP54
IPL1
IPP1
IPT1
IQG1
IRA1
IRA2
IRE1
IRR1
IRS4
ISC10
ISF1
ISM1
ISR1
ISW1
ISW2
ISY1
ITR1
ITR2
IXR1
JAC1
JEM1
JEN1
JNM1
JSN1
KAP104
KAP123
KAP95
KAR1
KAR2
KAR3
KAR4
KAR5
KAR9
KCS1
KEL1
KEL2
KEL3
KEM1
KES1
KEX1
KEX2
KGD1
KGD2
KIM2
KIM3
KIN1
KIN2
KIN28
KIN3
KIN4
KIN82
KIP1
KIP2
KIP3
KKQ8
KNH1
KNS1
KRE11
KRE2
KRE5
KRE6
KRR1
KRS1
KSP1
KSS1
KTI12
KTR1
KTR2
KTR3
KTR4
KTR5
KTR6
KTR7
LAG1
LAG2
LAP3
LAP4
LAS1
LAS17
LAT1
LCB1
LCB2
LCB3
LCB4
LCB5
LCP5
LEE1
LEO1
LEU1
LEU2
LEU3
LEU4
LHS1
LIF1
LIP5
LOS1
LPD1
LPP1
LRE1
LRG1
LRS4
LSC1
LSC2
LST7
LST8
LTE1
LTP1
LTV1
LYP1
LYS1
LYS12
LYS14
LYS2
LYS20
LYS21
LYS4
LYS5
LYS9
MAD1
MAD2
MAD3
MAG1
MAK10
MAK11
MAK16
MAK3
MAK32
MAK5
MAL31
MAL32
MAL33
MAP1
MAP2
MAS1
MAS2
MAS6
MBA1
MBP1
MBR1
MCD1
MCD4
MCK1
MCM1
MCM16
MCM2
MCM21
MCM22
MCM3
MCM6
MCR1
MDH1
MDH2
MDJ1
MDL1
MDL2
MDM1
MDM10
MDM12
MDM20
MDR1
MDS3
MEC3
MED2
MED4
MED6
MED7
MED8
MEF1
MEF2
MEI5
MEK1
MEP1
MEP2
MEP3
MER1
MES1
MET1
MET10
MET12
MET13
MET16
MET17
MET18
MET2
MET22
MET28
MET3
MET30
MET32
MET4
MET6
MET8
MEU1
MEX67
MF(ALPHA)2
MFA2
MFT1
MGA1
MGA2
MGE1
MGM1
MGM101
MGT1
MHP1
MID1
MIF2
MIG1
MIG2
MIH1
MIP1
MIP6
MIR1
MIS1
MKC7
MKK1
MKK2
MKS1
MKT1
MLH1
MLP1
MLS1
MMD1
MMM1
MMS2
MMS21
MMT1
MMT2
MNE1
MNN1
MNN10
MNN11
MNN4
MNN9
MNR2
MNS1
MOD5
MOG1
MOT1
MOT2
MOT3
MPA43
MPD1
MPP10
MPS1
MPT1
MPT5
MRE11
MRF1
MRF1'
MRK1
MRP1
MRP13
MRP17
MRP2
MRP20
MRP21
MRP4
MRP49
MRP51
MRP7
MRP8
MRPL11
MRPL13
MRPL16
MRPL20
MRPL25
MRPL27
MRPL28
MRPL31
MRPL32
MRPL35
MRPL36
MRPL37
MRPL39
MRPL4
MRPL40
MRPL44
MRPL6
MRPL7
MRPL8
MRPL9
MRPS28
MRPS5
MRPS9
MRS1
MRS2
MRS3
MRS5
MRS6
MRT4
MSB1
MSB2
MSB3
MSB4
MSD1
MSE1
MSF1
MSF1'
MSG5
MSH1
MSH2
MSH3
MSH4
MSH5
MSH6
MSI1
MSK1
MSL1
MSL5
MSM1
MSN1
MSN2
MSN4
MSN5
MSO1
MSP1
MSR1
MSS1
MSS11
MSS116
MSS4
MSS51
MST1
MSU1
MSW1
MSY1
MTD1
MTF1
MTF2
MTH1
MTR10
MTR2
MTR3
MTR4
MUB1
MUC1
MUD1
MUD13
MUD2
MUM2
MUP1
MUP3
MUQ1
MVD1
MVP1
MYO1
MYO2
MYO3
MYO4
MYO5
NAB2
NAB3
NAM2
NAM7
NAM8
NAM9
NAP1
NAT1
NAT2
NBN1
NBP1
NBP2
NBP35
NCA2
NCA3
NCB2
NCE102
NCE103
NCE4
NCP1
NDC1
NDI1
NDJ1
NDT80
NEO1
NFI1
NFS1
NGG1
NGR1
NHA1
NHP10
NHP2
NHX1
NIC96
NIF3
NIP1
NIP100
NIP29
NIP7
NIT1
NIT2
NIT3
NMD2
NMD3
NMD4
NMD5
NMT1
NNF1
NOP1
NOP2
NOP4
NOT3
NPL3
NPL4
NPL6
NPR1
NPR2
NPT1
NRD1
NRK1
NRP1
NSP1
NSR1
NTA1
NTC20
NTF2
NTG1
NTG2
NTH2
NUC1
NUD1
NUF1
NUF2
NUM1
NUP1
NUP100
NUP116
NUP120
NUP133
NUP145
NUP157
NUP159
NUP170
NUP188
NUP2
NUP42
NUP49
NUP57
NUP82
NUP84
NUP85
NUT1
NUT2
OCH1
OGG1
OLE1
OM45
OPI1
OPI3
OPY1
OPY2
ORC1
ORC3
ORC4
ORC5
ORC6
ORM1
OSH1
OSM1
OST1
OST2
OST3
OST4
OXA1
OYE2
OYE3
PAC1
PAC10
PAC11
PAC2
PAD1
PAF1
PAK1
PAM1
PAN1
PAN2
PAN3
PAP1
PAT1
PAU2
PBN1
PBP1
PBP2
PBS2
PCA1
PCF11
PCH2
PCK1
PCL1
PCL10
PCL2
PCL5
PCL6
PCL7
PCL8
PCM1
PCT1
PDA1
PDB1
PDC1
PDC2
PDC5
PDC6
PDE1
PDE2
PDI1
PDR1
PDR10
PDR11
PDR12
PDR13
PDR15
PDR3
PDR5
PDS1
PDS5
PDX1
PDX3
PEA2
PEP1
PEP12
PEP3
PEP4
PEP5
PEP7
PEP8
PES4
PET100
PET111
PET112
PET117
PET122
PET123
PET127
PET130
PET18
PET191
PET309
PET494
PET54
PET56
PET8
PET9
PEX1
PEX10
PEX11
PEX12
PEX13
PEX14
PEX15
PEX17
PEX19
PEX2
PEX3
PEX4
PEX5
PEX6
PEX7
PEX8
PFD1
PFK1
PFK2
PFK26
PFK27
PGD1
PGI1
PGK1
PGM1
PGM2
PGU1
PHA2
PHB1
PHB2
PHD1
PHO11
PHO13
PHO23
PHO3
PHO4
PHO5
PHO8
PHO80
PHO81
PHO84
PHO85
PHO86
PHO87
PHO88
PHR1
PIB1
PIF1
PIG1
PIG2
PIK1
PIM1
PIP2
PIR3
PIS1
PKA3
PKC1
PKH1
PKH2
PLC1
PMA1
PMA2
PMC1
PMD1
PMI40
PMR1
PMS1
PMT1
PMT2
PMT3
PMT4
PMT5
PMU1
PNT1
POB3
POL1
POL12
POL2
POL30
POL32
POL4
POL5
POM152
POP1
POP2
POP3
POP4
POP5
POP6
POP7
POP8
POR1
POS5
POT1
POX1
PPA1
PPA2
PPG1
PPH21
PPH22
PPH3
PPQ1
PPR1
PPT1
PPT2
PPZ1
PPZ2
PRB1
PRC1
PRD1
PRE1
PRE10
PRE2
PRE3
PRE4
PRE5
PRE6
PRE7
PRE8
PRI1
PRI2
PRK1
PRO1
PRO2
PRO3
PRP11
PRP12
PRP16
PRP18
PRP19
PRP2
PRP22
PRP24
PRP28
PRP31
PRP39
PRP4
PRP40
PRP42
PRP43
PRP5
PRP6
PRP8
PRP9
PRS1
PRS2
PRS3
PRS4
PRT1
PRY1
PRY3
PSA1
PSD1
PSD2
PSE1
PSP1
PSP2
PTA1
PTC1
PTC2
PTC3
PTK1
PTK2
PTM1
PTP1
PTP2
PTP3
PTR2
PTR3
PUB1
PUP1
PUP2
PUP3
PUS1
PUS2
PUS4
PUT1
PUT2
PUT3
PUT4
PWP1
PWP2
PXA1
PXA2
PYC1
PYC2
PYK2
PZF1
QCR2
QCR6
QCR7
QRI1
QRI2
QRI5
QRI7
QRI8
RAD1
RAD10
RAD14
RAD16
RAD17
RAD18
RAD2
RAD23
RAD24
RAD26
RAD27
RAD28
RAD3
RAD30
RAD4
RAD5
RAD50
RAD51
RAD52
RAD53
RAD54
RAD55
RAD57
RAD59
RAD6
RAD7
RAD9
RAM1
RAM2
RAP1
RAS2
RAT1
RBK1
RBL2
RCK1
RCK2
RCS1
RDH54
RDI1
REB1
REC102
REC104
REC107
REC114
RED1
REF2
REG1
REG2
RET1
RET2
RET3
REV1
REV3
REV7
RFA1
RFA2
RFA3
RFC1
RFC2
RFC3
RFC4
RFC5
RFT1
RFX1
RGA1
RGA2
RGP1
RGR1
RGT1
RGT2
RHC18
RHK1
RHO1
RHO3
RHO4
RHR2
RIB1
RIB2
RIB3
RIB4
RIB5
RIB7
RIC1
RIF1
RIF2
RIM101
RIM11
RIM13
RIM15
RIM2
RIM9
RIO1
RIP1
RIS1
RIT1
RKI1
RLF2
RLM1
RLP7
RLR1
RME1
RML2
RMS1
RNA1
RNA14
RNA15
RNC1
RNH1
RNH35
RNH70
RNP1
RNR1
RNR2
RNR3
RNR4
RNT1
ROD1
ROK1
ROM1
ROT1
ROT2
ROX1
ROX3
RPA12
RPA190
RPA34
RPA43
RPA49
RPB10
RPB2
RPB3
RPB4
RPB5
RPB8
RPB9
RPC19
RPC25
RPC31
RPC34
RPC40
RPC53
RPC82
RPD3
RPG1
RPH1
RPI1
RPL10
RPL11A
RPL11B
RPL12A
RPL12B
RPL13A
RPL13B
RPL14A
RPL14B
RPL15A
RPL15B
RPL16B
RPL18A
RPL18B
RPL19B
RPL20A
RPL20B
RPL21A
RPL21B
RPL22A
RPL24A
RPL24B
RPL26A
RPL26B
RPL27A
RPL27B
RPL28
RPL2B
RPL3
RPL30
RPL32
RPL33A
RPL33B
RPL35A
RPL35B
RPL36A
RPL37A
RPL37B
RPL38
RPL40A
RPL40B
RPL43A
RPL5
RPL6A
RPL6B
RPL7A
RPL8A
RPL8B
RPL9A
RPL9B
RPM2
RPN1
RPN10
RPN11
RPN12
RPN2
RPN3
RPN4
RPN5
RPN6
RPN7
RPN8
RPN9
RPO21
RPO26
RPO31
RPO41
RPP0
RPP1
RPR2
RPS0A
RPS10A
RPS11A
RPS11B
RPS12
RPS13
RPS14A
RPS14B
RPS15
RPS16A
RPS16B
RPS17B
RPS18A
RPS18B
RPS19A
RPS19B
RPS1A
RPS1B
RPS2
RPS20
RPS21A
RPS21B
RPS22A
RPS22B
RPS23A
RPS23B
RPS24A
RPS25B
RPS26A
RPS26B
RPS27A
RPS27B
RPS29A
RPS31
RPS4A
RPS6A
RPS6B
RPS7A
RPS7B
RPS8B
RPS9B
RPT1
RPT2
RPT3
RPT4
RPT5
RPT6
RRD1
RRD2
RRN11
RRN3
RRN5
RRN6
RRN7
RRN9
RRP1
RRP3
RRP42
RRP43
RRP45
RRP46
RRP5
RRP6
RRP7
RSC1
RSC2
RSC4
RSC6
RSC8
RSE1
RSP5
RSR1
RTA1
RTF1
RTG1
RTG2
RTG3
RTS1
RTS2
RUB1
RUD3
RVS161
RVS167
SAC1
SAC2
SAC3
SAC6
SAC7
SAE2
SAG1
SAH1
SAM1
SAM2
SAN1
SAP1
SAP155
SAP18
SAP190
SAP4
SAR1
SAS10
SAS2
SAS3
SAS4
SAS5
SAT4
SBA1
SBE2
SBE22
SBP1
SCC2
SCC3
SCD5
SCD6
SCH9
SCJ1
SCL1
SCO1
SCO2
SCP1
SCP160
SCS3
SCS7
SCT1
SCY1
SDC25
SDH1
SDH2
SDH3
SDH4
SDL1
SDS22
SDS3
SEC1
SEC10
SEC11
SEC12
SEC13
SEC14
SEC15
SEC16
SEC17
SEC18
SEC2
SEC20
SEC21
SEC22
SEC23
SEC24
SEC26
SEC27
SEC28
SEC3
SEC31
SEC4
SEC5
SEC53
SEC59
SEC6
SEC61
SEC62
SEC63
SEC65
SEC66
SEC7
SEC72
SEC8
SEC9
SED4
SED5
SEF1
SEH1
SEN1
SEN2
SEN34
SEN54
SEO1
SER1
SER2
SES1
SET1
SET2
SFA1
SFH1
SFI1
SFL1
SFP1
SGA1
SGD1
SGE1
SGS1
SGT1
SGT2
SGV1
SHE1
SHE10
SHE3
SHE4
SHE9
SHM1
SHM2
SHP1
SHR3
SHR5
SHY1
SIC1
SIK1
SIM1
SIN3
SIN4
SIP1
SIP18
SIP2
SIP3
SIP4
SIR1
SIR2
SIR3
SIR4
SIS1
SIS2
SIT4
SIW14
SKI2
SKI3
SKI6
SKI8
SKM1
SKN1
SKN7
SKO1
SKP1
SKS1
SKT5
SKY1
SLA1
SLA2
SLC1
SLF1
SLG1
SLK19
SLN1
SLS1
SLT2
SLU7
SLY1
SLY41
SLZ1
SMB1
SMC1
SMC2
SMC3
SMC4
SMD1
SMD2
SMD3
SME1
SMF1
SMF2
SMI1
SMK1
SMM1
SMP1
SMP2
SMP3
SMT3
SMT4
SMY1
SMY2
SNC1
SNC2
SNF1
SNF11
SNF12
SNF2
SNF3
SNF4
SNF5
SNF6
SNF7
SNF8
SNG1
SNL1
SNM1
SNO1
SNO3
SNP1
SNQ2
SNT309
SNU114
SNU56
SNU71
SNZ1
SNZ3
SOD1
SOD2
SOF1
SOH1
SOK2
SOL1
SOL3
SOL4
SOV1
SPA2
SPB4
SPC2
SPC3
SPC42
SPC72
SPC97
SPC98
SPE1
SPE2
SPE3
SPE4
SPF1
SPH1
SPL2
SPO1
SPO12
SPO14
SPO16
SPO20
SPO7
SPP2
SPP41
SPR1
SPR28
SPR3
SPS1
SPS100
SPS18
SPS19
SPS2
SPS4
SPT10
SPT14
SPT15
SPT16
SPT2
SPT20
SPT21
SPT23
SPT3
SPT4
SPT5
SPT6
SPT7
SPT8
SQT1
SRA1
SRA3
SRB4
SRB5
SRB6
SRB8
SRD1
SRM1
SRN2
SRO4
SRO7
SRO9
SRP1
SRP101
SRP14
SRP21
SRP40
SRP68
SRP72
SRV2
SSA1
SSA2
SSA3
SSA4
SSB2
SSC1
SSD1
SSE1
SSE2
SSF1
SSF2
SSH1
SSH4
SSK1
SSK2
SSK22
SSL1
SSL2
SSM4
SSN2
SSN3
SSN8
SSO1
SSO2
SSP1
SSP120
SSP2
SSQ1
SST2
SSU1
SSU72
SSU81
SSY1
SSY5
STB1
STB2
STB3
STB4
STB5
STB6
STD1
STE11
STE12
STE13
STE14
STE18
STE2
STE20
STE23
STE24
STE3
STE4
STE5
STE50
STE6
STE7
STF2
STH1
STI1
STL1
STM1
STN1
STO1
STP1
STP2
STP22
STP3
STP4
STS1
STT3
STT4
STU1
STU2
STV1
SUA5
SUA7
SUB1
SUB2
SUC2
SUI1
SUI2
SUI3
SUL1
SUL2
SUM1
SUN4
SUP35
SUP45
SUR1
SUR2
SUR7
SUT1
SUV3
SVL3
SWE1
SWH1
SWI1
SWI3
SWI4
SWI5
SWI6
SWP1
SXM1
SYF1
SYF2
SYF3
SYG1
SYS1
SYT1
TAF145
TAF25
TAF40
TAF47
TAF60
TAF61
TAF67
TAF90
TAL1
TAP42
TAT2
TBF1
TCI1
TCM10
TCP1
TDH1
TDH2
TEA1
TEC1
TEF2
TEF4
TEL1
TEL2
TEM1
TEP1
TES1
TFA1
TFA2
TFB1
TFB2
TFB3
TFB4
TFC1
TFC3
TFC4
TFC5
TFC6
TFG1
TFG2
TFP1
TFP3
TFS1
TGL1
TGL2
THI2
THI3
THI4
THI6
THI7
THI80
THR1
THR4
THS1
TID3
TIF2
TIF3
TIF34
TIF35
TIF4631
TIF4632
TIF5
TIM17
TIM22
TIM44
TIM54
TIN1
TIP20
TIR1
TIR2
TIS11
TKL1
TKL2
TLG1
TLG2
TOF1
TOF2
TOK1
TOM1
TOM20
TOM37
TOM40
TOM7
TOM70
TOM71
TOP1
TOP2
TOP3
TOR1
TOR2
TPD3
TPI1
TPK3
TPM2
TPS1
TPS2
TPS3
TPT1
TRA1
TRF4
TRF5
TRK1
TRK2
TRL1
TRM1
TRP2
TRP3
TRP4
TRP5
TRR1
TRR2
TSA1
TSL1
TSM1
TTR1
TUB1
TUB2
TUB3
TUB4
TUP1
TWF1
TYE7
TYR1
TYS1
UBA1
UBA2
UBC1
UBC12
UBC13
UBC4
UBC5
UBC6
UBC8
UBC9
UBI4
UBP1
UBP11
UBP12
UBP13
UBP14
UBP15
UBP16
UBP2
UBP3
UBP5
UBP6
UBP7
UBP8
UBP9
UBR1
UBS1
UFD1
UFD2
UFD4
UFE1
UGA1
UGA3
UGA4
UGX2
ULA1
UME1
UME6
UMP1
UNG1
UPF3
URA1
URA10
URA2
URA3
URA4
URA5
URA6
URA7
URA8
URE2
URK1
USO1
USV1
UTH1
UTR1
UTR2
UTR4
VAC7
VAC8
VAM3
VAM6
VAM7
VAN1
VAP1
VAS1
VCX1
VID24
VIK1
VIP1
VMA13
VMA2
VMA21
VMA22
VMA4
VMA5
VMA6
VMA7
VMA8
VPH1
VPH2
VPS1
VPS13
VPS15
VPS16
VPS17
VPS21
VPS24
VPS27
VPS28
VPS29
VPS3
VPS30
VPS33
VPS34
VPS35
VPS36
VPS38
VPS4
VPS41
VPS45
VPS5
VPS8
VPS9
VRP1
VTI1
WBP1
WHI2
WHI3
WHI4
WSC2
WSC3
WSC4
WTM1
WTM2
XBP1
XDJ1
XRS2
YAE1
YAF1
YAK1
YAP1
YAP1801
YAP1802
YAP3
YAP5
YAP6
YAP7
YAR1
YAT1
YBT1
YCF1
YCK1
YCK2
YCK3
YDJ1
YEA4
YEF3
YET1
YGP1
YHC1
YHC3
YHM1
YHM2
YIF1
YIP1
YJU2
YJU3
YKT6
YLA1
YLF2
YMC2
YME1
YNT20
YOR1
YOX1
YPK1
YPK2
YPR1
YPS1
YPS4
YPS5
YPS6
YPS7
YPT1
YPT31
YPT32
YPT52
YPT53
YPT6
YRA1
YRB2
YRO2
YRR1
YSA1
YSC83
YSC84
YSH1
YSP3
YSR3
YSW1
YTA12
YTA7
YTH1
YTM1
YTP1
YUR1
YVH1
ZAP1
ZDS1
ZDS2
ZIP1
ZIP2
ZMS1
ZRC1
ZRT1
ZRT2
ZTA1
ZUO1
ZWF1

|  |  |  |
| --- | --- | --- |
| Primer Name | | |
| Target ORF/Gene  | |
| Primer Sequence  | |
| Validity  | |
| Regression Line  | |
